# Peptidoglycan maturation controls outer membrane protein assembly

**DOI:** 10.1038/s41586-022-04834-7

**Published:** 2022-06-15

**Authors:** Gideon Mamou, Federico Corona, Ruth Cohen-Khait, Nicholas G. Housden, Vivian Yeung, Dawei Sun, Pooja Sridhar, Manuel Pazos, Timothy J. Knowles, Colin Kleanthous, Waldemar Vollmer

**Affiliations:** 1 Department of Biochemistry, South Parks Road, University of Oxford, Oxford, UK; 2 Centre for Bacterial Cell Biology, Biosciences Institute, Newcastle University, Newcastle upon Tyne, UK; 3 Structural Biology, Genentech, South San Francisco, CA USA; 4 School of Biosciences, University of Birmingham, Birmingham, UK; 5 Present Address: Genome Biology Unit, European Molecular Biology Laboratory, Heidelberg, Germany; 6 Present Address: Department of Molecular Biology, Center of Molecular Biology ‘Severo Ochoa’ (UAM-CSIC), Autonomous University of Madrid, Madrid, Spain

**Keywords:** Membrane structure and assembly, Cell growth

## Abstract

Linkages between the outer membrane of Gram-negative bacteria and the peptidoglycan layer are crucial for the maintenance of cellular integrity and enable survival in challenging environments^1–5^. The function of the outer membrane is dependent on outer membrane proteins (OMPs), which are inserted into the membrane by the β-barrel assembly machine^6,7^ (BAM). Growing *Escherichia coli* cells segregate old OMPs towards the poles by a process known as binary partitioning, the basis of which is unknown^8^. Here we demonstrate that peptidoglycan underpins the spatiotemporal organization of OMPs. Mature, tetrapeptide-rich peptidoglycan binds to BAM components and suppresses OMP foldase activity. Nascent peptidoglycan, which is enriched in pentapeptides and concentrated at septa^9^, associates with BAM poorly and has little effect on its activity, leading to preferential insertion of OMPs at division sites. The synchronization of OMP biogenesis with cell wall growth results in the binary partitioning of OMPs as cells divide. Our study reveals that Gram-negative bacteria coordinate the assembly of two major cell envelope layers by rendering OMP biogenesis responsive to peptidoglycan maturation, a potential vulnerability that could be exploited in future antibiotic design.

## Abstract (web summary)

Peptidoglycan stem peptides in the Gram-negative bacterial cell wall regulate the insertion of essential outer membrane proteins, thus representing a potential target for antibiotic design.

## Main

The insertion of outer membrane β-barrel proteins into the membrane of Gram-negative bacteria is catalysed by the BAM complex^6,7^, comprising the β-barrel protein BamA and four accessory lipoproteins, BamB, BamC, BamD and BamE^10–12^. BamA is essential for viability, which, in conjunction with its surface exposure, makes it a promising target for Gram-negative-specific antibiotics^13–16^. Much is known of the mechanism by which BamA catalyses the folding of OMPs in vitro^17–20^. By contrast, little is known about BamA-mediated OMP biogenesis in the asymmetric outer membrane of live bacteria. Some studies suggest that new OMPs are inserted preferentially in the mid-cell region^8,21,22^, whereas others suggest that OMPs are inserted throughout the membrane^23,24^. These contrasting views are confounded by recent super-resolution microscopy studies in fixed cells that show BamA clustered throughout the outer membrane^25^.

## BAM activity is linked to the cell cycle

**Fig. 1:**
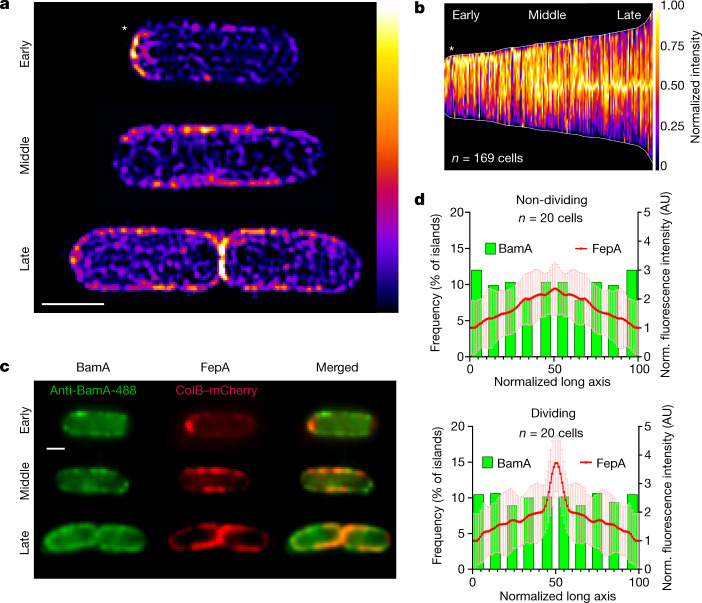
OMP biogenesis mirrors the cell cycle and not localization of BamA in the outer membrane of live *E. coli* cells. **a**, Biogenesis patterns for FepA, stained with ColB–GFP, following a 5-min induction (0.4% arabinose). Shown are fluorescence heat maps of individual cells representing different cell cycle stages, including a recently divided cell (early), an elongating cell (middle) and a dividing cell (late). **b**, Demograph showing normalized fluorescence intensity across multiple cells following a 5-min induction of FepA biogenesis. Cells are aligned to show the more intense pole at the top (white asterisk). **c**, Co-labelling of BamA (with BamA antibody) and FepA (with ColB–mCherry) at different cell cycle stages following 7 min of FepA induction. **d**, Comparison of FepA biogenesis regions (7 min induction; mean (red line) ± s.d. shaded region) with the distribution of BamA-containing islands (bars) (see also Extended Data Fig. 1), in dividing and non-dividing *E. coli* cells. Norm., normalized. Scale bars, 1 μm. AU, arbitrary units.

**Extended Data Fig. 1:**
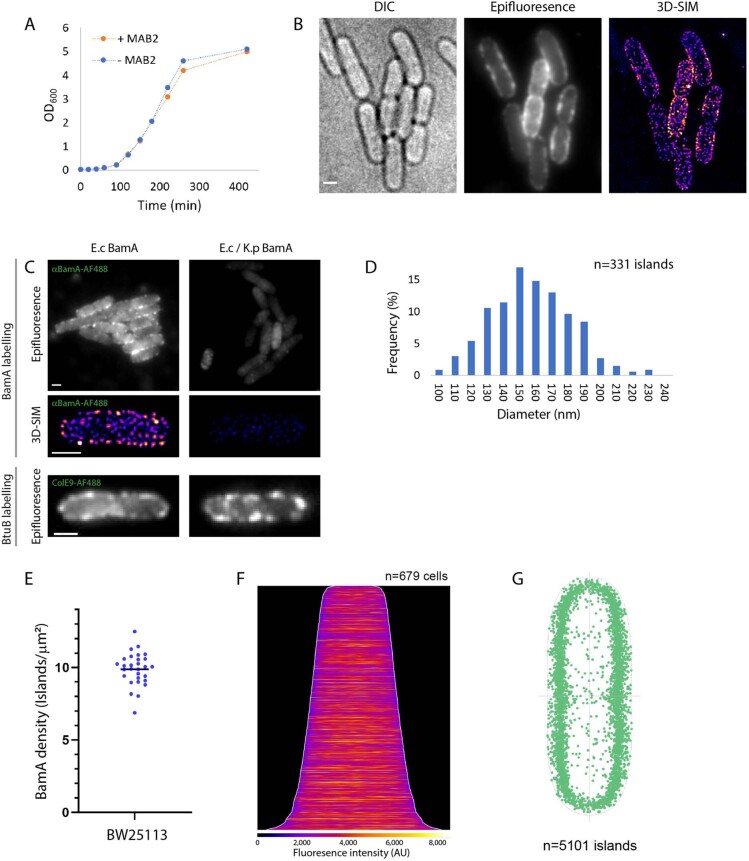
Surface labelling of BamA using a high-affinity monoclonal antibody. **(A)** Growth curves of *E. coli* BW25113 cells grown in LB with or without the MAB2 Fabs used for live cell labelling showing the Fabs have no effect on bacterial survival. **(B)** A field of view showing DIC, epifluorescence and 3D-SIM images of BamA labelled using αBamA^AF488^ Fabs. Scale bar, 1 μm. **(C)** BamA labelling by MAB2 is specific for *E. coli* BamA. BamA and BtuB, used as a control, labelling of the *E. coli* BW25113 (*E. coli* sequence) and a strain expressing a modified BamA β-barrel domain (*E. coli*/*K. pneumoniae* sequence). Shown are comparative images of cells labelled with αBamA^AF488^ or ColE9^AF488^. Scale bars, 1 μm. **(D)** Size distribution of BamA-containing islands demonstrating that the average island diameter is ~150 nm. **(E)** The density of BamA-containing islands on the surface of exponentially growing *E. coli* as measured by 3D-SIM (*n* = 30 cells). **(F)** Demograph of the fluorescence intensity across the long axis of multiple cells labelled with αBamA^AF488^, emphasising the even distribution of BamA molecules in the OM. **(G)** Integrated localization data from multiple cells showing BamA islands are distributed through the *E. coli* OM. The analysis is based on the same cells shown in panel F and represents different stages of the cell cycle.

**Extended Data Fig. 2:**
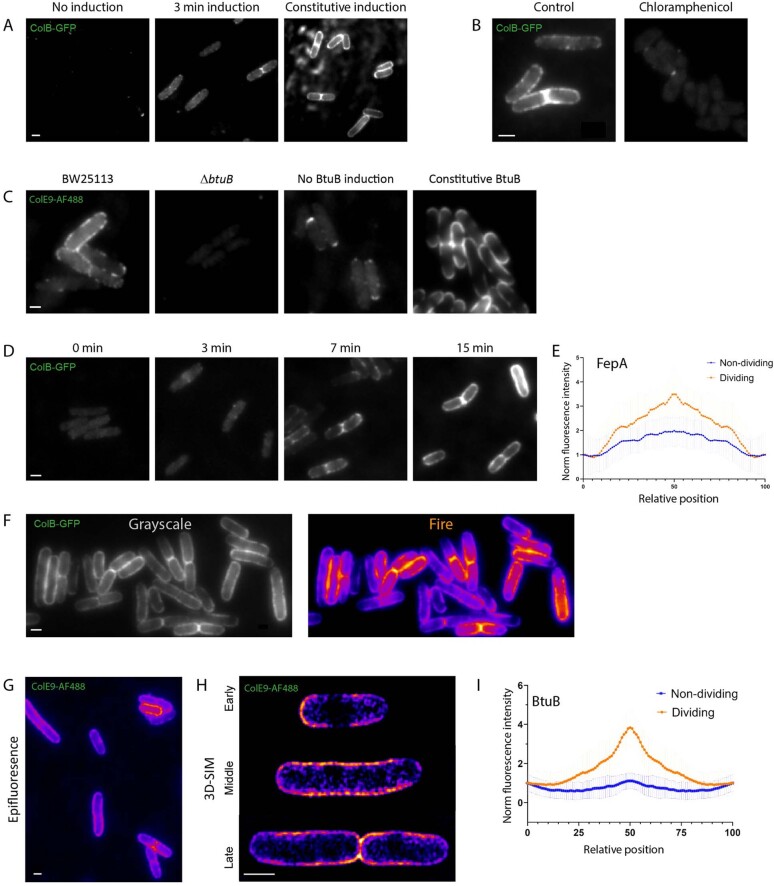
FepA and BtuB induction systems reveal the patterns of OMP biogenesis. **(A)** Epifluorescence images of FepA in an inducible expression strain (GM07). Shown are images of cells after different induction regimes. FepA expression was induced with 0.4% arabinose and the cells stained with ColB-GFP. **(B)** Images of FepA labelling after 7 min induction with or without chloramphenicol pre-treatment demonstrating that labelling is dependent on new protein synthesis. **(C)** Epifluorescence images of BtuB stained with ColE9^AF488^ under different induction regimes showing labelling is specific for BtuB. Expression was induced with 0.4% arabinose. **(D)** Timeline of FepA biogenesis in M9 media. Samples were taken and labelled with ColB-GFP at different time points after FepA induction. **(E)** The fluorescence distribution (± SD) of FepA labelling in dividing *vs*. non-dividing cells after 7 min induction (*n* = 25 cells for each condition). **(F)** A field of view showing grayscale and a corresponding fire heatmap of FepA labelling after 7 min induction. **(G)** Representative image of BtuB biogenesis after 5 min induction and staining with ColE9^AF488^. Shown is a heatmap of a field of view. **(H)** 3D-SIM projections of BtuB biogenesis after 5 min induction. The images represent different cell cycle stages. **(I)** The fluorescence distribution (± SD) of BtuB labelling in dividing vs non-dividing cells after 5 min induction (*n* = 30). Scale bar, 1 μm in all microscopy images shown.

**Extended Data Fig. 3:**
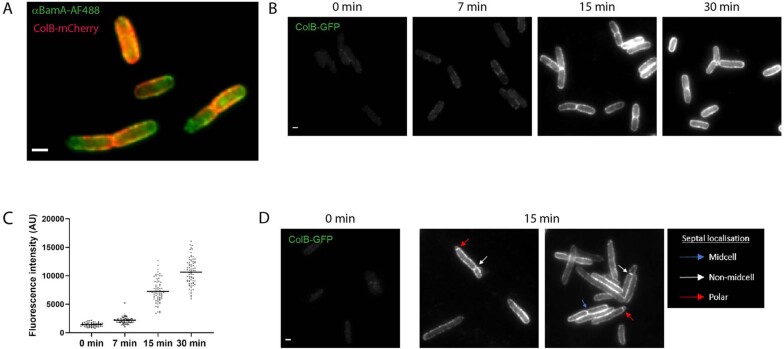
OMP biogenesis is septal biased and cell cycle dependent in native expression systems and division mutants. **(A)** Co-labelling of BamA and FepA with αBamA^AF488^ and ColB-mCherry, respectively, after 7 min induction of FepA biogenesis. Shown is an overlay of the BamA (*green*) and FepA (*red*) channels. **(B)** Timeline of FepA biogenesis following native chromosomal expression under iron limiting conditions. Samples were taken and labelled with ColB-GFP at different time points after the addition of 2,2 Bipyridyl to LB growth media. **(C)** Fluoresence intensity of cells shown in panel B. **(D)** Epifluorescence images of FepA in a Δ*minB* strain (PB114). Shown are images of cells before and after chromosomal induction with 2,2 Bipyridyl. Scale bars, 1 μm.

To determine where OMPs emerge and whether their insertion in the outer membrane is regulated, we first determined the localization of BamA in live *E. coli* cells. We visualized BamA using epifluorescence microscopy and 3D structured illumination microscopy (SIM) following labelling with a specific, high-affinity monoclonal Fab antibody^13^ (MAB2) that binds extracellular loop 6 in BamA with no impact on growth (Extended Data Fig. 1a–c). We found that BamA clusters into small (average diameter approximately 150 nm), uniformly distributed islands (8–10 per μm^2^) on the cell surface (Extended Data Fig. 1d–g and Supplementary Video 1), in agreement with data from fixed cells^25^. In contrast to another recent study on BamA localization, in which extensive cell permeabilization of fixed *E. coli* cells was needed for detection of BamA^26^, we saw no enrichment of BamA at division sites. We next investigated where newly synthesized OMPs appear on the cell surface in relation to this distribution of BamA. We focused on two TonB-dependent transporters (TBDTs), the siderophore transporter FepA and the vitamin B_12_ transporter BtuB. Both TBDTs were labelled with high-affinity, fluorescently labelled colicins that exploit these OMPs as receptors^8,27^. ColB was fused to mCherry or GFP to locate FepA and ColE9 was labelled with Alexa Fluor 488 (AF488) to locate BtuB. Both OMPs were expressed separately in *E. coli* from plasmids and induced with arabinose (Extended Data Fig. 2a–c). Time-course studies on FepA indicated that the OMP appeared on the bacterial surface approximately 3 min after induction (Extended Data Fig. 2d), and so all subsequent experiments included at least 3 min for induction. Within populations of cells that had been induced to express either FepA or BtuB, two clear zones of OMP biogenesis were observed; sites of cell division were predominant, yielding labelled septa and cells with unipolar labelling. We also observed OMPs on the long axis of cells (Fig. 1a,b and Supplementary Video 2). Little or no OMP biogenesis was observed at old poles (Extended Data Fig. 2e–i), in agreement with previous reports^8,21^. We next co-labelled BamA and FepA following a brief period of induction of the latter. Co-labelling revealed a clear divergence between BamA localization and the sites of OMP insertion. Whereas BamA was distributed uniformly across the entire cell surface, including the poles, OMP biogenesis was confined to the long axis of cells and division sites (Fig. 1c,d and Extended Data Fig. 3a). We observed a similar biogenesis pattern for FepA expressed from its endogenous promoter (Extended Data Fig. 3b,c), demonstrating that the phenomenon is not exclusive to plasmid-based expression of OMP genes. Intense OMP biogenesis at (aberrant) cell division sites near cell poles were also seen in a minicell mutant (∆*minB*) (Extended Data Fig. 3d), showing that enhanced OMP biogenesis is not limited to the mid-cell position but localizes at active cell division sites. These data demonstrate conclusively that BamA is not equally active in the outer membrane and that its OMP insertion activity is regulated in a cell cycle dependent manner.

## OMPs assemble at PG synthesis sites

**Fig. 2:**
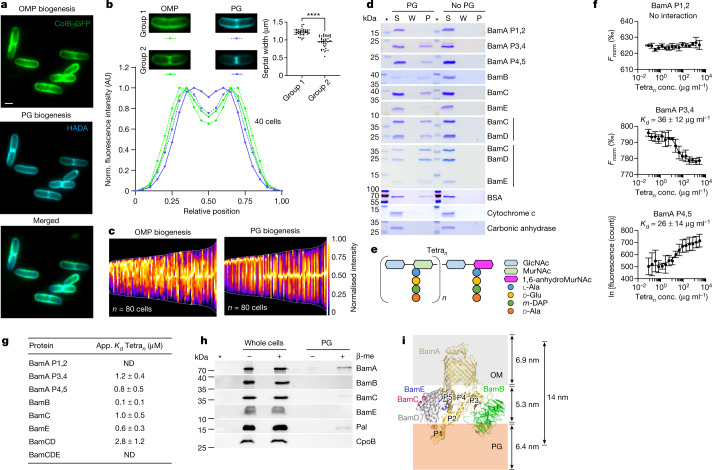
The cell wall has a pivotal role in OMP biogenesis. **a**, Co-labelling of PG (with HADA) and the OMP FepA (with ColB–GFP). PG staining and FepA induction were carried out simultaneously for 7 min. **b**, Fluorescence intensity profiles (main graph) of PG (HADA) and OMP biogenesis (ColB–GFP) across the septum of dividing cells at two different stages of septum formation (group 1 and group 2) (top left). Top right, the width of individual cells at the division plane. Statistical significance was calculated using two-tailed Student’s unpaired *t*-test (****P = 0.0001). **c**, Demographs comparing the normalized fluorescence distribution of FepA and PG biogenesis in multiple cells from experiments performed as in **a**. **d**, Interaction of BAM proteins with purified PG analysed by SDS–PAGE and Coomassie blue staining (gel source data is presented in Supplementary Fig. 1). S, supernatant fraction; W, wash fraction; P, pellet fraction. The asterisk indicates the protein size marker lane. **e**, Scheme of poly-disaccharide-tetrapeptide chains (Tetra_*n*_). GlcNAc, *N*-acetylglucosamine; *m*-DAP, *meso*-diaminopimelic acid; MurNAc, *N*-acetylmuramic acid. **f**, Interaction of BamA POTRA domain constructs with Tetra_*n*_ shown by MST. Data are mean ± s.d. (*n* = 3). Conc., concentration; *F*_norm_, normalized fluorescence. **g**, Apparent (app.) *K*_d_ values (mean ± s.d.; *n* = 3) for the interaction of Bam proteins or sub-complexes with Tetra_*n*_, measured by MST. ND, no interaction detected. **h**, Interaction of BamA and BamC with PG in *E. coli* MC1061 cells, detected with antibodies after in vivo cross-linking (blot source data are shown in Supplementary Fig. 1). Pal and CpoB were used as positive and negative controls, respectively. β-mercaptoethanol (β-me) releases cross-linked proteins from PG. **l**, Schematic depiction of the relative position of BAM^10^ (Protein Data Bank ID: 5AYW) in the cell envelope. The approximate width of PG (orange area), outer membrane (grey area) and outer membrane–PG distance were modelled on measurements from Matias et al. (2003)^53^. The position of each BAM subunit relative to the PG and how they change during the OMP folding cycle of BAM are not known.

**Extended Data Fig. 4:**
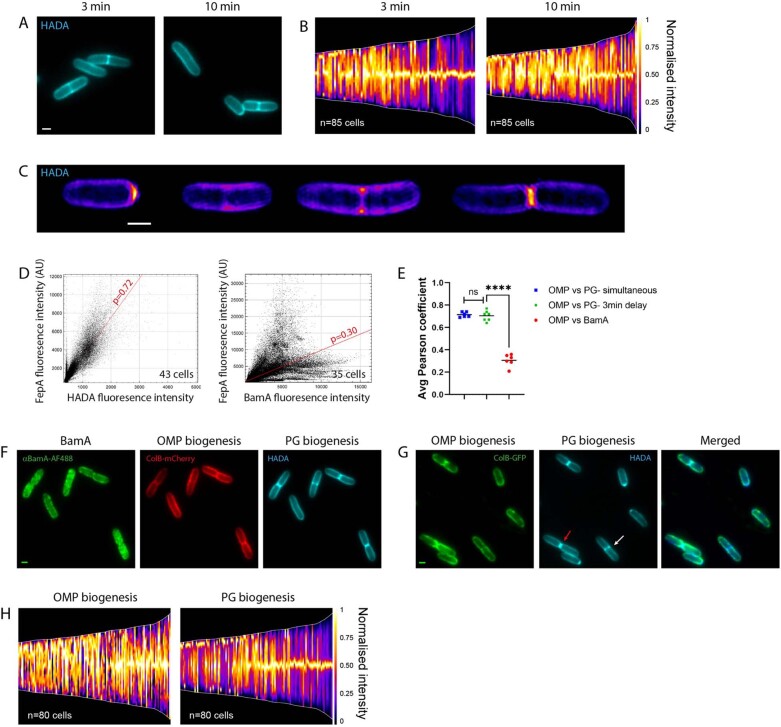
The spatiotemporal organization of cell wall biogenesis mirrors OMP biogenesis. **(A)** Images of *E. coli* BW25113 cells after incubation with the PG stain HADA for 3 or 10 min. **(B)** Demographs of the normalised fluorescence intensity across the long axis of multiple cells after 3 or 10 min incubation with HADA. Cells are aligned so the more intense pole appears at the top. The charts highlight the cell cycle dependent patterns of PG biogenesis. **(C)** 3D-SIM images of single *E. coli* cells after incubation with HADA for 10 min. *Left to right*, cells representing different stages of the cell cycle. **(D)** The fluorescence intensity of FepA vs HADA (*left*) or BamA (*right*) following simultaneous co-labelling. The representative pixel by pixel cytofluorograms of single images show that OMP biogenesis correlates better with PG biogenesis than BAM localization. **(E)** Jitter plot showing the correlation between FepA biogenesis and either PG biogenesis or BamA distribution. Shown are Pearson coefficients of single images and the average value. Statistical significance was calculated using two-tailed Student’s unpaired t-test (*P* = 0.0001). **(F)** Co-labelling of BamA, PG and OMP biogenesis. HADA was added 3 min after FepA induction and the total induction time was 10 min. **(G)** Co-labelling of PG and OMP biogenesis incorporating a time delay. HADA was added 3 min after FepA induction (stained with ColB-GFP). The total induction time was 10 min. The white/red arrows indicate cells from groups 1 and 2, respectively (see Fig. 2b). **(H)** Demographs comparing the normalised fluorescence distribution of FepA and PG biogenesis in multiple cells. Cells were treated as in panel G. Scale bars, 1 μm.

**Extended Data Fig. 5:**
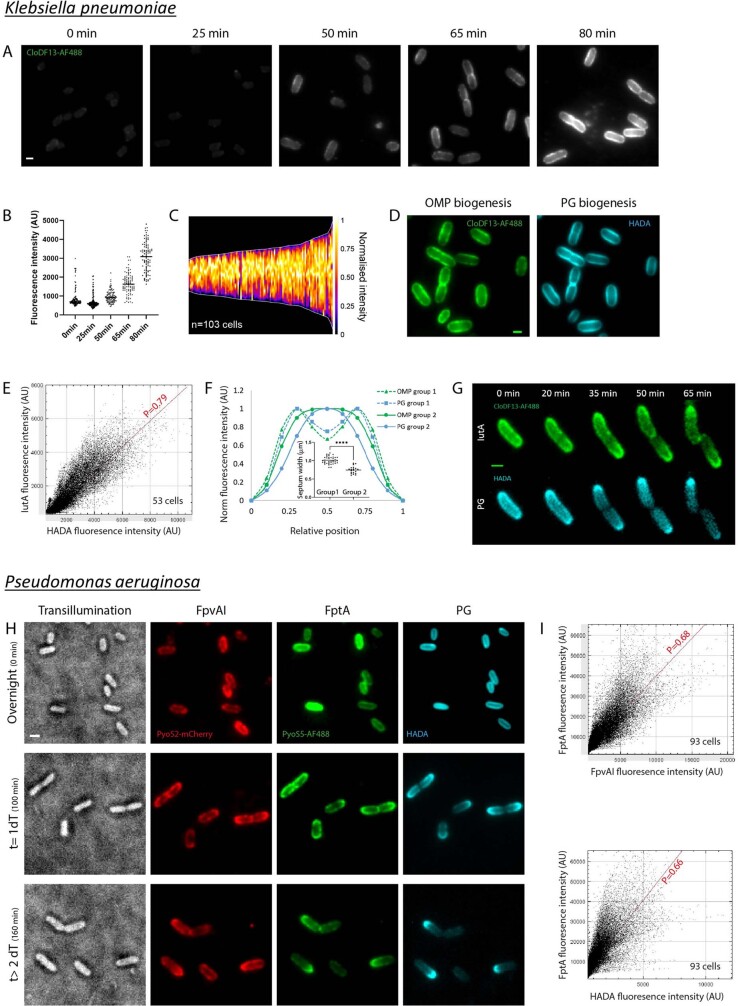
PG and OMP biogenesis are also coordinated in other Gram-negative bacterial species. **(A)** Timeline of IutA biogenesis in *K. pneumoniae*. IutA OMP expression was induced by transferring *K. pneumoniae* cells from overnight LB culture (0 min) into fresh M9. Samples were taken and labelled with CloDF13-AF488 at different time points after IutA induction. **(B)** Fluoresence intensity of cells shown in panel A. **(C)** Demograph showing the normalised fluorescence distribution of IutA in multiple cells. Cells were treated as in panel A. **(D)** Co-labelling of PG and OMP biogenesis 80 min after lutA induction as described in panel A. HADA was added 10 min before sample collection. **(E)** Pixel by pixel cytofluorogram of a single field of view after co-labelling as in panel D emphasising the correlation of PG and OMP fluorescence. **(F)** Comparison of the fluorescence intensity profiles of PG and OMP biogenesis in dividing *K. pneumoniae* cells. Shown are profiles of cells at two different stages of septum formation (Group 1/Group 2). Inset displays the width of individual cells at the designated division plane. n_early_ = 40 cells, n_late_ = 20 cells. Statistical significance was calculated using two-tailed Student’s unpaired t-test (*P* = 0.0001). In the following panels polar displacement of OMPs and PG was used as a measure of their spatially coordinated insertion (see Fig. 4c & Methods). **(G)** Time lapse images of PG and OMP polar displacement emphasising coordination of these layers during growth of *K. pneumoniae*. Cells from an overnight culture were labelled and grown on M9 agar pads. Images were taken at the indicated time points. **(H)** Time course images of PG and OMP polar displacement during *P. aeruginosa* growth. Cells from an overnight M9 culture were labelled with HADA and resuspended in fresh LB+FeCl_3_ to suppress expression of the OMPs FpvAI and FptA. Samples were taken at the indicated time points and each OMP labelled with PyoS2-mCherry and PyoS5-AF488, respectively. **(I)** Pixel by pixel cytofluorogram of a single field of view after co-labelling as in panel H. Charts demonstrate the co-localisation of both OMPs with one another (*top*) and with the old PG (*bottom*). Scale bar, 1 μm in all microscopy images shown.

Peptidoglycan (PG) is made of glycan chains connected by short peptides and forms a thin, net-like layer called sacculus underneath the outer membrane. The patterns of OMP biogenesis are reminiscent of those seen previously for PG in *E. coli*^28,29^. We therefore investigated the correlation between PG and OMP biogenesis through co-labelling experiments. PG biogenesis was imaged following incorporation of the fluorescent d-amino acid 7-hydroxycoumarincarbonylamino-d-alanine^30^ (HADA) (Extended Data Fig. 4a–c and Supplementary Video 3) and OMP biogenesis was imaged by labelling induced FepA with ColB–GFP. Cells were incubated for 7 min with both HADA and arabinose, the latter to induce *fepA* expression. Cells were then fixed and surface-exposed FepA was labelled with ColB–GFP. Similar patterns of fluorescence labelling were observed, with particularly strong labelling for both PG and OMP at division sites (Fig. 2a–c). Co-localization analysis of fluorophore pixel intensity across cells also revealed a strong correlation between PG and OMP fluorescence and this correlation was significantly greater than that for BamA and OMP fluorescence (Extended Data Fig. 4d,e), a conclusion that was confirmed by a triple-label experiment (Extended Data Fig. 4f and Supplementary Video 4). Closer inspection of new OMP and PG labelling at division septa revealed that they segregated into two populations; those with both PG and OMP labelling (group 2) and those with only PG labelling (group 1) (Fig. 2b). There were no cells with only OMP labelling at division sites. Fluorescence scans across each cell type and cell population demographs (Fig. 2b,c and Methods) demonstrated that PG biogenesis always appeared more advanced than OMP biogenesis. In addition, septal widths were narrower for group 2 cells compared to group 1 cells (Fig. 2b). Although the interpretation of these differences is complicated by the distinct, multi-step biosynthetic routes that result in label incorporation, they suggest that cell wall biogenesis precedes the emergence of OMPs at division sites. Dual-labelling experiments that included an additional (3 min) pre-induction period for *fepA* expression before the addition of HADA generated patterns of PG and OMP labelling similar to those without pre-induction (Extended Data Fig. 4e,g,h). Finally, we observed a similar correlation between OMP and PG biogenesis in *Klebsiella pneumoniae* (Extended Data Fig. 5a–g), including the emergence of PG before OMPs at the septum (Extended Data Fig. 5f). Spatial coordination between the two processes was also apparent in *Pseudomonas aeruginosa* (Extended Data Fig. 5h,i and Supplementary Video 5) suggesting that it is conserved for all Gram-negative bacteria. We conclude that the patterns of OMP and PG biogenesis closely mirror each other in exponentially growing cells, suggesting extensive crosstalk between these layers of the bacterial cell envelope.

## BAM proteins bind to PG

**Extended Data Fig. 6:**
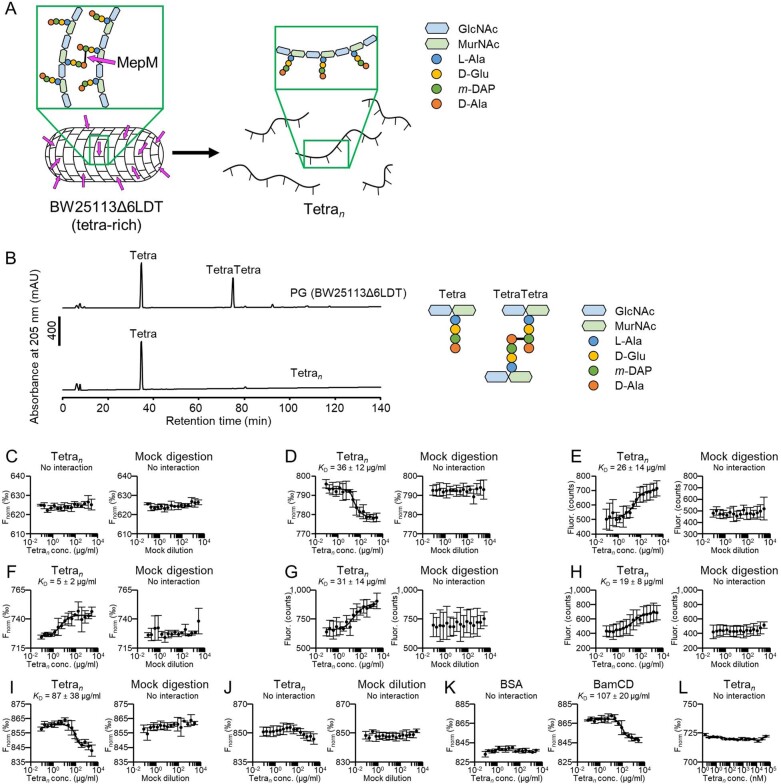
MST experiments for BAM proteins in the presence of Tetra_*n*_. **(A)** Generation of Tetra_*n*_ by enzymatic digestion of sacculi from BW25113Δ6LDT. The DD-endopeptidase MepM cleaves the DD-cross-links, generating soluble Tetra_*n*_ chains of variable length. **(B)** HPLC analysis of sacculi from BW25113Δ6LDT (*top*) and of Tetra_*n*_ (*bottom*), after digestion with the muramidase cellosyl to produce the disaccharide peptide subunits (muropeptides). Schematic representations of the chemical structures of muropeptides are shown on the right side. **(C)** MST experiment for BamA P1,2 and Tetra_*n*_. Fig. 2f shows that BamA P1,2 does not interact with Tetra_*n*_. Here, the right graph shows the lack of MST response in the control sample (mock PG digest without Tetra_*n*_). **(D)** MST experiment as in C but with BamA P3,4. BamA P3,4 interacts with Tetra_*n*_ (Fig. 2f). **(E)** MST experiment as in C but with BamA P4,5. BamA P4,5 interacts with Tetra_*n*_ (Fig. 2f). Further MST experiments were performed in the presence or absence of Tetra_*n*_ or mock PG digests for BamB **(F)**, BamC **(G)**, BamE **(H)**, BamCD **(I)** and BamCDE **(J)**. **(K)** Control experiments performed with fluorescent-labelled BSA or BamCD in the presence of Tetra_*n*_, showing that proteins do not generally bind to Tetra_*n*_ in MST experiments. **(L)** Control experiments performed with free fluorescent Red-NHS dye and no protein in the presence of Tetra_*n*_, showing that the fluorescent dye does not bind to Tetra_*n*_. Values shown in panels C-L are mean ± SD of three independent experiments. For source data see Supplementary Table 6.

We probed the link between OMP and PG biogenesis by first determining whether purified BAM proteins physically interact in pull-down experiments with PG isolated from wild-type *E. coli* MC1061^31,32^. We tested three fragments from BamA encompassing polypeptide-transport-associated (POTRA) domains 1 and 2 (BamA P1,2), 3 and 4 (BamA P3,4), and 4 and 5 (BamA P4,5), soluble versions of BamB, BamC and BamE, and BamCD and BamCDE complexes. We detected specific binding of all BAM proteins, except BamA P1,2, and of BamCD and BamCDE complexes to PG (Fig. 2d). We next tested the interaction of BAM proteins with soluble, uncross-linked disaccharide-tetrapeptide chains (Tetra_*n*_; Fig. 2e) by microscale thermophoresis (MST). We generated Tetra_*n*_ by digesting PG from *E. coli* BW25113Δ6LDT^30^, which exclusively contains tetrapeptides and tetra–tetra (4–3) cross-links, with the dd-endopeptidase MepM^33^ (Extended Data Fig. 6a,b). BamA P3,4 and BamA P4,5, but not BamA P1,2, interacted with Tetra_*n*_ (Fig. 2f). We also detected interaction of BamB, BamC, BamE and BamCD, but not BamCDE, with Tetra_*n*_, with apparent dissociation constant (*K*_d_) values between 0.15 and 2.8 µM (Fig. 2g and Extended Data Fig. 6). There were no changes in the MST responses in the absence of Tetra_*n*_ (that is, serial dilutions of mock MepM digestion without PG) (Extended Data Fig. 6). We conclude that BamA, BamB, BamC, BamE and BamCD bind specifically to Tetra_*n*_ chains of PG. To test whether BAM proteins interact with PG in *E. coli* cells, we treated *E. coli* MC1061 cultures with the chemical cross-linker 3,3′-dithiobis(sulfosuccinimidyl propionate) (DTSSP), followed by isolation of PG, reversal of cross-linking and detection of Bam proteins by SDS–PAGE with specific antibodies^34^. We found that BamA and BamC were cross-linked to PG isolated from DTSSP-treated cells (Fig. 2h), whereas BamB and BamE were not cross-linked to PG. As expected, the method identified the known PG-binding outer membrane lipoprotein Pal^35^, whereas the lipoprotein CpoB, which does not interact with PG^36^, was not detected. These results indicate that BamA and BamC interact with PG in *E. coli* cells. BAM proteins are likely to be close to the cell wall on the basis of dimensions of the complex inferred from structural studies (Fig. 2i). However, their relative positions are likely to vary owing to the highly dynamic nature of the OMP folding cycle^37^, which may explain why binding of PG to some BAM components is detected in vitro but not in vivo.

## PG differentially affects BAM activity

**Fig. 3:**
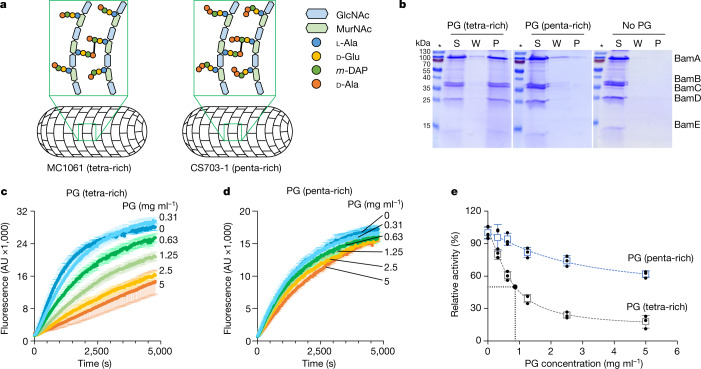
Nascent (pentapeptide-rich) and mature (tetrapeptide-rich) PG differentially affect BAM activity. **a**, Schematic representation of the PG structure in MC1061 and CS703-1, which are enriched in tetrapeptides (tetra-rich) and pentapeptides (penta-rich), respectively. **b**, Interaction of BamABCDE with tetrapeptide-rich and pentapeptide-rich sacculi in vitro after pull-down by SDS–PAGE and Coomassie blue staining (gel source data are shown in Supplementary Fig. 1). No PG, negative control without PG. **c**–**e**, Dose-dependent effects of tetrapeptide-rich (left) and pentapeptide-rich (right) PG on BAM-mediated OmpT assembly in vitro. Data are mean ± s.d. of three independent experiments.

**Extended Data Fig. 7:**
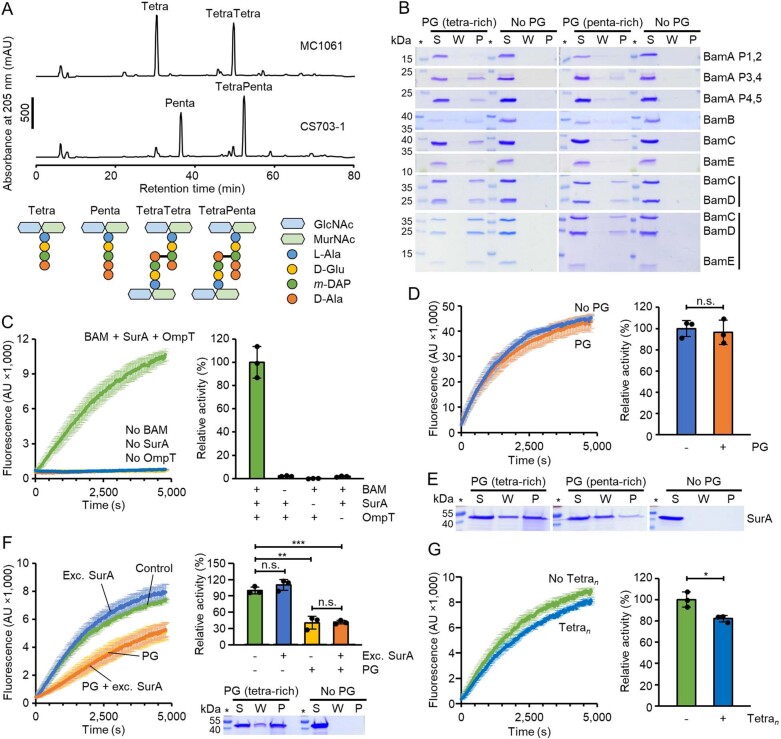
Controls for PG pull-down experiments and BAM activity assays performed with tetrapeptide-rich and pentapeptide-rich PG. **(A)** HPLC muropeptide analysis of tetrapeptide-rich PG from MC1061 and pentapeptide-rich PG from CS703-1. Schematic representation of the main muropeptides are shown. **(B)** PG pull-down assays for BAM proteins performed in the presence of tetrapeptide-rich PG (MC1061) or pentapeptide-rich PG (CS703-1), analysed by SDS-PAGE and Coomassie Blue staining (for gel source data, see SI Fig. 1). *S*, supernatant fraction; *W*, wash fraction; *P*, pellet fraction. Marker lanes are indicated by asterisks. Representative results from two independent replicates are shown. **(C)** BAM-mediated OmpT assembly *in vitro*. Control experiments without BAM (empty liposomes), OmpT or SurA are included. Values are mean ± SD of three independent experiments. **(D)** Effect of tetrapeptide-rich PG on OmpT activity. Sacculi did not reduce the cleavage of the fluorogenic peptide when added to reactions containing already folded OmpT (*n* = 3, *P* = 0.69). **(E)** Interaction of SurA with tetrapeptide-rich (MC1061) and pentapeptide-rich PG (CS703-1) *in vitro*, analysed by SDS-PAGE and Coomassie Blue staining (for gel source data, see SI Fig. 1). *S*, supernatant fraction; *W*, wash fraction; *P*, pellet fraction. **(F)** BAM activity measured in the presence of a 15 µM excess of SurA and tetrapeptide-rich PG, showing that SurA binding to PG is not a limiting factor for BAM activity *in vitro* (*n* = 3; control vs sample containing excess SurA: *P* = 0.21; control vs sample containing PG: *P* = 0.001; control vs sample containing PG + excess SurA: *P* = 0.0002; sample containing PG vs sample containing PG + excess SurA: *P* = 0.89. For gel source data, see SI Fig. 1). **(G)** Effect of Tetra_*n*_ on BAM-mediated OmpT assembly *in vitro* (*n* = 3, *P* = 0.02). Statistical significance of data shown in panels D, F and G was calculated using two-tailed Student’s unpaired t-test. Values are mean ± SD.

Sites of PG biosynthesis are characterized by a transient enrichment in pentapeptides, which are present in PG precursors but not in mature PG owing to the action of dd-carboxypeptidases^9^. We therefore tested whether differences in PG composition might account for the cell cycle dependence of OMP biogenesis. Specifically, we tested whether differences in stem peptide composition between mature (tetrapeptide-rich) and nascent (pentapeptide-rich) PG affected the BAM–PG interaction. We purified the complete BAM complex (BamABCDE) and tested its binding to different PG sacculi preparations. We used MC1061 as a source for mature PG and the multiple dd-carboxypeptidase mutant CS703-1^38,39^ as a source for pentapeptide-rich PG, mimicking nascent PG (Fig. 3a and Extended Data Fig. 7a). The BAM complex pulled down with tetrapeptide-rich PG, whereas we observed almost no binding to pentapeptide-rich PG (Fig. 3b). Purified BAM subunits also showed a reduced interaction with pentapeptide-rich PG compared with tetrapeptide-rich PG (Extended Data Fig. 7b). We conclude that BAM proteins have greater affinity for tetrapeptide-rich PG than pentapeptide-rich PG.

We next tested whether the composition of the peptides in PG affected BAM activity in vitro using an established assay that monitors the liposome incorporation of the β-barrel protease OmpT. The protease activity of folded OmpT is detected as fluorescence following cleavage of a self-quenched peptide substrate^40–42^ (Extended Data Fig. 7c). Addition of tetrapeptide-rich PG from MC1061 reduced BAM activity, whereas pentapeptide-rich PG from CS703-1 had little effect, consistent with the stronger binding of BAM proteins and the whole complex to PG (Fig. 3c–e). Control experiments indicated that the effects of PG on OMP folding activity were specific to BAM (Extended Data Fig. 7d–f). The effect of PG on BAM activity was dose-dependent, with a half-maximal effective concentration (EC_50_) of 0.86 mg ml^−1^ for tetrapeptide-rich PG and more than 5 mg ml^−1^ for pentapeptide-rich PG (Fig. 3c–e). Finally, we demonstrated that Tetra_*n*_ had little effect on BAM activity (Extended Data Fig. 7g), suggesting that cross-linked high-molecular weight forms of PG modulate BAM activity. It remains unclear why individual subunits of BAM vary in their ability to bind PG in vitro compared with the intact complex in vivo, which will require further mechanistic investigation. The variable nature of BAM binding to PG may have its origins in the large conformational changes undergone by the assembly during the OMP folding cycle^43^, which probably influences the positions of BAM components relative to the cell wall^37^ (Fig. 2i).

## PG modulates OMP patterning in vivo

**Extended Data Fig. 8:**
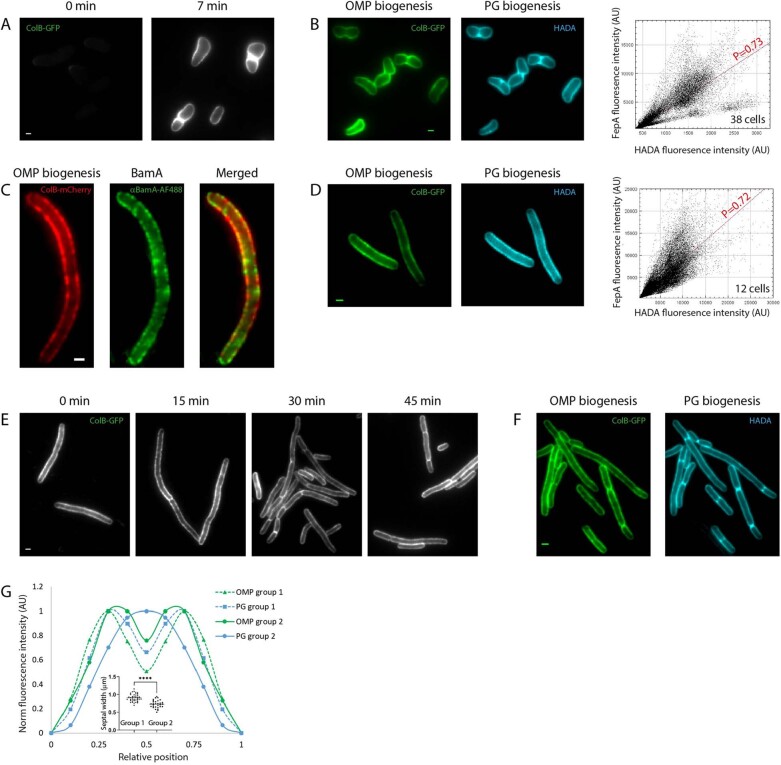
The effect of cell wall targeting antibiotics on OMP biogenesis. **(A)** Epifluorescence images of FepA in an inducible expression strain (GM07) treated with mecillinam. Shown are images of cells before and after induction with 0.4% arabinose. **(B)** Co-labelling of PG and OMP biogenesis in mecillinam treated cells. HADA and 0.4% arabinose were added simultaneously for 7 min. Shown are epifluoresence images (*left*) and pixel by pixel cytofluorogram of a single image (*right*). **(C)** Co-labelling of BamA and OMP biogenesis in aztreonam treated cells. Aztreonam was added 30 min before induction with 0.4% arabinose for an additional 7 min. **(D)** Co-labelling of PG and OMP biogenesis in aztreonam treated cells. HADA and 0.4% arabinose were added simultaneously for 7 min. Shown are epifluoresence images (*left*) and pixel by pixel cytofluorogram of a single image (*right*). **(E)** OMP biogenesis following the removal of aztreonam. Aztreonam was washed from the media at T = 0 min. OMP biogenesis was induced with 0.4% arabinose for 7 min prior to each time point. **(F)** Co-labelling of PG and OMP biogenesis 45 min after the removal of aztreonam from the media. HADA and 0.4% arabinose were added simultaneously for 7 min. **(G)** Comparison of the fluorescence intensity profiles of PG and OMP biogenesis across emerging septa as shown in panel F. Shown are profiles of cells at two different stages of septum formation (Group 1/Group 2). Inset displays the width of individual cells at the designated division plane. The chart demonstrates that PG biogenesis precedes the emergence of OMPs at division sites upon aztreonam removal. n_early_ = 50 cells, n_late_ = 30 cells. Statistical significance was calculated using two-tailed Student’s unpaired t-test (*P* = 0.0001). Scale bars, 1 μm.

Since mature and nascent PG have differential effects on the OMP folding activity of BAM in vitro, we hypothesized that cell wall composition is the driver of OMP patterning in the outer membrane of *E. coli* in vivo. We tested this hypothesis using two approaches: antibiotics that disrupt the PG and *E. coli* mutants with altered PG, in both cases monitoring PG incorporation using HADA labelling and OMP biogenesis using FepA labelling. Mecillinam, which inhibits the action of PBP2 within the elongasome causing cells to round up^44^, had a negligible effect on coordination between PG and OMP biogenesis as division sites were still formed (Extended Data Fig. 8a,b). Aztreonam, which inhibits PBP3-mediated PG cross-linking at the divisome^45^, inhibits formation of division septa. OMP biogenesis was observed across the filaments of aztreonam-treated cells except at the poles (Extended Data Fig. 8c,d and Supplementary Video 6), but this activity was lower than that at the septa of dividing cells, since removal of aztreonam allowed division sites to reform, accompanied by enhanced OMP biogenesis (Extended Data Fig. 8e–g).

**Fig. 4:**
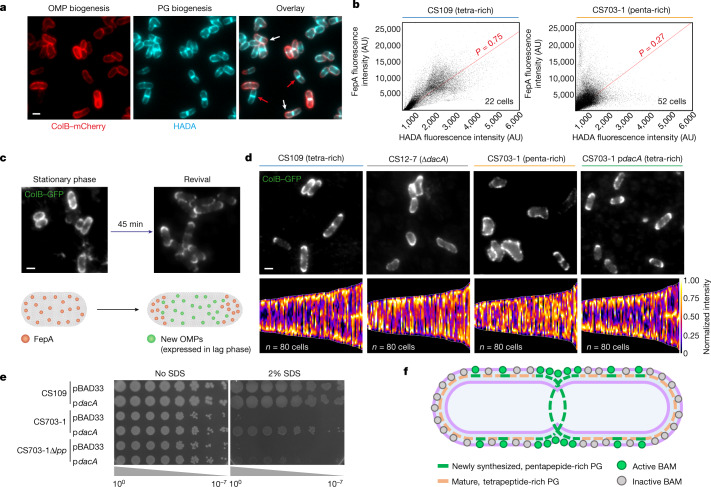
Cell wall composition affects the integrity and organization of the outer membrane. **a**, Co-labelling of PG and the OMP FepA in a pentapeptide-rich strain (CS703-1). PG labelling (HADA) and FepA induction (0.4% arabinose) were initiated simultaneously for a total period of 7 min. Arrows indicate cells showing disparity between OMP and PG biogenesis. **b**, The fluorescence intensity of FepA versus HADA in tetrapeptide-rich (left) and pentapeptide-rich (right) strains following co-labelling as in **a**. The representative pixel-by-pixel cytofluorograms of single images show that the strong correlation of OMP and PG biogenesis is abrogated in the pentapeptide-rich strain. **c**, Polar displacement assay whereby mid-cell OMP biogenesis bias can be discerned from the movement of stationary phase FepA as cells revive. Representative images before and after resuspension in fresh medium (top) and an illustration of OMP movement during this period (bottom). **d**, Polar displacement of stationary phase FepA during revival. Representative images (top) and demographs of normalized fluorescence intensities across multiple cells (bottom) 45 min after resuspension in fresh M9 medium. The images and demographs show how differently OMPs segregate between tetrapeptide-rich and pentapeptide-rich strains. **e**, Effect of PG remodelling by plasmid-produced PBP5 on SDS sensitivity in CS703-1 and CS703-1Δ*lpp*. Ectopic PBP5 production from p*dacA* partially restores outer membrane integrity. **f**, Model for spatial coupling of PG biosynthesis and BAM activity in the cell. BAM-mediated OMP insertion is dampened by mature PG, resulting in OMP biogenesis being largely absent at the old poles of cells and occurring predominantly at PG growth sites.

**Extended Data Fig. 9:**
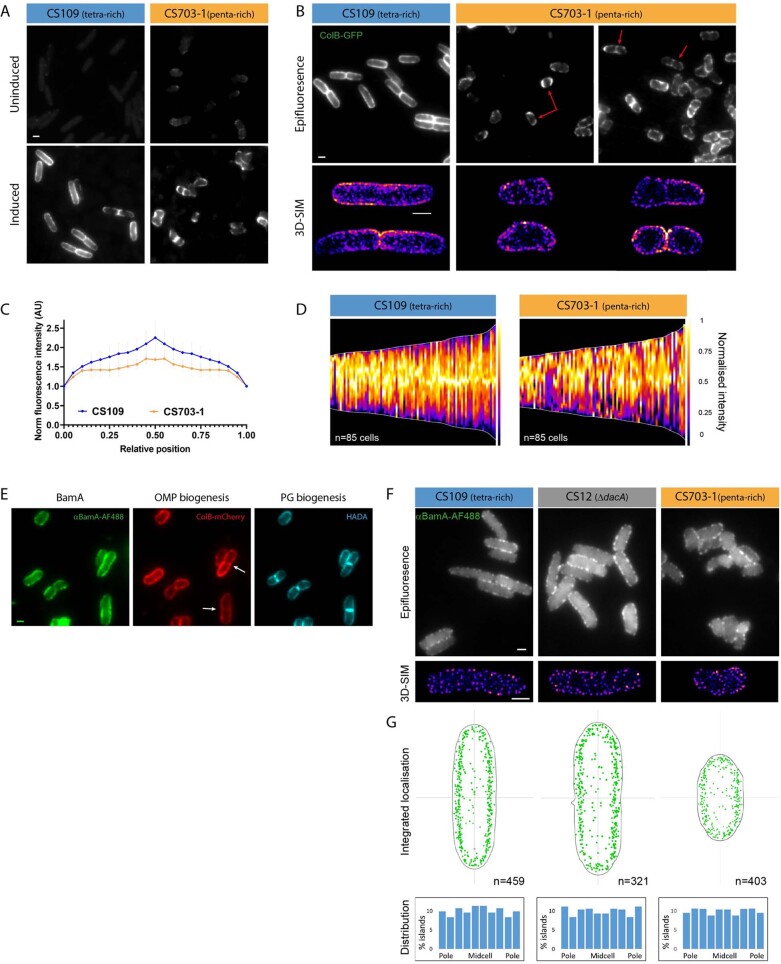
Increased levels of pentapeptide-rich PG diminishes midcell bias of OMP biogenesis. **(A)** Epifluorescence images of FepA, labelled with ColB-GFP in the indicated strains with or without induction of FepA expression. **(B)** Epifluorescence and 3D-SIM images of newly synthesised FepA in the indicated strains after 7 min induction (0.4% arabinose). Red arrows indicate cells exhibiting atypical biogenesis patterns compared to the CS109 parent strain. **(C)** Distribution of FepA localisation along normalised cell lengths in the strains from panel B (± SD, 3 biological replicates). **(D)** Normalised fluorescence intensity across the long-axis of multiple cells. Shown are demographs of FepA labelling 7 min after induction of the indicated strains. These charts and the images in panel B demonstrate the incoherent and reduced midcell bias for OMP biogenesis in the pentapeptide-rich strain. **(E)** Co-labelling of BamA, PG and OMP biogenesis in a penta-rich strain (CS703). HADA was added 3 min after FepA induction and the total induction time was 10 min. Arrows indicate septa where no increased OMP biogenesis was observed. **(F)** Epifluorescence (*top*) and 3D-SIM (*bottom*) images of the indicated strains labelled with αBamA^AF488^ Fabs. The images highlight that BamA organization is similar despite the changes in PG composition. **(G)** Comparison of BamA distribution in the strains from panel F. Shown are integrated localization maps (*top*) and charts showing the distribution of islands along the long axis of the cells (*bottom*). The charts show that BamA is uniformly distributed in all strains. Scale bars, 1 μm.

**Extended Data Fig. 10:**
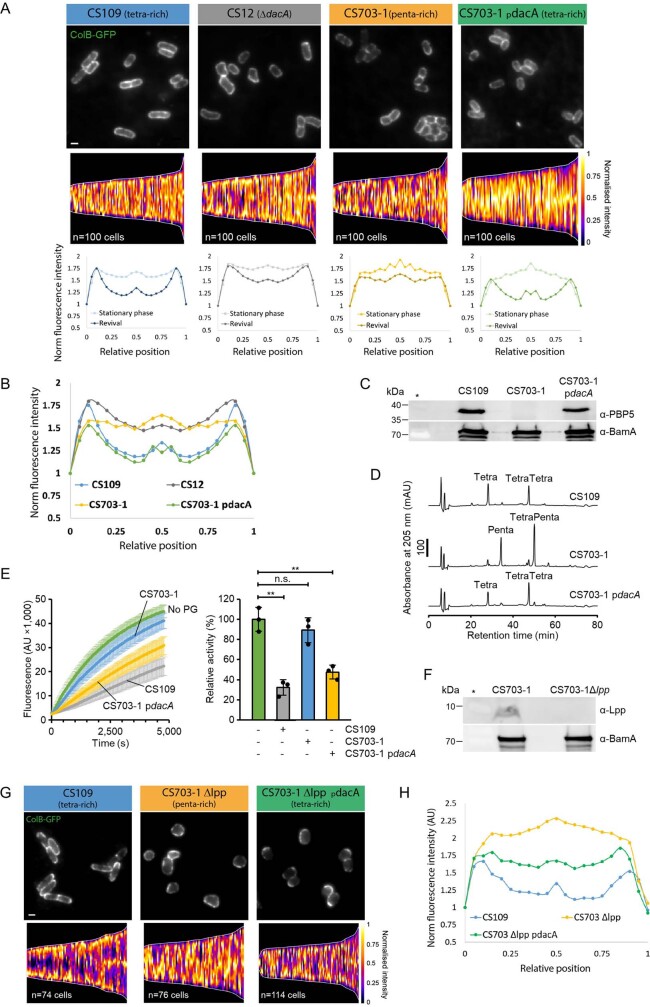
Complementation experiments by ectopic PBP5 production from p*dacA*. **(A)** Native FepA localisation before resuspension in fresh media (stationary phase). Shown are representative images of the indicated strains (*top*), demographs of the normalised fluorescence intensity across the long axis of multiple cells (*middle*) and distribution of native FepA before vs after revival (*bottom*). The charts and images demonstrate that FepA distribution is almost uniform at stationary phase in all strains but binary partitioning is diminished in penta-rich strains. **(B)** Distribution of FepA localisation along normalised cell lengths in the strains shown in Fig 4d. **(C)** Ectopic PBP5 production from p*dacA* in CS703-1 detected by Western Blot and specific antibodies (for blot source data, see SI Fig. 1). The marker lane is indicated by an asterisk. **(D)** HPLC analysis of cellosyl-digested PG sacculi shows that the CS703-1 cells expressing PBP5 restore a tetrapeptide-rich PG as is present in the CS109 strain. **(E)** Effect of PBP5-remodelled PG isolated from CS703-1 (with or without expression of PBP5) on BAM-mediated OmpT assembly *in vitro* (*n* = 3; control vs PG from CS109: *P* = 0.001; control vs PG from CS703-1: *P* = 0.34; control vs PG from CS703-1 p*dacA*: *P* = 0.003). Statistical significance was calculated using two-tailed Student’s unpaired t-test. Values are mean ± SD. **(F)** Western Blot analysis confirming the absence of Lpp in CS703-1Δ*lpp* (for blot source data, see SI Fig. 1). The marker lane is indicated by an asterisk. **(G)** Polar migration of pre-existing FepA during revival from stationary phase in the Δ*lpp* background. Shown are representative images (*top*) and demographs of the normalised fluorescence intensity across multiple cells (*bottom*) 45 min after resuspension in fresh M9 media. **(H)** Distribution of FepA localisation along normalised cell lengths in the strains from A. The charts demonstrate that *dacA* expression restores FepA polar migration in the pentapeptide-rich strain in Δ*lpp* background.

In the second approach, we induced OMP (FepA) expression in a pentapeptide-rich mutant strain (CS703-1) and its tetrapeptide-rich parent (CS109). In both strains, FepA appeared on the cell surface shortly after arabinose induction (Extended Data Fig. 9a). However, whereas the distribution of FepA in the tetrapeptide-rich strain was similar to that observed in a wild-type (BW25113) strain, including mid-cell bias, the pentapeptide-rich strain displayed a defective biogenesis pattern with reduced mid-cell bias (Extended Data Fig. 9b–d). Furthermore, we found that OMP and PG biogenesis no longer mirrored one another as co-localization was significantly reduced (Fig. 4a,b and Extended Data Fig. 9e). Given the aberrant nature of OMP insertion in this and other PG mutant strains, we developed a live-cell assay by which we could determine the effectiveness of mid-cell OMP insertion even in highly disrupted cell envelopes. This assay exploited the fact that several OMP genes in *E. coli*, including *fepA*, are highly expressed in late-exponential and stationary phase but are suppressed in lag-phase and early exponential cells^46,47^. We used the displacement of FepA, expressed from its endogenous promoter, as cells revived from stationary phase as a proxy for mid-cell OMP insertion (Fig. 4c). We used this assay to assess the effect of PG mutant strains on their ability to drive polar displacement of FepA after first having determined that all strains expressed similar levels of BamA (Extended Data Fig. 9f,g and 10c). We also determined that they displayed uniform (or near uniform) distribution of FepA in stationary phase (Extended Data Fig. 10a). Following revival, FepA distribution was bipolar in the wild type but nearly uniform in the pentapeptide-rich mutant, indicative of defective OMP insertion at mid-cell in the cell wall mutant (Fig. 4d and Extended Data Fig. 10b). Decreased polar displacement was also observed when only *dacA* (which encodes PBP5) was deleted (Fig. 4d and Extended Data Fig. 10b). This strain (CS12-7^39^) contains higher than normal levels of pentapeptide PG, but the increase is more moderate than in CS703-1^48^. Notably, restoration of FepA polar displacement was achieved upon plasmid expression of PBP5 in the CS703-1 background (Fig. 4d and Extended Data Fig. 10b–e). This effect was retained in a derivative of CS703-1 lacking *lpp* (CS703-1Δ*lpp*; Extended Data Fig. 10f–h), indicating that it is not the lack of outer membrane tethering to the PG that results in changes to OMP biogenesis patterns. Finally, we demonstrated that PBP5 expression in the CS703-1 background was sufficient to restore *E. coli* outer membrane stability when cells were challenged with the detergent sodium dodecyl sulfate (SDS) (Fig. 4e), demonstrating that the outer membrane instability associated with PG mutants is rescued when the cell wall defect is corrected. Cumulatively, our data show that it is the local density of pentapeptides that underpins the biogenesis patterns of OMPs and—consequently—the stability of the outer membrane in *E. coli*, since nascent pentapeptides do not dampen BAM OMP insertion activity as do matured tetrapeptides (Fig. 4f). Pentapeptides are absent in the PG of old poles but are abundant at division sites, whereas inhibitory tetrapeptides show the inverse distribution, explaining why little or no OMP biogenesis is seen in old polar regions of *E. coli*.

## Discussion

Our data show that although BamA clusters are uniformly distributed in the outer membrane, their capacity for catalysing OMP insertion is not uniform, but is modulated by the maturation state of the underlying PG, an effect that is probably enhanced by the low mobility of OMPs in the outer membrane^49^. Maximum OMP insertion activity occurs at division sites, owing to the inability of nascent PG to inhibit BAM. By contrast, little or no OMP insertion occurs at cell poles, owing to the shutdown of OMP folding and insertion activity by mature PG. Intermediate levels of OMP insertion are observed on the long axis of cells, reflecting the mixed maturation state of PG as cells elongate (Fig. 4f).

How different forms of PG affect BAM activity is not yet understood, but some elements of the crosstalk between these cell envelope layers can be inferred. Tetrapeptide-rich, non-cross-linked PG fragments bind to BAM proteins with micromolar affinity, but do not inhibit OMP foldase activity. Inhibition requires intact sacculi, suggesting that multiple sites within BAM have to be contacted simultaneously for inhibition to occur. This coordinate effect is reversed by the presence of a terminal d-alanine in nascent PG, blocking the ability of PG to associate with BAM. Tetrapeptide-rich PG could suppress BAM activity directly, for example by blocking access of unfolded OMP substrates to the lateral gate of BamA, or indirectly by restricting the conformational transitions of the BAM machinery that enable OMP folding and release into the outer membrane. Recent evidence points to the Sec translocon in the inner membrane, through which all OMPs are secreted, passing unfolded, chaperone-bound OMPs directly to BAM across the periplasm^50,51^. Thus, an additional element to PG-mediated control of BAM beyond that uncovered here could be the efficiency with which unfolded OMP substrates are transferred through the PG layer.

The modulation of OMP biogenesis by PG maturation has two important physiological consequences for the bacterium. First, it synchronizes insertion of OMPs with the growth of the PG. Such synchrony probably includes growth of the outer membrane itself, since the OMP LptD—which deposits lipopolysaccharides into the outer leaflet of the membrane—is a BamA substrate^52^. Second, biasing OMP insertion to septa or mid-cell regions enables the organism to turn over its OMPs simply by division (binary partitioning). As a result, the organism can rapidly alter its OMP composition in response to a changing environment without the need for active protein degradation^49^.

## Methods

### Strains, plasmids, oligonucleotides and antibodies used in this study

The bacterial strains used in this study are provided in Supplementary Table 1. A list of plasmids and oligonucleotides appear in Supplementary Tables 2 and 3, respectively. Antibodies and engineered bacteriocins are listed in Supplementary Table 4.

### Construction of pNGH206

For the construction of the pNGH206 plasmid, site-directed mutagenesis was used to introduce a solvent accessible cysteine (K469C) in the cytotoxic domain of a construct in which the N-terminal 62 amino acids of the colicin had been deleted (Δ2–61 ColE9).

### Construction of pBAD33-*dacA*

The *dacA* gene was amplified by PCR with Q5 Polymerase (NEB), using chromosomal DNA from *E. coli* BW25113 as template and the oligonucleotides dacA_F and dacA_R (Supplementary Table 3). Plasmid pBAD33 was amplified by PCR with Q5 Polymerase using oligonucleotides pBAD33_FR and pBAD33_RF (Supplementary Table 3). Insert and vector were joined by ligation-independent cloning^54^. Positive clones were selected on LB agar + 25 μg ml^−1^ chloramphenicol and identified by colony PCR using GoTaq G2 Polymerase (Promega). The correct sequence of the cloned *dacA* gene was confirmed by double-strand sequencing using specific oligonucleotides pBAD33_seq_F1, dacA_seq_R1, dacA_seq_F2 and pBAD33_seq_R2 (Supplementary Table 3). All oligonucleotides were obtained from Eurogentec.

### Expression and purification of antibodies and bacteriocins

Antibodies and engineered colicins used are listed in Supplementary Table 4. Anti-BamA MAB2 Fabs: a construct suitable for periplasmic expression of Fab in *E. coli* and containing a sequence coding for Fab fragments of MAB2 was cloned, transformed into 34B8 *E. coli* cells and expressed at 30 °C under control of the *phoA* promoter in CRAP phosphate-limiting autoinduction medium (PMID: 12009210) supplemented with carbenicillin (50 μg ml^−1^). After 24 h, cells were collected and resuspended in PBS supplemented with one complete EDTA-free Protease Inhibitor Cocktail tablet (Roche) per 50 ml of lysis buffer, lysozyme (0.125 mg ml^−1^), and benzonase (0.01 mg ml^−1^). The prepared suspension was microfluidized at 15,000 psi and clarified at 50,000*g* for 30 min at 4 °C. The supernatant was then resolved on protein G Sepharose beads equilibrated with PBS, using 2 ml packed resin volume per original gram of cell paste. The column was washed extensively with PBS and Fabs were eluted under mildly acidic conditions (0.56% glacial acetic acid pH 3.6). Eluted Fabs were immediately dialysed overnight at 4 °C against buffer containing 500 mM NaCl, 10% glycerol and 100 mM Tris (pH 8.0). Fabs were further purified on an S75 16/60 gel filtration column (GE Healthcare) using PBS (pH 7.2) as the running buffer. MAB2 Fab fragments were labelled by using Alexa Fluor 488 Protein Labeling Kit (ThermoFisher Scientific) following the manufacturer’s instructions. The fluorescently labelled Fabs were passed over HiPrep desalting column (GE Healthcare) to remove the excess dye. Peak fractions were collected and concentrated, and the degree of labelling was determined to be 1.42 dye molecules per Fab using liquid chromatography mass spectrometry (LC–MS). Colicin E9–AF488 expression and purification: the expression and purification of this protein has been previously described^8^. Here we used a modified construct with a single cysteine (Δ2–61 ColE9 K469C-Im9_His6_). Cys469 in the C-terminal DNase domain of these ColE9 constructs was labelled with a threefold excess of Alexa Fluor 488-maleimide (Invitrogen), as previously described^8^. The labelling efficiency (typically 0.8 fluorophores per protein) was estimated spectrophotometrically (V550 spectrophotometer, Jasco). Colicin B–GFP or colicin B–mCherry expression and purification: the expression and purification of these proteins has been previously described^27^. Pyocin S5–AF488 expression and purification: the expression and purification of this protein has previously been described^55^. Pyocin S2–mCherry expression and purification: the expression and purification of this protein has previously been described^56^. Here we fused mCherry in a similar manner. Primers and plasmids used for the expression of this construct appear in Supplementary Tables 2 and 3. CloDF13–AF488 expression and purification: DNA encoding receptor binding domain of cloacin DF13 with a cysteine at its C terminus was cloned C-terminal to the gene for the colicin E9 immunity protein, Im9, in the pQE-2 vector (Qiagen), to give pNGH382. BL21 (DE3) cells transformed with pNGH382 were grown at 37 °C to an optical density at 600 nm (OD_600_) of 0.8, upon which His6–Im9–CloDF13_301–460_Cys expression was induced through the addition of 1 mM IPTG. Cells were grown for a further 2 h at 37 °C, before being collected by centrifugation. Cells were resuspended in 20 mM Tris-HCl, pH 7.5, 8 mM imidazole, 0.5 M NaCl, 1 mM PMSF before being lysed by sonication. Cell lysate was clarified by centrifugation at 17,500*g* for 30 min at 4 °C, before passing the supernatant through a 0.45 µm filter and loading onto a 5 ml HisTrap HP column (Cytiva). Bound material was eluted from the column with a 4 to 500 mM imidazole gradient. Fractions containing His6–Im9–CloDF13_301–460_Cys were labelled with a 1.5-fold AF488-maleimide (Invitrogen) as previously described^8^. The labelling efficiency was estimated spectrophotometrically to be 98% (BioSpectrometer, Eppendorf).

### Expression and purification of soluble constructs of BamA P1,2 (residues 21–174), BamA P3,4 (residues 175–345), BamA P4,5 (266–422), BamB, BamC and BamE

Constructs for BamB and BamE were synthesised without their periplasmic export sequences with the cysteine at the beginning of the mature protein mutated to serine to remove their N-terminal acylation sites and cloned into pET22b(+) (Genscript, Novagen). A construct for BamC was synthesised lacking its periplasmic export sequence and N-terminal acylation site (residues 26–344) and incorporated into the pET16b expression vector with an N-terminal 6xHis-tag (Genscript, Novagen)^57^. A construct for BamA P1,2 (residues 21–174) was cloned into pQE70, with a C-terminal 4×His-tag^58^. Constructs for BamA P3,4 (residues 175–345) and BamA P4,5 (residues 266–422) were synthesised and cloned into pET26b(+), with a C-terminal 6×His-tag (Genscript, Novagen).

Cells containing the appropriate plasmid were grown in LB medium supplemented with 100 µg ml^−1^ ampicillin for BamB, BamC, BamE and BamA P1,2, and 30 µg ml^−1^ kanamycin for P3,4 and P4,5, to an OD_600_ of 0.4 and protein expression induced by the addition of 1 mM IPTG, at 18 °C overnight. Cultures were collected by centrifugation (6,000*g*, 15 min), resuspended in 50 mM sodium phosphate pH 7.5, 300 mM NaCl, 10 mM imidazole with EDTA-Free protease inhibitor tablets (Roche) and lysed using an Emulsiflex C3 cell disruptor (Avestin). The lysate was centrifuged at 75,000*g* for 45 min at 4 °C to pellet insoluble material. The supernatant was filtered through a 0.45 µM filter (Millipore), then purified via immobilized metal affinity chromatography using a 5 ml HisTrap HP column (GE Healthcare) in sodium phosphate buffer pH 7.5 followed by size-exclusion chromatography in 50 mM Sodium phosphate pH 7.5, 300 mM NaCl, using a Superdex 75 26/60 column (GE Healthcare). Fractions were assessed by SDS–PAGE, combined, concentrated using an Amicon Ultra 10 kDa MWCO centrifugal concentrator (Millipore) and stored at 4 °C for immediate use or frozen in liquid nitrogen and stored at −80 °C.

### Expression and purification of BamCD and BamCDE

Plasmids expressing BamCD (pSK46)^40^ and BamE with a C-terminal 6×His-tag (pBamE-His)^40^ were transformed separately into BL21(DE3) cells (New England Biolabs). Cells were grown in LB broth (supplemented with 50 µg ml^−1^ streptomycin for BamCD and 100 µg ml^−1^ ampicillin for BamE), at 37 °C, to an OD_600_ of 0.4 and expression induced by the addition of 0.5 mM IPTG, overnight, at 18 °C. Cells were collected separately by centrifugation (6,000*g*, 15 min), resuspended in 20 mM Tris pH 8.0, 150 mM NaCl with EDTA-Free protease inhibitor tablets (Roche) and lysed separately, using an Emulsiflex C3 cell disruptor (Avestin). The lysates were spun separately at 10,000*g* for 30 min at 4 °C and the supernatant centrifuged at 100,000*g* for 45 min to collect membranes. The membranes were solubilised (1 ml of buffer for every 40 mg of membrane) with 50 mM Tris pH 8.0, 150 mM NaCl and 1% *n*-dodecyl-β-d-maltoside (DDM) (Anatrace), combined and then rotated at 4 °C for 2 h. The solubilized membranes were centrifuged at 50,000*g* for 30 min; the supernatant was filtered through a 0.45 µm filter (Millipore), and then bound to equilibrated Ni-NTA agarose beads (Qiagen) overnight at 4 °C. The beads were washed with 3 column volumes of 50 mM Tris pH 8.0, 150 mM NaCl, 50 mM imidazole, 0.03% DDM and the protein was eluted with 2 column volumes of 50 mM Tris pH 8.0, 150 mM NaCl, 500 mM imidazole, 0.03% DDM. Fractions were assessed by SDS–PAGE and those containing BamCDE were pooled and further purified through size-exclusion chromatography using a Superdex 200 16/600 column (GE Healthcare) in 50 mM Tris pH 8.0, 150 mM NaCl and 0.03% DDM. Fractions were further assessed by SDS–PAGE, combined and stored at 4 °C for immediate use or frozen in liquid nitrogen and stored at −80 °C.

BamCD was co-purified from a modified version of plasmid pSK46 carrying a 6×His-tag at the C terminus of BamC following the same protocol, omitting the steps with plasmid pBamE-His.

### Purification of BamABCDE

The protocol was adapted from previous reports^41,42^. Plasmid pJH114^41^ was transformed into *E. coli* BL21(DE3). Cells were grown in LB (10 g l^−1^ NaCl) containing 100 µg ml^−1^ ampicillin up to OD_600_ of 0.5–0.6, and expression induced with 0.4 mM IPTG by incubating at 37 °C for 90 min with orbital shaking (175 rpm). Cells were collected (6,200*g*, 4 °C, 15 min), resuspended in buffer A (20 mM Tris/HCl, pH 8.0) and disrupted by sonication. Membranes were collected by ultracentrifugation (130,000*g*, 4 °C, 1 h) and solubilised in buffer B containing 50 mM Tris/HCl, 150 mM NaCl, 1% DDM (Avanti) at pH 8.0 by incubating for 1 h on ice. The sample was incubated with 2 ml per l of culture volume of Ni-NTA agarose beads (Qiagen) and rotated overnight at 4 °C on a tube roller. Beads were washed in buffer C (50 mM Tris/HCl, 150 mM NaCl, 50 mM imidazole, 0.05% DDM, pH 8.0) and proteins eluted in buffer D (50 mM Tris/HCl, 150 mM NaCl, 500 mM imidazole, 0.05% DDM, pH 8.0). Eluted fractions were concentrated to ~500 µl in ultrafiltration units and applied to a Superdex 200 (10/300) column (GE Healthcare), in filtered and degassed buffer E (50 mM Tris/HCl, 150 mM NaCl, 0.05% DDM, pH 8.0) at 0.5 ml min^−1^, collecting 500 µl fractions. Protein purity and yield was analysed by SDS–PAGE. Fractions containing BamABCDE were combined and immediately reconstituted into proteoliposomes or snap-frozen in liquid nitrogen and stored in small aliquots at −80 °C.

### Purification of SurA

The protocol was adapted from previous reports^40,41^. SurA was overproduced in *E. coli* BL21(DE3) by growing cells in LB (10 g l^−1^ NaCl) containing 50 µg ml^−1^ kanamycin up to an OD_600_ of ~1.0. The temperature was shifted to 16 °C and 0.1 mM (final concentration) IPTG was added and the cells incubated at 16 °C for ~16–18 h. Cells were collected (6,200*g*, 4 °C, 15 min), resuspended in buffer A (20 mM Tris/HCl, pH 8.0) and disrupted by sonication. The soluble fraction was incubated with 2 ml per l of culture volume of Ni-NTA agarose beads (Qiagen) and rotated overnight at 4 °C on a tube roller. Beads were washed in buffer B (20 mM Tris/HCl, 50 mM imidazole, pH 8.0) and the protein was eluted in buffer C (20 mM Tris/HCl, 500 mM imidazole, pH 8.0). Eluted fractions were dialysed against buffer D (20 mM Tris/HCl, 10% glycerol) overnight at 4 °C, then concentrated to ~5 ml and applied to a Superdex 75 (16/600) column (GE Healthcare), in filtered and degassed buffer D at 1 ml min^−1^. Eluted fractions were analysed by SDS–PAGE to assess protein purity and yield. Fractions containing SurA were combined and concentrated to 250–300 µM in a Vivaspin Turbo 10 kDa centrifugal concentrator (Sartorius), and stored in aliquots at −80 °C.

### Purification of OmpT

An adapted protocol was used^42^. OmpT was overproduced as cytoplasmic inclusion bodies in *E. coli* BL21(DE3) by growing cells in LB (10 g l^−1^ NaCl) containing 50 µg ml^−1^ kanamycin up to OD_600_ ~0.5-0.6, adding 1 mM IPTG and incubating for 4 h at 37 °C. Cells were collected (6,200*g*, 4 °C, 15 min), resuspended in buffer A (50 mM Tris/HCl, 5 mM EDTA, pH 8.0) and disrupted by sonication. The insoluble fraction was collected by centrifugation (4,500*g*, 4 °C, 15 min) and resuspended in buffer B (50 mM Tris/HCl, 2% Triton X-100, pH 8.0), then incubated for 1 h at room temperature with gentle shaking. Inclusion bodies were pelleted (4,500*g*, 4 °C, 15 min) and washed twice in buffer C (50 mM Tris/HCl, pH 8.0) by incubating for 1 h at room temperature, then solubilized in buffer D (25 mM Tris/HCl, 6 M guanidine-HCl, pH 8.0). The supernatant was filtered, concentrated to ~5 ml in a Vivaspin Turbo 10 kDa centrifugal concentrator (Sartorius), and applied to a Superdex 75 (26/600) column (GE Healthcare) with filtered and degassed buffer D at 1 ml min^−1^. Eluted fractions were analysed by SDS–PAGE to assess protein purity and yield. Fractions containing OmpT were combined and stored in aliquots at −80 °C.

### Purification of MepM

An adapted protocol was used^33^. Soluble MepM carrying a C-terminal 6×His-Tag was overproduced in the cytoplasm of *E. coli* BL21 (DE3) from plasmid pMN86. Cells were grown in LB (10 g l^−1^ NaCl) containing 100 µg ml^−1^ ampicillin at 37 °C with shaking up to OD_600_ ~0.6. The culture was shifted to 25 °C and supplemented with 50 μM IPTG after 30 min to induce protein overproduction. Induction was followed for 2 h. Cells were collected by centrifugation (6,200*g*, 4 °C, 15 min) and resuspended in buffer A (25 mM Tris/HCl, 300 mM NaCl, 10 mM MgCl_2_, 20 mM imidazole, 10% glycerol, pH 7.0). Cells were disrupted by sonication and the soluble fraction applied to a 5 ml HisTrap HP column at 1 ml min^−1^. The column was washed with 5 column volumes of buffer A at 1 ml min^−1^. MepM was eluted at 1 ml min^−1^ in buffer B (25 mM Tris/HCl, 300 mM NaCl, 10 mM MgCl_2_, 400 mM imidazole, 10% glycerol, pH 7.0). Fractions containing MepM were pooled and dialysed overnight at 4 °C against buffer C (25 mM Tris/HCl, 300 mM NaCl, 10 mM MgCl_2_, 10% glycerol, pH 7.0), then concentrated to a volume of ~5 ml and applied to a Superdex 75 (16/600) column (GE Healthcare), in filtered and degassed buffer C at 1 ml min^−1^. Eluted fractions were analysed by SDS–PAGE and fractions containing MepM were combined and stored in aliquots at −80 °C.

### Isolation of PG sacculi

An adapted protocol was used^59^. Cells were grown in 4 l of LB (10 g l^−1^ NaCl) at 37 °C with orbital shaking (175 rpm), up to OD_600_ ~0.5–0.6. Cultures were incubated on ice for 10 min to stop cell growth. Cells were collected (6,200*g*, 4 °C, 15 min) and resuspended in 40 ml of ice-cold Milli-Q water. The cell suspension was added drop-wise to 40 ml of boiling 8% SDS and boiled with vigorous stirring for 30 min. After cooling down to room temperature, sacculi were collected by ultracentrifugation (130,000*g*, 25 °C, 1 h) and washed in Milli-Q water. Ultracentrifugation and washing steps were repeated until samples were SDS-free^59^. Sacculi were resuspended in 9 ml of 10 mM Tris/HCl, 10 mM NaCl, pH 7.0, supplemented with 1.5 mg of α-amylase (Sigma-Aldrich) and incubated at 37 °C for 2 h. Samples were supplemented with 2 mg of Pronase E (Sigma-Aldrich) and incubated at 60 °C for 1 h. Reactions were stopped by adding 4% SDS (1:1 v/v) and boiling at 100 °C for 15 min, then samples washed until SDS-free as before. Purified sacculi were resuspended at ~10 mg ml^−1^ in 0.02% NaN_3_ and stored at 4 °C. PG preparations were quantified by digestion with the muramidase cellosyl followed by the reduction of the resulting muropeptides by sodium borohydride and their separation by HPLC using detection at 205 nm. The total area of the muropeptides was compared with that of a standard sample with a known concentration that was estimated from UV absorbance, as described^59^. When comparing tetrapeptide- and pentapeptide-rich PG in pull-down assays and BAM activity assays, the amounts of the different PG preparations used was adjusted to the same UV absorbance of muropeptides released from the PG.

### Preparation of disaccharide-tetrapeptide chains for MST experiments

Isolated sacculi from *E. coli* BW25113Δ6LDT^30^ (~5.6 mg ml^−1^) were incubated with MepM (3 µM) in 25 mM Tris/HCl, 150 mM NaCl, 0.05% Triton X-100, pH 7.5. Negative control samples containing no sacculi (mock digests) for MST experiments were prepared in parallel. Samples were incubated for ~18 h at 37 °C with shaking and boiled at 100 °C for 10 min. The released soluble PG fragments (Tetra_*n*_) were collected by centrifugation (17,000*g*, room temperature, 15 min) and dialysed against 50 mM sodium phosphate, 150 mM NaCl, pH 7.0 for ~24 h at room temperature in a 3.5 kDa dialysis membrane. Dialysed Tetra_*n*_ was stored at −20 °C.

### HPLC analysis of PG and Tetra_*n*_

For composition analysis and quantification, purified PG sacculi or Tetra_*n*_ (~100 µg) were digested with cellosyl (0.5 μg ml^−1^) for 16–18 h at 37 °C in 20 mM sodium phosphate, pH 4.8 with shaking (1,000 rpm). Digestions were stopped by boiling at 100 °C for 10 min. Muropeptides were collected after centrifugation (15,000*g*, 15 min), reduced with sodium borohydride as described^59^ and analysed by reversed-phase HPLC at 55 °C in a 90- or 180-min linear gradient from 50 mM sodium phosphate, pH 4.31 to 75 mM sodium phosphate, pH 4.95, 15% methanol, on an Agilent 1220 Infinity HPLC system (Agilent). Relative concentrations of muropeptides from different sacculi preparations were estimated by comparing the total peak area from the integration of the UV signal from HPLC chromatograms^59^.

### PG pull-down assay

The protocol was adapted from previous reports^31,32^. PG sacculi (~1 mg) were incubated with purified protein (5 µM) in PG binding buffer (50 mM Tris/maleate, 50 mM NaCl, 10 mM MgCl_2_, pH 7.5) in a total volume of 100 µl. Samples were incubated on ice for 30 min, then pelleted by centrifugation (17,000*g*, room temperature, 10 min) and the supernatant was collected (supernatant fraction, S). PG pellets were washed by resuspending in 200 µl of PG binding buffer and pelleting again (17,000*g*, room temperature, 10 min) and the supernatant was recovered (wash fraction, W). PG-bound protein was released from sacculi by resuspending the PG pellet in 100 µl of 2% SDS and stirring for 1 h at room temperature. Samples were centrifuged (17,000*g*, room temperature, 10 min) and the supernatant was collected (pellet fraction, P). Fractions S, W and P were analysed by SDS–PAGE on 15% polyacrylamide gels and proteins visualised by Coomassie Blue staining. Protein retention in fraction P indicated binding of the protein to PG sacculi. For experiments performed with the full-length BamABCDE complex, PG binding buffer was supplemented with 0.05% Triton X-100. PG pull-down experiments with SurA were performed in 20 mM Tris/HCl, pH 6.5.

### Microscale thermophoresis

Purified proteins were labelled with Red–NHS (NanoTemper Technologies) in MST labelling buffer (50 mM sodium phosphate, 150 mM NaCl, 10% glycerol, pH 7.0) according to the manufacturer’s protocol. The concentration of fluorescently labelled proteins and efficiency of labelling was determined spectrophotometrically. MST experiments were performed as follows: serial dilutions of Tetra_*n*_ (from ~5.6 mg ml^−1^ to ~0.2 μg ml^−1^, 16 samples in total) or mock digestions were prepared in MST buffer in a total volume of 10 μl, mixed to an equal volume of labelled protein at 100 nM in MST buffer supplemented with 0.1% reduced Triton X-100 (Sigma-Aldrich) in order to obtain a serial dilution of ligand from ~2.8 mg ml^−1^ to ~0.1 μg ml^−1^ (~90 μM to ~3 nM, estimating an average molecular weight of 30 kDa for Tetra_*n*_), a final protein concentration of 50 nM, detergent concentration of 0.05% and reaction volume of 20 μl. Samples were incubated for 5 min on ice and 5 min at room temperature and loaded into standard-coated MST capillaries. Measurements were performed in a Monolith NT.115 (NanoTemper Technologies). LED power for each set of experiments was chosen in order to obtain initial fluorescence values between 200 and 2,500 counts for each individual protein during the capillary scan. Thermophoresis was analysed at the steady-state region of each thermogram. Curve fitting for *K*_d_ measurements was performed by plotting the normalized fluorescence intensity (*F*_norm_) at the steady state for each sample against ligand concentration according to a 1:1 binding model, as an average of three independent experiments. Results were analysed using the MO-Affinity Analysis software (NanoTemper Technologies).

Alternatively, for proteins that exhibited variations in initial fluorescence greater than ±10% of the average fluorescence along the serial dilution prior to the application of the temperature gradient, curve fitting was performed by directly plotting the initial fluorescence of samples against ligand concentration. *K*_d_ was calculated assuming a 1:1 binding model from an average of three independent experiments. To confirm that initial fluorescence changes were ligand-dependent, SDS-denaturation tests were performed as follows: after preparing the serial dilution as for the main interaction experiments, the initial fluorescence of three capillary samples representative of the bound fraction (capillary 1, 2 and 3) and three capillary samples representative of the unbound fraction (capillary 14, 15 and 16) was first measured to determine the differences between bound and unbound states. Samples were then centrifuged at 15,000*g* for 10 min, and the supernatant mixed 1:1 with 2× SD-mix (40 mM DTT, 4% SDS) and boiled at 95 °C for 10 min. The initial fluorescence for the chosen samples was then measured again and compared to the initial fluorescence observed before the SDS-denaturation tests. The initial difference was confirmed to be ligand-dependent if initial differences in fluorescence between samples from the bound and unbound fractions disappeared by the SDS-treatment. SDS-denaturation tests (SDS-tests) were performed in triplicate and analysed using the MO-Affinity Analysis software (NanoTemper Technologies).

### Reconstitution of BamABCDE into liposomes

The protocol was adapted from previous reports^40,41,60^. *E. coli* polar lipids (Avanti) were resuspended at 20 mg ml^−1^ in water, well dispersed by sonication and 200 µl were mixed with 1 ml of freshly purified BAM complex, and incubated on ice for 5 min. The mixture was diluted with 20 ml of 20 mM Tris/HCl, pH 8.0 and incubated on ice for 30 min. Proteoliposomes were pelleted by ultracentrifugation (135,000*g*, 4 °C, 30 min) and washed in 20 ml of 20 mM Tris/HCl pH 8.0, pelleted again and resuspended in 800 μl of 20 mM Tris/HCl pH 8.0. Efficiency of reconstitution was assessed by SDS–PAGE, analysing the supernatant, wash and pellet fractions. Proteoliposomes prepared by this method contain BAM complexes that are almost exclusively oriented outwards (that is, the periplasmic part of the complex is exposed on the liposome surface^60^). Aliquots (20 μl) of proteoliposomes were snap-frozen in liquid nitrogen and stored at −80 °C.

### In vitro BAM activity assay

Two 25 µl sub-reactions (A and B) were assembled in 20 mM Tris-HCl, pH 6.5 as follows: sub-reaction A contained SurA (140 µM) and OmpT (20 µM); sub-reaction B contained BAM proteoliposomes (2 µM), fluorogenic peptide (Peptide Synthetics) (2 mM). The two sub-reactions were assembled in half-area, black microplates (Corning) and incubated at 30 °C for 5 min, and mixed to start OmpT folding (final concentrations: 1 µM BAM proteoliposomes, 1 mM fluorogenic peptide, 70 µM SurA and 10 µM OmpT in a total volume of 50 µl). When required, PG sacculi or Tetra_*n*_ prepared in 20 mM Tris-HCl, pH 6.5 were supplemented to sub-reaction B. Fluorescence emission (excitation at 330 nm, emission at 430 nm) upon cleavage of the fluorogenic peptide by folded OmpT was monitored at 30 °C for 1 h 20 min after in a FLUOStar Microplate Reader (BMG Labtech), with readings every 20 s and 5 s orbital shaking prior to each reading. Three independent replicates were analysed for each experiment. Activity rates for each replicate were analysed over the linear range in the fluorescence release curve, averaged and converted into percentage relative to control reactions containing no PG. EC_50_ values for tetrapeptide-rich and pentapeptide-rich PG were estimated using the online MyCurveFit tool (https://mycurvefit.com/).

### In vitro BAM activity assay with pre-folded OmpT

For experiments in which OmpT was folded prior to fluorescence measurements, reactions were assembled as described and incubated for 2 h 30 min at 30 °C with orbital shaking to allow BAM-mediated OmpT assembly. Samples were then mixed with 50 µl reactions containing either 2 mM fluorogenic peptide or 2 mM fluorogenic peptide and 5 mg/ml sacculi from *E. coli* MC1061 in 20 mM Tris-HCl, pH 6.5, and OmpT activity was monitored as described.

### In vitro BAM activity assay with an excess of SurA

Experiments containing an excess of SurA were performed as follows. SurA (15 µM) was mixed with 1 mg of sacculi from *E. coli* MC1061 in 20 mM Tris-HCl, pH 6.5, in a total volume of 200 µl. Control samples contained no PG. Samples were incubated on ice for 30 min, then split in half. One half was added to OmpT folding reactions, assembled as described (BAM activity control reactions containing no PG and no extra SurA, or PG only, were included). The other half was used to monitor PG binding of SurA as described. OmpT activity was measured as above.

### Statistical analysis of in vitro BAM activity experiments

Statistical significance was calculated using two-tailed Student’s unpaired *t*-test. Differences were considered statistically significant for *P* < 0.05. Statistical significance was indicated as follows: NS, *P* > 0.05 (not significant); *P < 0.05; **P ≤ 0.01; ***P ≤ 0.001. Exact *P* values are indicated in the figure legends.

### Western blot analysis

*E. coli* cell suspensions were mixed 1:1 with 2× SDS–PAGE loading buffer (200 mM Tris-HCl, pH 6.8, 4% SDS, 0.2% bromophenol blue, 20% glycerol, 10% β-mercaptoethanol) and boiled at 100 °C for 10 min. Samples were loaded on 15% polyacrylamide gels and proteins resolved by SDS–PAGE, then transferred to nitrocellulose membranes and probed with specific primary antibodies (anti-BamA 1:40,000; anti-BamB 1:3,000; anti-BamC 1:20,000; anti-BamE 1:1,500; anti-Pal 1:2,500; anti-CpoB 1:2,500; anti-PBP5 1:1,000; anti-Lpp 1:3,000). Goat anti-rabbit HRP-IgG (Sigma–Aldrich, 1:5,000) was used as secondary antibody. Western Blots were developed using ECL Prime Western Blotting System (GE Healthcare).

### In vivo cross-linking of Bam proteins to PG

The protocol was adapted from a previous report^34^. In brief, *E. coli* MC1061 was grown in 50 ml of LB (5 g l^−1^ NaCl) at 37 °C by orbital shaking up to OD_600_ ~0.5. Cells were pelleted (4,500*g*, room temperature, 10 min), the cell pellet washed with 50 ml of phosphate-buffered saline (PBS) three times and the OD_600_ adjusted to 2.0 with PBS. 3,3′-dithiobis (sulfosuccinimidyl propionate) (DTSSP, ThermoFisher) was freshly dissolved in 5 mM sodium citrate, pH 5.0 and added to cells to a final concentration of 0.5 mM. Cells were incubated at room temperature for 10 min. The cross-linking reaction was quenched by adding Tris/HCl, pH 8.0 to a final concentration of 50 mM, incubating at room temperature for 15 min. Whole cell samples were taken for Western Blot analysis by concentrating 300 µl of cells 3-fold and mixing 1:1 v/v with 2× SDS–PAGE buffer (200 mM Tris/HCl pH 6.8, 4% SDS, 20% glycerol, 0.2% bromophenol blue) with or without 10% β-mercaptoethanol. The rest of the bacterial suspension was added drop-wise to an equal volume of boiling 8% SDS and boiled with vigorous stirring for 30 min to isolate PG sacculi. After cooling down to room temperature, sacculi were pelleted by ultracentrifugation (130,000*g*, room temperature, 1 h) and washed twice in 2% SDS, then resuspended in ~100 μl of 2% SDS. To analyse PG-bound proteins, sacculi suspensions were boiled in SDS–PAGE buffer with or without 10% β-mercaptoethanol at 100 °C for 10 min, briefly centrifuged, and supernatants loaded on 15% polyacrylamide gels, together with whole cell samples taken after cross-linking. Proteins were transferred to nitrocellulose membranes for Western blot analysis.

### SDS sensitivity assay

*E. coli* strains were grown from a single colony in LB + 25 µg ml^−1^ chloramphenicol at 37 °C by orbital shaking for ~16–18 h. The OD_600_ was adjusted to 2.0 and cells were serially diluted to 10^−7^ in growth medium, then plated with a pin replicator on LB agar + 25 µg ml^−1^ chloramphenicol + 0.2% arabinose, with or without 2% SDS. Plates were incubated at 37 °C and photographed after 24 h of incubation.

### Cell preparation for live microscopy

Overnight LB (10 g l^−1^ tryptone, 10 g l^−1^ NaCl, 5 g l^−1^ yeast extract (pH 7.2)), supplemented M9-glucose medium (0.4% (w/v) d-glucose, 2 mM MgSO_4_, 0.1 mM CaCl_2_, 1 mg ml^−1^ NH_4_Cl, 0.05% (w/v) casamino acids) cultures were grown at 37 °C and diluted 1:100 into fresh medium with appropriate antibiotics. Cultures were grown at 37 °C, unless stated otherwise, to mid-log phase (OD_600_ = 0.2–0.7) and cells were centrifuged at 7,000*g* for 1 min. For translation inhibition experiments, cells were treated with chloramphenicol (30 µg ml^−1^) 30 min before samples were taken. Agar pads were prepared by mixing supplemented M9-glucose medium or PBS with 1% agarose and pouring 150 µl into 1.5 × 1.6 gene frame (Thermo Scientific AB0577) attached to the slide. For pad formation, the gene frame was sealed by a coverslip until agarose solidified. Six microliters of cells were pipetted onto the agar pad, allowed to dry and sealed with a clean coverslip. For the induction of OMPs from a plasmid, 0.4% (w/v) arabinose was added directly into the growing culture 7 min before samples were taken, unless stated otherwise.

For live-cell labelling, an equivalent of 1ml of cells at OD_600_ = 0.25 were pelleted by centrifugation (7,000*g*, 1 min) and the samples were resuspended in supplemented M9-glucose medium containing 200 nM fluorescently labelled MAB2. Labelling was carried out for 20 min at room temperature with mixing by rotary inversion in an opaque tube. Subsequently the cells were washed twice (M9-glucose) by pelleting (7,000*g*, 1 min) and finally resuspended in ~50 μl M9-glucose. For fixed cell labelling, cells were pelleted by centrifugation (7,000*g*, 1 min) and the samples were resuspended in 4% formaldehyde (in PBS) at 4 °C immediately after sampling. After 20 min, cells were pelleted by centrifugation (7,000*g*, 1 min) and resuspended in 100 μl of fresh supplemented PBS containing 200 nM fluorescently labelled Fabs or colicins. Labelling was carried out as with live cells. After labelling, cells were washed 3 times (in PBS) by pelleting (7,000*g*, 1 min) and finally resuspended in ~50 μl PBS. To improve binding of the MAB2 Fabs in co-labelling experiments, after the labelling step cells were washed once (in PBS), pelleted (7,000*g*, 1 min) and resuspended in 4% formaldehyde (in PBS) at 4 °C for further 20 min. Cells were then washed twice (PBS) and resuspended in ~50 μl PBS.

The fluorescent d-amino acid HADA (Tocris Bioscience) was used for cell wall labelling. The labelling was carried as described previously^61^ with minor adjustments. The final concentration of HADA in the growing culture was 500 µM and the incubation time varied according to the aims of the experiment. When both PG and OMP labelling were included, the HADA labelling protocol was used first. After completing the last step of HADA labelling (fixation), samples were labelled using the relevant colicins as described above. For *P. aeruginosa* polar displacement experiments, HADA was added to the overnight culture and washed before resuspension in fresh medium.

### Induction and suppression of chromosomal OMP expression

For *E. coli*, chromosomal expression of FepA was induced by the addition of 200 µM 2,2 Bipyridyl (Sigma-Aldrich) to LB medium during mid-log phase. For, *K. pneumoniae*, chromosomal expression of IutA was induced by growing U11 cells in LB overnight (stationary phase) and transferring them into fresh M9. For *P. aeruginosa*, suppression of FpvAI and FptA expression for polar displacement experiments was carried out by growing PAO1 cells in M9 overnight (stationary phase) and transferring them into fresh LB medium containing 200 µM FeCl_3_.

### Total internal reflection fluorescence microscopy acquisition

Live cells were imaged using an Oxford NanoImager (ONI) super-resolution microscope equipped with four laser lines (405, 473, 561 and 640 nm) and ×100 oil-immersion objective (Olympus 1.49 NA). Fluorescence images were acquired by scanning a 50 µm × 80 µm area with a 473 nm laser for AF488- and GFP-labelled proteins (laser power 1.4–2.3 mW) or 561 nm for mCherry-labelled proteins (laser power 2.1–3.4 mW). The laser was set at 50° incidence angle (200 ms exposition), resulting in a 512 × 1,024 pixel image. Images were recorded by NimOS software associated with the ONI instrument. Each image was acquired as a 20-frame stack for brightfield and fluorescence channels, respectively. For analysis, images were stacked into composite images using average intensity as a projection type in ImageJ (version 1.52p). To ensure non-uniform fluorescence of labelled OMPs was not the result of proximity to the coverslip, equivalent images were taken in epifluorescence and by 3D-SIM microscopy where such potential bias was absent.

### Epifluorescence and SIM imaging

Cells were imaged using Deltavision OMX V3 Blaze microscopy system (GE Healthcare) equipped with four laser lines (405, 488, 593 and 633 nm), pco.edge 5.5 sCMOS cameras (PCO), a standard or a green/red drawer filter set and a ×60 oil-immersion objective (Olympus 1.42 NA). Three-dimensional-SIM three-colour images were taken using Deltavision OMX-SR microscopy system (GE Healthcare) equipped with four laser lines (405, 488, 568 and 640 nm), pco.edge 4.4 sCMOS cameras (PCO) and a ×60 oil-immersion objective (Olympus PlanApo 1.42 NA). For both conventional and SIM imaging 1.512 index refraction immersion oil was used for AF488- and GFP-labelled proteins and for mCherry/GFP/HADA three-colour imaging. For mCherry-labelled proteins or AF488–mCherry dual-colour imaging 1.514 index refraction immersion oil was used. Conventional fluorescence images were acquired by imaging a 42 μm × 42 μm area with the 488 nm laser (5.7 mW, 500 ms exposure) resulting in a 512 × 512 pixel image. For SIM acquisition, a similar area was imaged using the 488 nm laser (2.7 mW, 200 ms exposure). Image stacks of 1–1.5 μm thickness were taken with 0.125 μm *z*-steps and 15 images (three angles and five phases per angle) per *z*-section and a 3D structured illumination with stripe separation of 213 nm and 238 nm at 488 nm and 594 nm, respectively. The SIMcheck plugin (ImageJ) was used to assess the data quality of SIM images. Image stacks were reconstructed using Deltavision softWoRx 7.2.0 software with a Wiener filter of 0.003 using wavelength specific experimentally determined OTF functions. Average intensity and 3D projections of 3D-SIM images were generated using ImageJ (V1.52p).

For the acquisition of multi-channel images, a DIC image was taken first followed by an imaging sequence which minimized any possible overlap between channels. Fluorophores with higher excitation–emission spectra were imaged first and the fluorescent signal was bleached prior to the acquisition of the following channel. Alignment of dual-colour images was carried out using TetraSpeck Microspheres, 0.1 µm (ThermoFisher scientific) and the channel aligner tool (ImageJ V1.52p).

### Image and data analysis

Two-dimensional SIM images of BamA labelled cells were binarized and regions of interest (ROIs) were generated. Non-distinct islands were manually excluded. The size of each island was calculated based on its Ferret’s diameter (ImageJ V1.52p). For measurement of septal cell widths, the DIC and epifluorescence images were overlaid and the HADA channel used to determine the location of the developing septum along the long axis of the cell. Measuring the width of the cell at the selected region was done using the DIC channel (ImageJ V1.52p).

For the detection of islands and creating integrated localization maps an Unsharp mask filter (Radius 2 px, Mask weight 0.5) was applied to the raw images and BamA islands identified using the Find Maxima process (Prominence > 600) (ImageJ) and ROI were created. The fluorescence intensity of each ROI was measured using the raw images and background fluorescence was subtracted. Integrated localization maps of BamA islands were created using the ImageJ plugin MicrobeJ (v5.13m)^62^. For the detection of BamA islands with MicrobeJ, Unsharp Mask filter was applied, as described above. Cells and maxima points detection was carried out using the MicrobeJ plugin, In some cases the auto segmentation was manually corrected to exclude improperly detected or clustered cells.

Measurement of individual fluorescent intensity profiles and calculation of the normalized fluorescence distribution of OMPs were carried out as follows: For the early experiments (Fig. 1, and the related supplementary figures) fluorescence distribution across the long axis of the outer membrane was determined by the plot profile function (ImageJ V1.52). After measuring the raw values, they were normalized to 0−1 scale for comparison between cells. To enable the integration of fluorescence intensity distribution from cells of different lengths, the long axis of each cell was normalized to 0−100. In later experiments (Figs. 2–4 and the related extended data figures), the measurement of the fluorescence intensity profile and normalization of the long axis between 0–100 was automated using the MicrobeJ plugin (v5.13m). After the integration of profiles from all cells, the value at the poles was set to 1 and the remaining values normalized accordingly. Normalization was done using Excel and the data was plotted in GraphPad Prism 8 software.

Demographs presenting the fluorescence distribution of OMPs in a large population of cells were created using the MicrobeJ plugin (v5.13m). Each demograph included cells from at least three different fields of view. For all experiments, cells were sorted by length in order to highlight the different cell cycle stages. For induced OMP biogenesis experiments the pole with the higher fluorescence intensity was aligned to the top in order to highlight the unipolar distribution pattern.

For co-localization measurements the two compared channels were overlaid (following channel alignment) using the ‘merge channels’ tool (ImageJ V1.52) to confirm that cells are properly aligned. Next, the fluorescence image from the 405nm channel was thresholded and a ROI including only cell containing regions was created. The Coloc 2 tool was used to calculate the Pearson correlation coefficient of the ROI between the different fluorescence channels. Cytofluorogram plotting was done using the imageJ plugin JACoP^63^.

### Statistics and reproducibility

The following experiments are representative of two independent biological replicates: Figs. 2d,h,  3b and 4a and Extended Data Figs. 1c, 2a,b,d,h, 3d, 4f, 5a,d,h, 7b,e, 8a,c,e, 9a,e and 10c,f,g.

The following experiments are representative of three independent biological replicates: Figs. 2a,f,g, 3c–e and 4d and Extended Data Figs. 2g, 3a, 4g, 6c–l, 7c,d,f,g, 8d and 10e.

The following experiments are representative of four independent biological replicates: Extended Data Figs. 1b, 2f, 3b, 4a and 9b.

Significant *P* values were validated using a two-tailed Student’s *t*-test or a non-parametric Mann–Whitney test.

### Reporting summary

Further information on research design is available in the Nature Research Reporting Summary linked to this paper.

## Online content

Any methods, additional references, Nature Research reporting summaries, source data, extended data, supplementary information, acknowledgements, peer review information; details of author contributions and competing interests; and statements of data and code availability are available at 10.1038/s41586-022-04834-7.

## Supplementary information

Supplementary Figures This file contains Supplementary Fig. 1, the uncropped scans of SDS–PAGE gels and blots shown in the study; and Supplementary Fig. 2, which shows MST analysis of interactions between Bam proteins and Tetra_*n*_.

Reporting Summary

Supplementary Tables 1–10 This zipped file contains Supplementary Tables 1–10 and a Supplementary Table guide which includes additional Supplementary Table references.

Supplementary Data This zipped file contains image source data.

Supplementary Video 1 BamA forms islands all across the outer membrane. 3D-SIM image of BamA labelling using monoclonal Fabs (heat map) and the binary map of the islands.

Supplementary Video 2 FepA biogenesis is mid-cell biased and cell cycle dependent. 3D-SIM image of FepA labelling after 5 minutes induction (heatmap) in dividing and non-dividing cells.

Supplementary Video 3 PG insertion pattern is cell cycle dependent. 3D-SIM image of PG labelling after 7 minutes incubation with HADA in dividing and non-dividing cells.

Supplementary Video 4 OMP and PG biogenesis patterns mirror one another. 3D-SIM images of FepA, BamA and PG at an intermediate stage of the cell cycle after 7 min of FepA induction. Cells were labelled using ColB–mCherry, anti-BamA–AF488 monoclonal Fabs and HADA.

Supplementary Video 5 Coordinated polar displacement of OMPs and PG in *K. pneumonia*. Time-lapse images of PG and OMP polar displacement during growth of *K. pneumoniae*. Cells were pre-labelled with CloDF13–AF488 and HADA and grown on M9 agar pads.

Supplementary Video 6 OMP biogenesis following aztreonam treatment. 3D-SIM image of FepA in aztreonam treated cells after 7 minutes induction (heat map).

## Data Availability

The data supporting the findings of this study are available within the paper and its Supplementary Information files. All the images displayed in this study, raw MST data and raw BAM activity data are available as source data files accompanying this manuscript. Raw uncropped gel images appear in Supplementary Fig. 1 and full MST controls appear in Supplementary Fig. 2. Materials and reagents are available upon request.
