# Supplementary material for: Peptidoglycan maturation controls outer membrane protein assembly
Source: Nature. 2022 Jun 15;606(7916):953–9. doi: 10.1038/s41586-022-04834-7 (PMC9242858; doi:10.1038/s41586-022-04834-7)
Supplement: Supplementary file 1 — This file contains Supplementary Fig. 1, the uncropped scans of SDS–PAGE gels and blots shown in the study; and Supplementary Fig. 2, which shows MST analysis of interactions between Bam proteins and Tetran. [file 41586_2022_4834_MOESM1_ESM.pdf]

nature portfolio

https://doi.org/10.1038/s41586-022-04834-7

Supplementary information

Peptidoglycan maturation controls outer membrane protein assembly

In the format provided by the
authors and unedited

SI Figure 1 (Fig. 2, Fig. 3, ED Fig. 7 and ED Fig. 10 related)

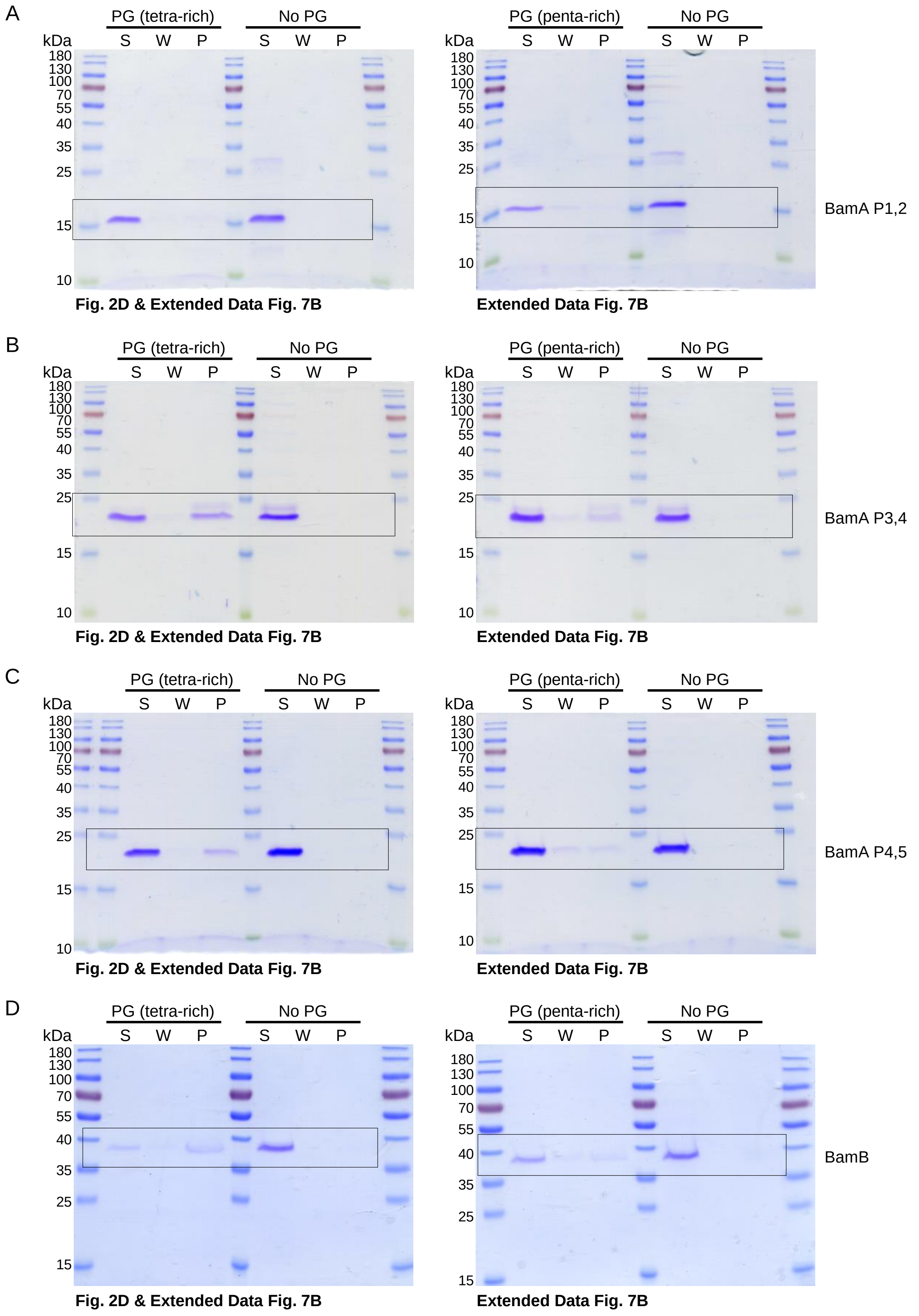

SI Figure 1 (Fig. 2, Fig. 3, ED Fig. 7 and ED Fig. 10 related)

E

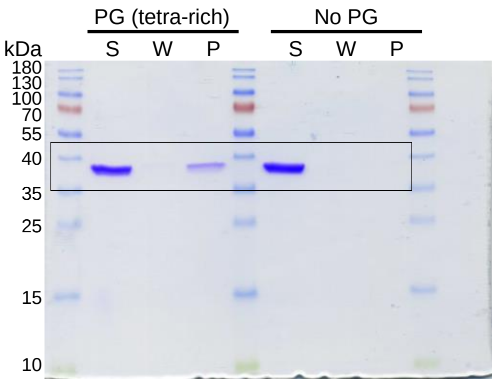

Fig. 2D & Extended Data Fig. 7B

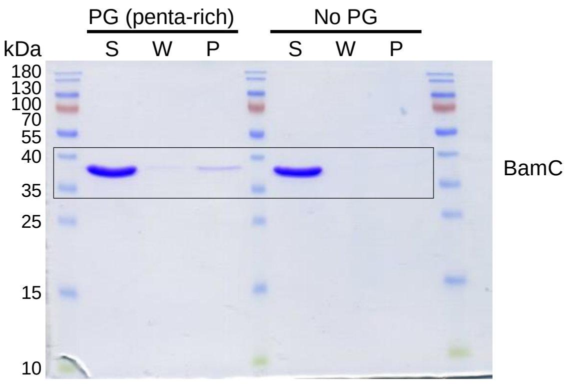

Extended Data Fig. 7B

F

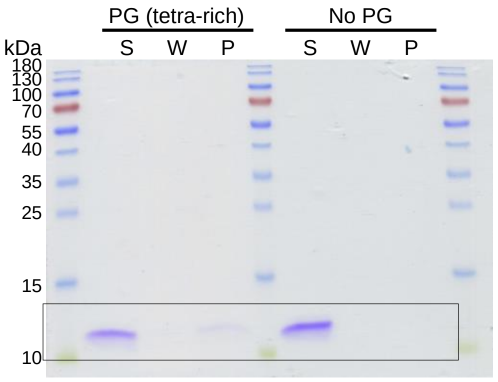

Fig. 2D & Extended Data Fig. 7B

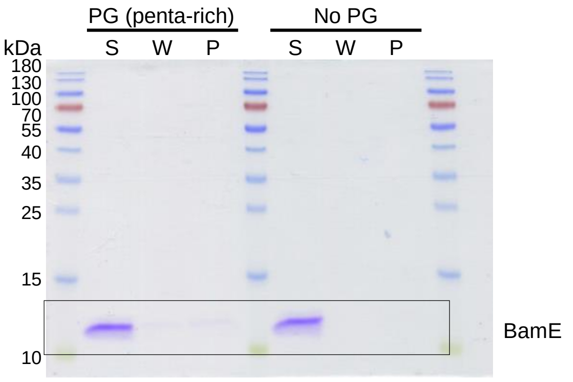

Extended Data Fig. 7B

G

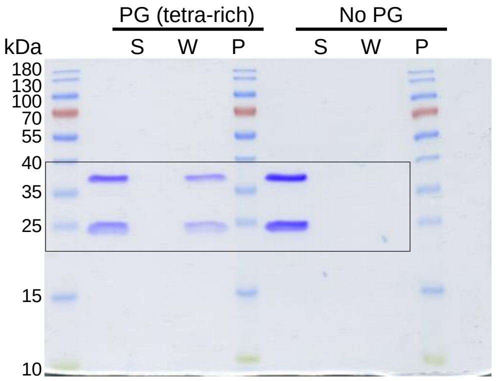

Fig. 2D & Extended Data Fig. 7B

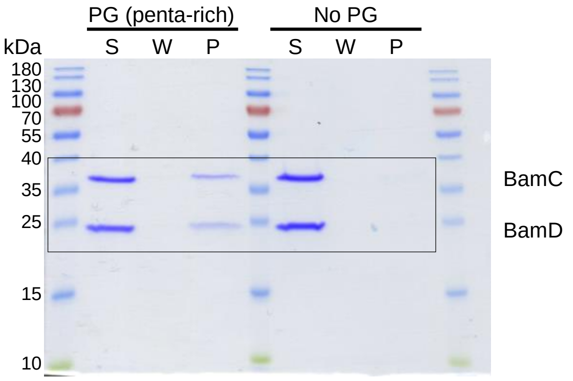

Extended Data Fig. 7B

H

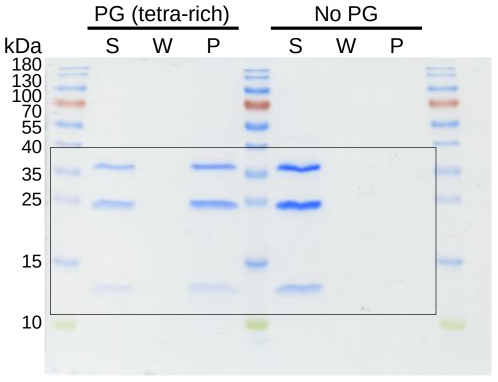

Fig. 2D & Extended Data Fig. 7B

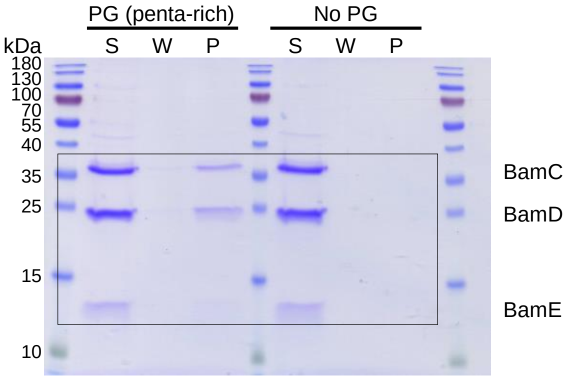

Extended Data Fig. 7B

SI Figure 1 (Fig. 2, Fig. 3, ED Fig. 7 and ED Fig. 10 related)

I

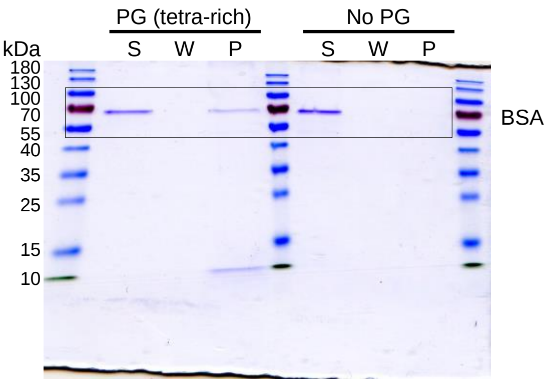

Fig. 2D

J

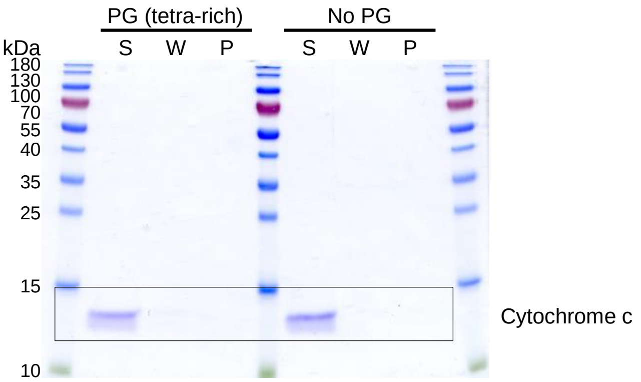

Fig. 2D

K

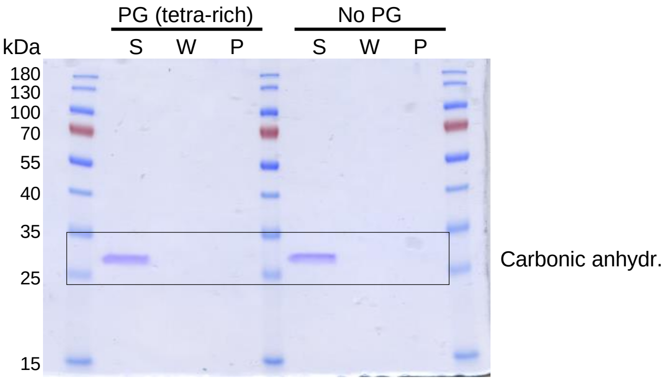

Fig. 2D

SI Figure 1 (Fig. 2, Fig. 3, ED Fig. 7 and ED Fig. 10 related)

L

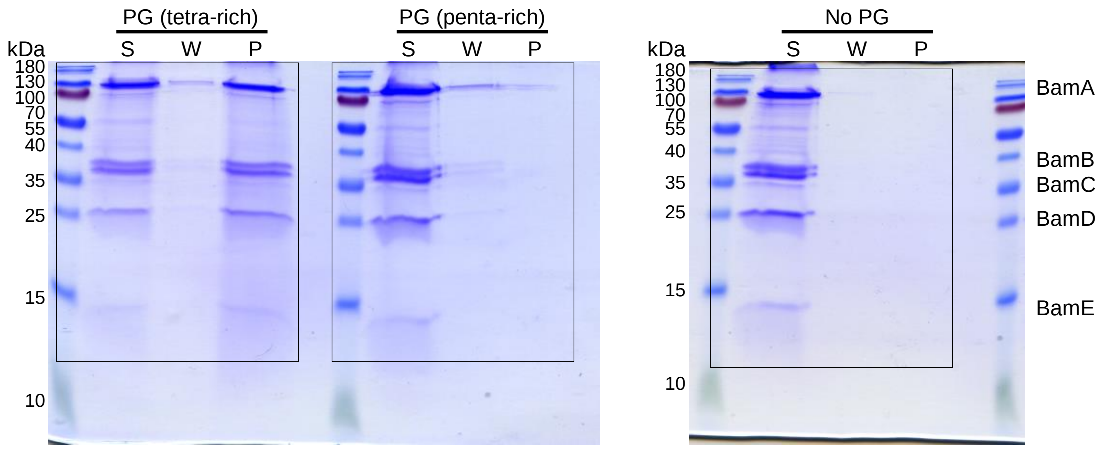

Fig. 3B **Fig. 3B**

M

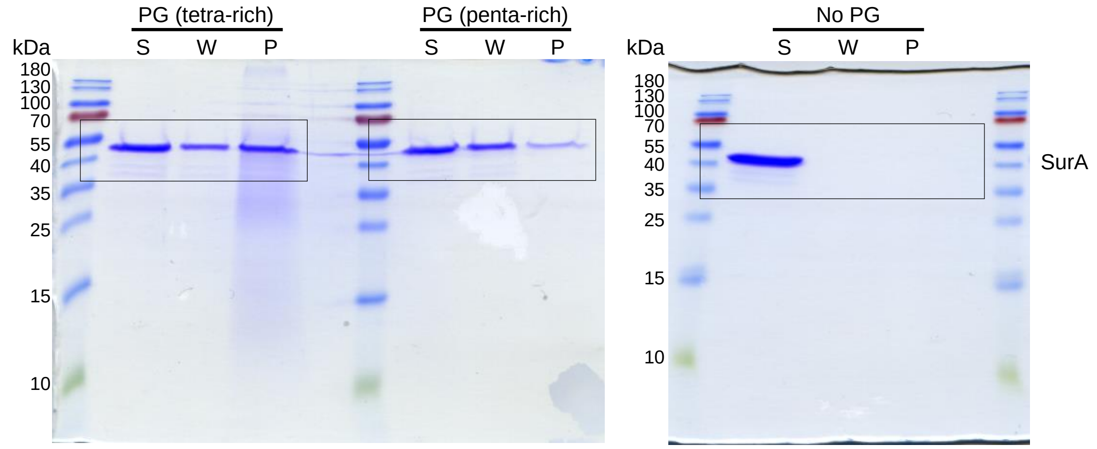

Extended Data Fig. 7E **Extended Data Fig. 7E**

N

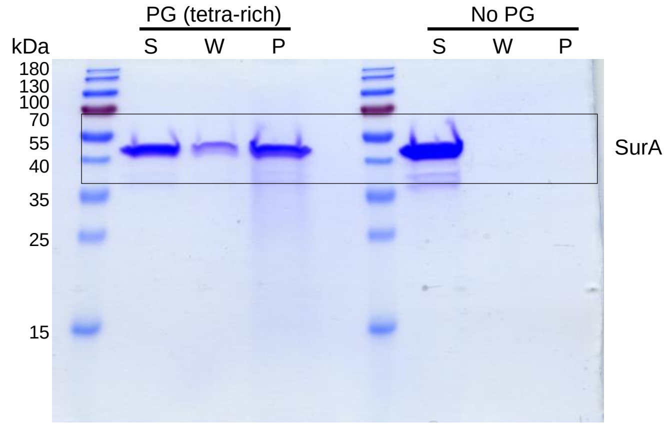

Extended Data Fig. 7F

SI Figure 1 (Fig. 2, Fig. 3, ED Fig. 7 and ED Fig. 10 related)

O

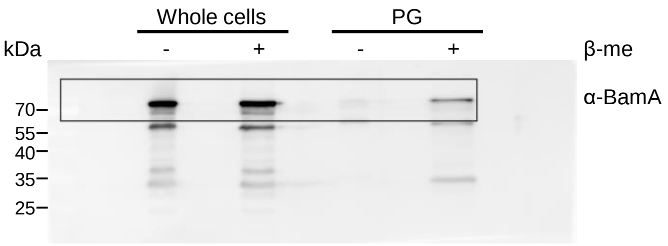

Fig. 2H

P

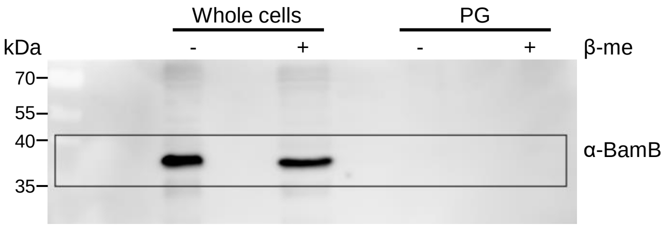

Fig. 2H

Q

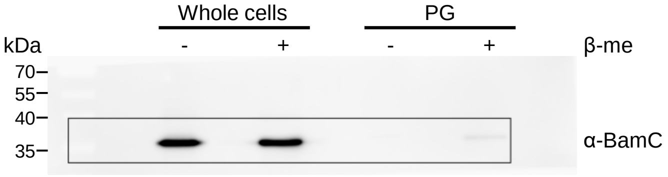

Fig. 2H

R

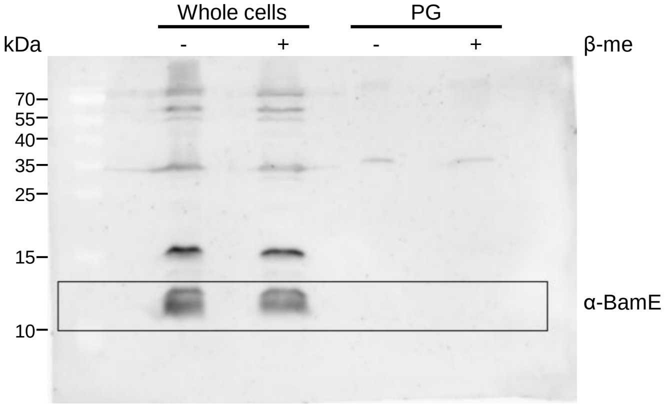

Fig. 2H

S

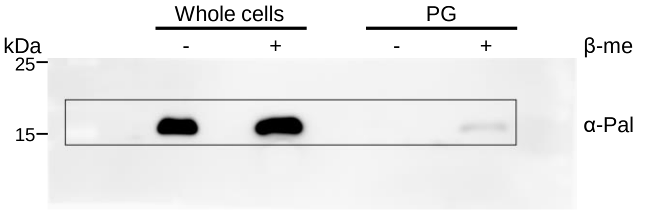

Fig. 2H

T

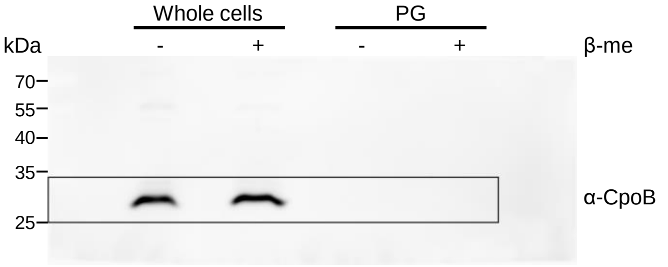

Fig. 2H

SI Figure 1 (Fig. 2, Fig. 3, ED Fig. 7 and ED Fig. 10 related)

U

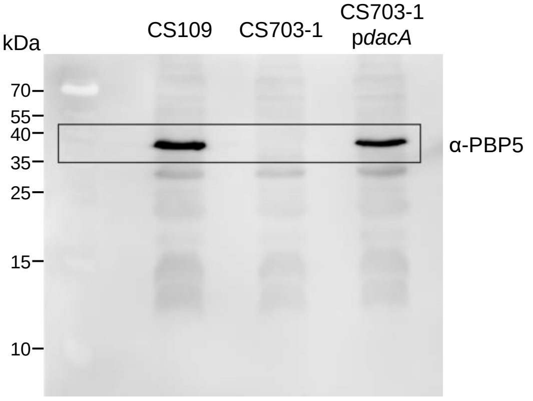

Extended Data Fig. 10C

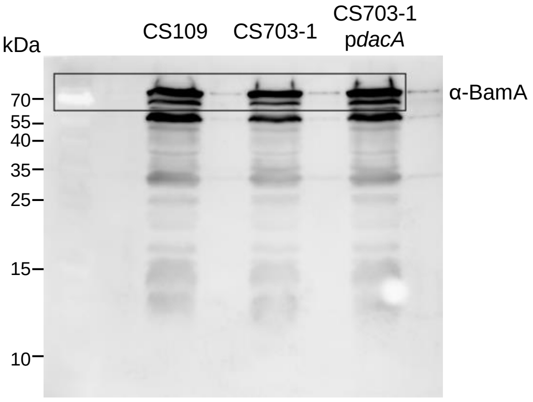

Extended Data Fig. 10C

V

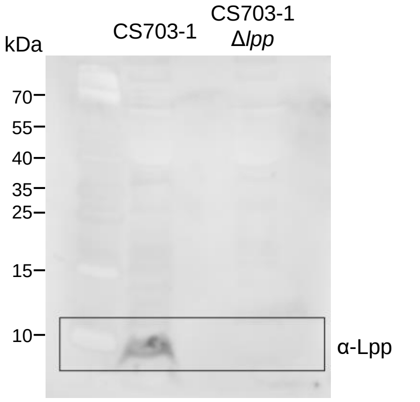

Extended Data Fig. 10F

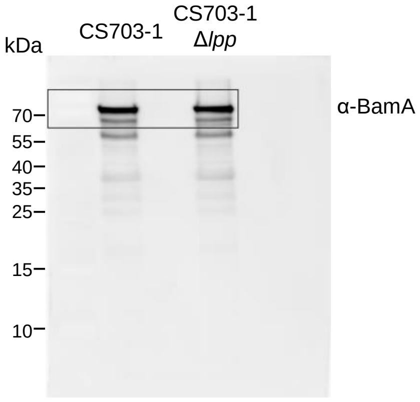

Extended Data Fig. 10F

W

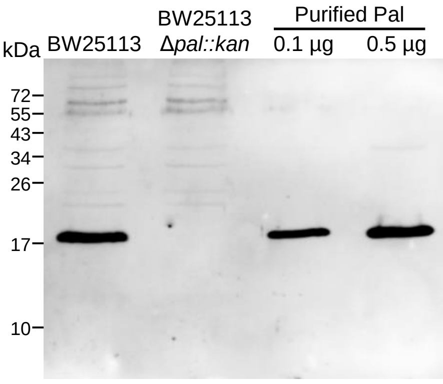

Fig. 2H

SI Figure 1. Uncropped scans of SDS-PAGE gels and blots shown in this study. (**A**) PG pull-down experiments for BamA P1,2 in the presence of tetrapeptide-rich PG (from MC1061, left panel) or pentapeptide-rich PG (from CS703-1, right panel). Uncropped gels for PG pull-down experiments performed in the same way for BamA P3,4 (**B**), BamA P4,5 (**C**), BamB (**D**), BamC (**E**), BamE (**F**), BamCD (**G**) and BamCDE (**H**) are also shown. (**I-K**) PG pull-down controls in the presence of tetrapeptide-rich PG from MC1061 performed with BSA (**I**), cytochrome c (**J**) and carbonic anhydrase (**K**). (**L-M**) PG pull-down experiments for BamABCDE (**L**) or SurA (**M**) in the presence of tetrapeptide-rich PG (from MC1061) or pentapeptide-rich PG (from CS703-1). A control sample containing no PG was analysed in parallel in the same way (right panel). (**N**) PG pull-down experiments to monitor PG binding of SurA in the presence of tetrapeptide-rich PG from MC1061 during *in vitro* BAM activity control experiments.

All PG pull-down experiments shown in this study were analysed by SDS-PAGE and Coomassie Blue staining. *S*, supernatant fraction; *W*, wash fraction; *P*, pellet fraction. The Main Figure or Extended Data Figure of reference in the main text is indicated below each individual panel. The cropped areas shown in Main Figures or Extended Data Figures in the main text are indicated for each uncropped gel scan as black boxes.

(**O**) Interaction of BamA with PG in *E. coli* MC1061. Cells were treated with DTSSP, then boiled in 8% SDS to isolate sacculi. Proteins cross-linked to PG were detected by specific antibodies after treatment with β-mercaptoethanol (β-me), which reverses the cross-links releasing proteins from PG. Interactions between PG and BamB (**P**), BamC (**Q**) and BamE (**R**) were analysed in the same way. Samples were also probed with Pal (**S**) and CpoB (**T**) antibodies as a positive and negative control, respectively. (**U**) Western Blot analysis showing ectopic production of PBP5 from p*dacA* in CS703-1. Expression of *dacA* was induced in LB with 0.2% arabinose, then cells were harvested and cell pellets analysed by SDS-PAGE and Western Blot

with specific PBP5 antibodies. Samples were also probed with BamA antibodies as loading control (bottom panel). (**V**) Western Blot analysis showing the absence of Lpp in CS703-1Δ*lpp*. Cells were grown in LB, then harvested and cell pellets analysed by SDS-PAGE and Western Blot with specific Lpp antibodies. Samples were also probed with BamA antibodies as loading control (bottom panel). (**W**) Western Blot analysis showing the validation of Pal antibodies in *E. coli* BW25113 and a *pal* deletion strain. Cells were grown in LB, harvested and cell pellets analysed by SDS-PAGE and Western Blot with antibodies raised against purified Pal protein, also loaded on the same gel as control.

The Main Figure or Extended Data Figure of reference in the main text is indicated below each individual panel. The cropped areas shown in Main Figures or Extended Data Figures in the main text are indicated for each uncropped blot scan as black boxes.

SI Figure 2 (Fig. 2 and ED Fig. 6 related)

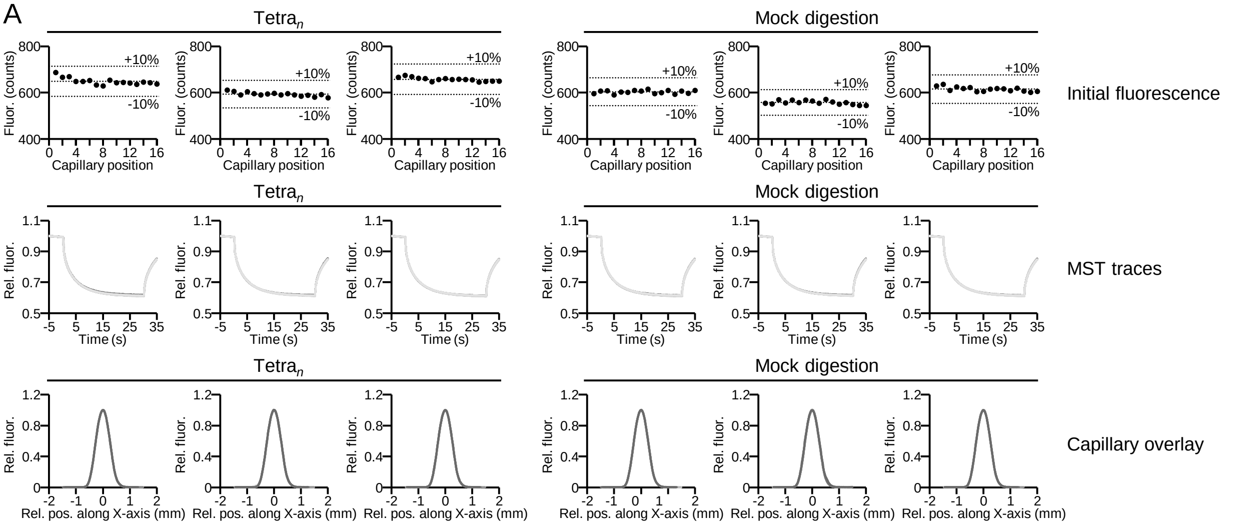

Fig. 2F & Extended Data Fig. 6C

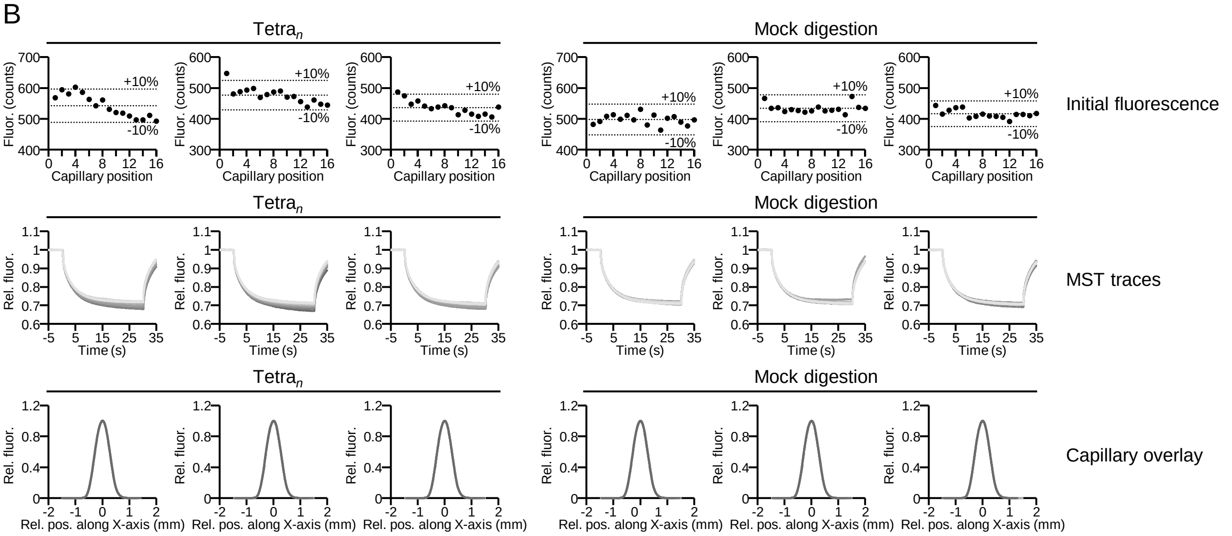

Fig. 2F & Extended Data Fig. 6D

SI Figure 2 (Fig. 2 and ED Fig. 6 related)

C

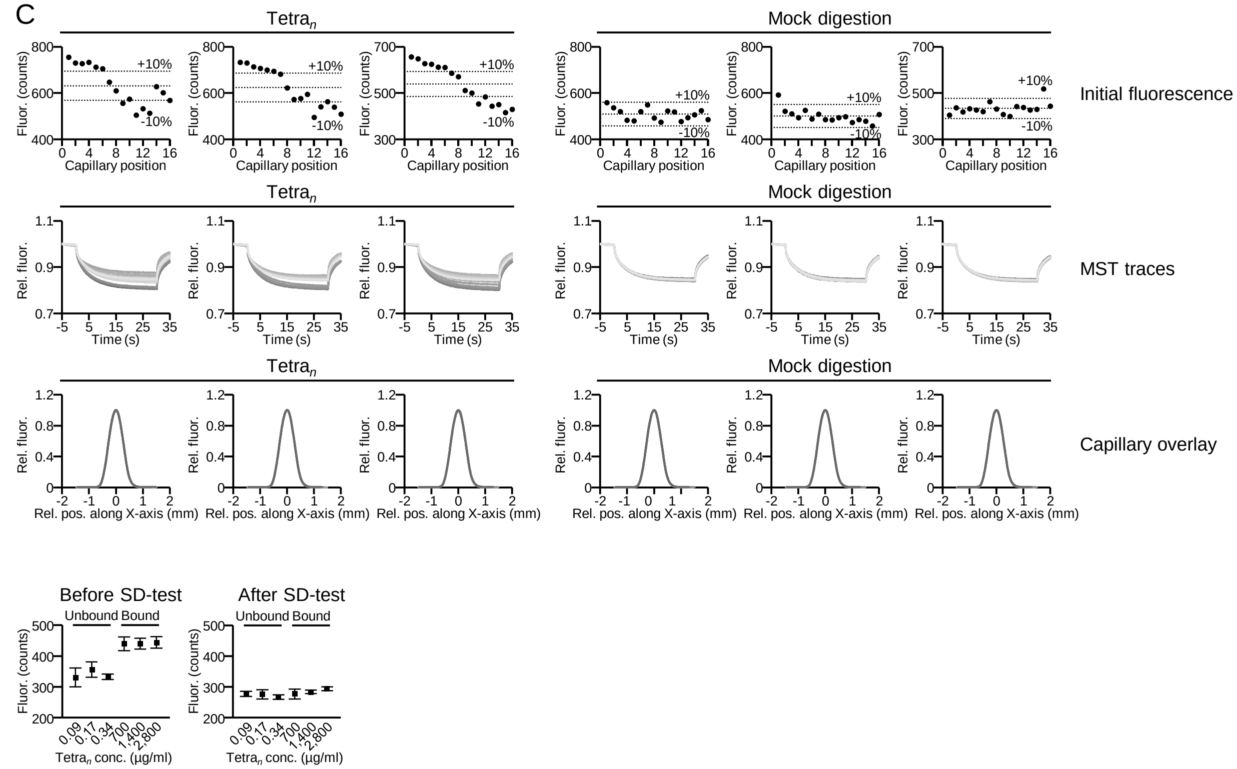

Fig. 2F & Extended Data Fig. 6E

D

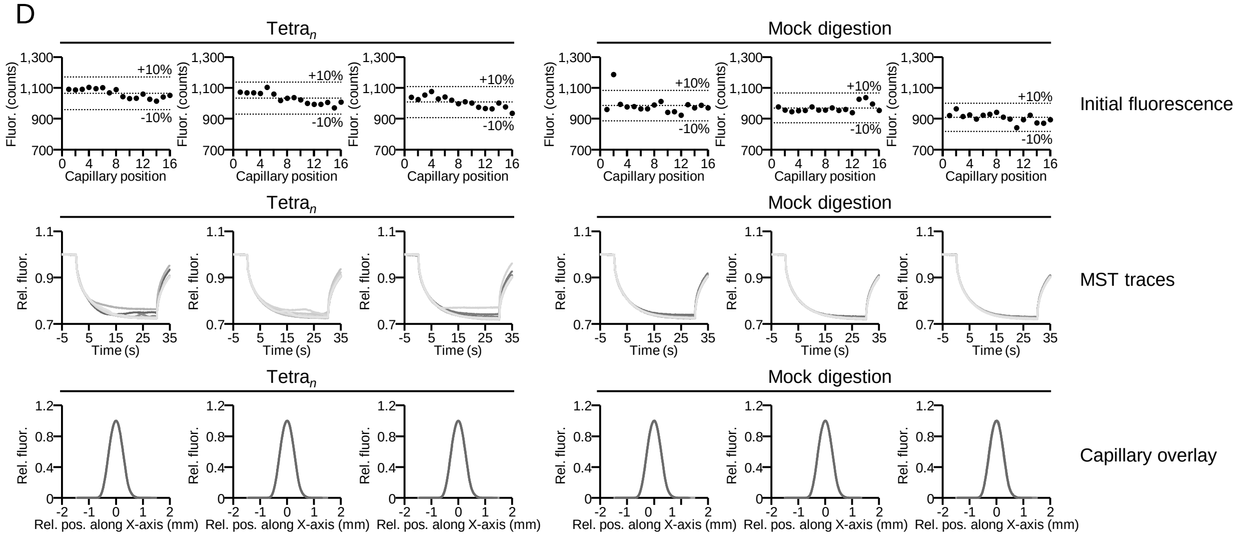

Extended Data Fig. 6F

SI Figure 2 (Fig. 2 and ED Fig. 6 related)

E

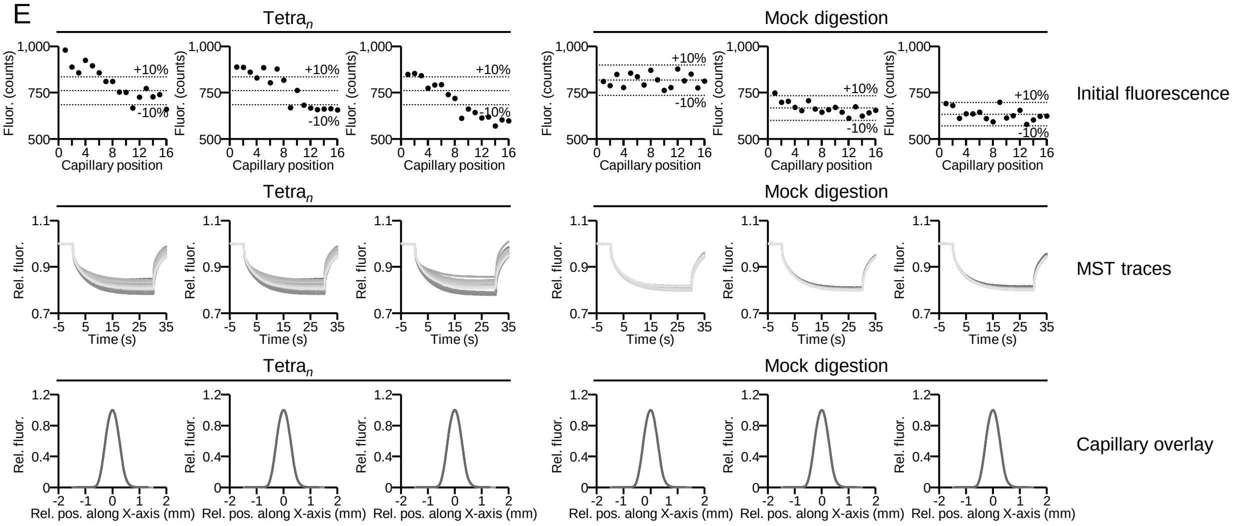

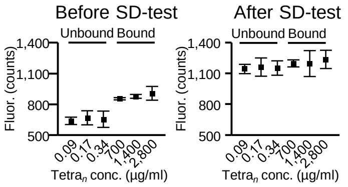

Extended Data Fig. 6G

F

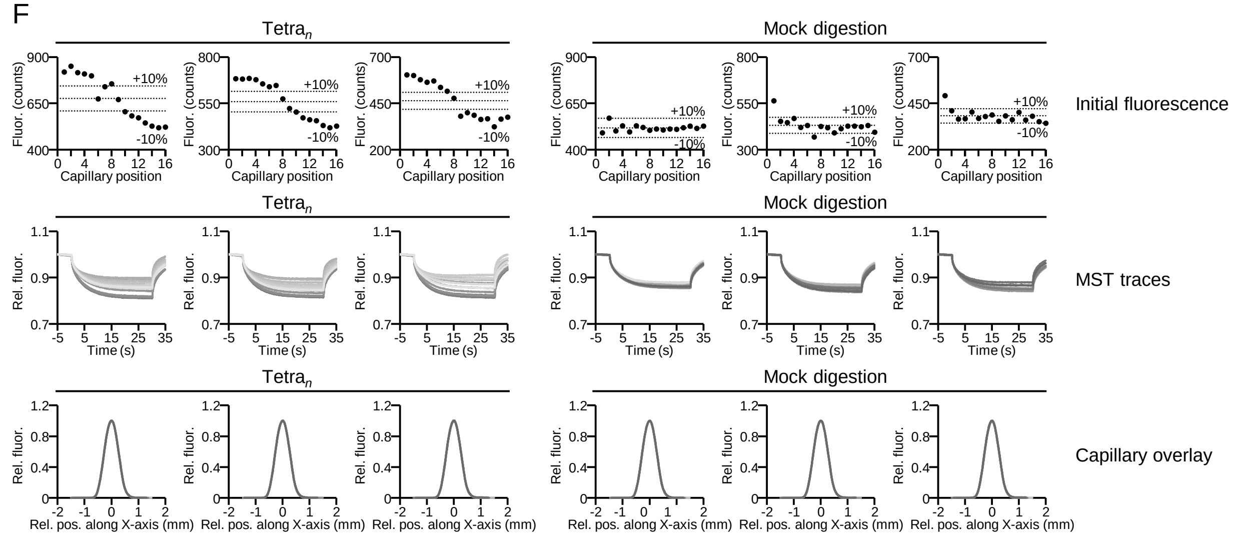

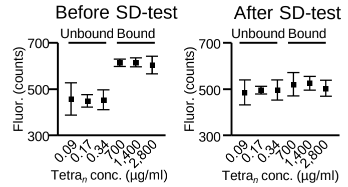

Extended Data Fig. 6H

SI Figure 2 (Fig. 2 and ED Fig. 6 related)

G

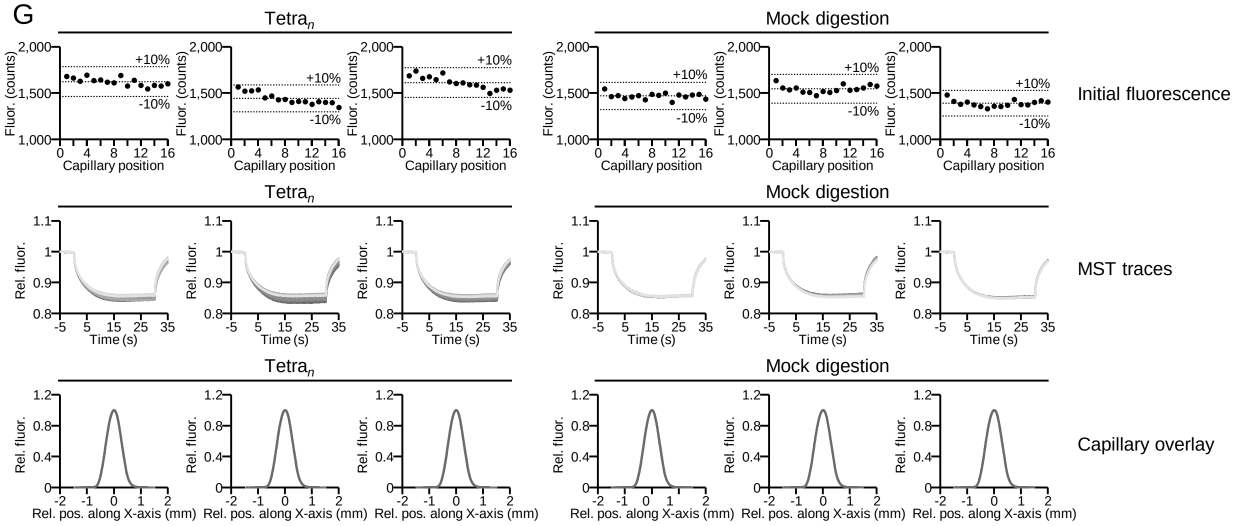

Extended Data Fig. 6I

H

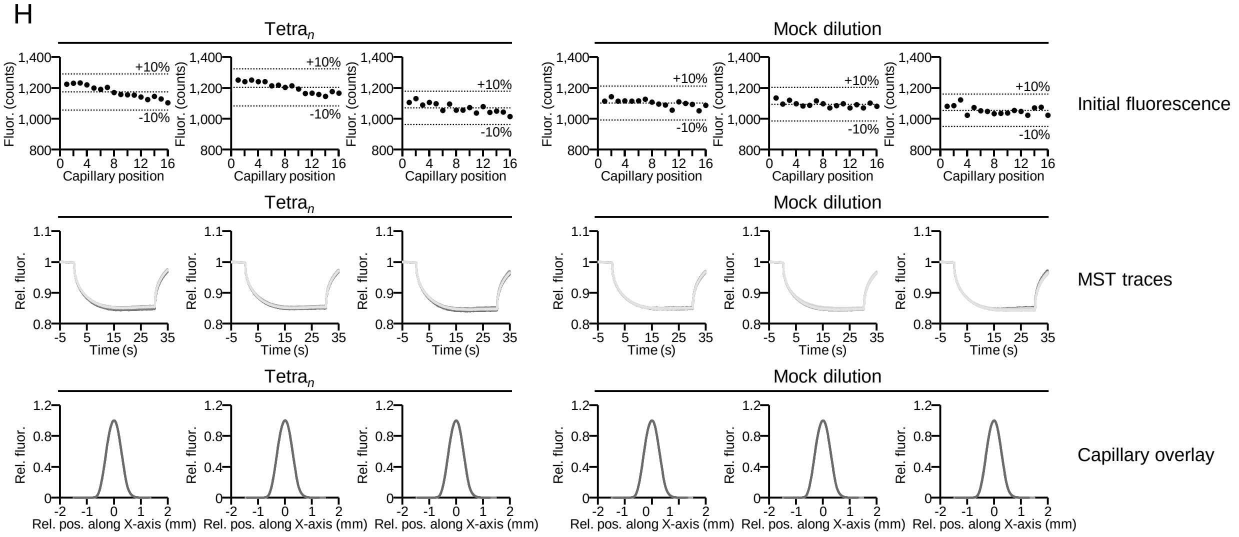

Extended Data Fig. 6J

SI Figure 2 (Fig. 2 and ED Fig. 6 related)

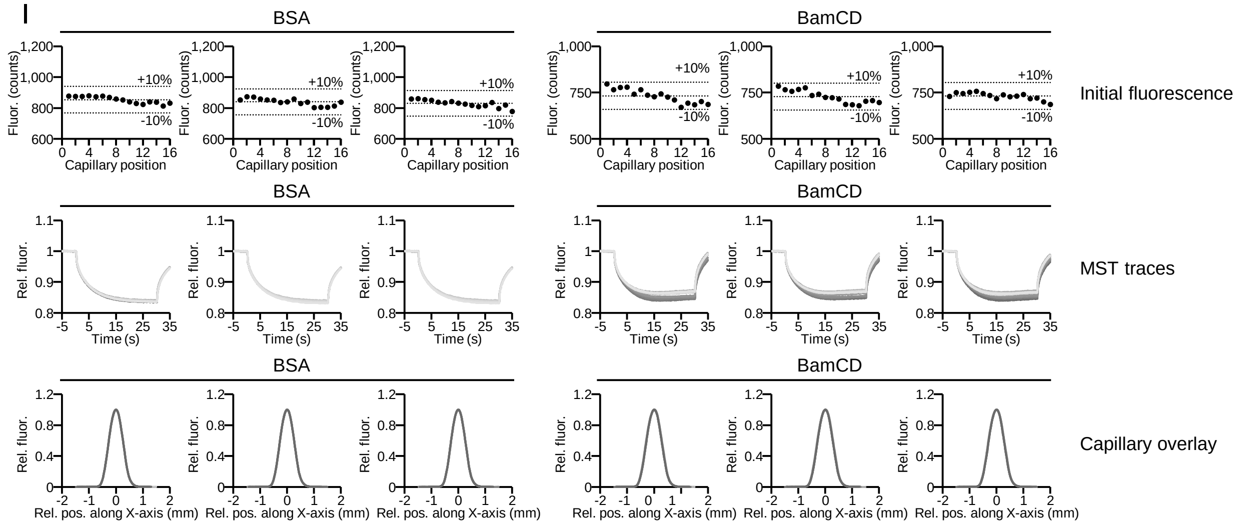

Extended Data Fig. 6K

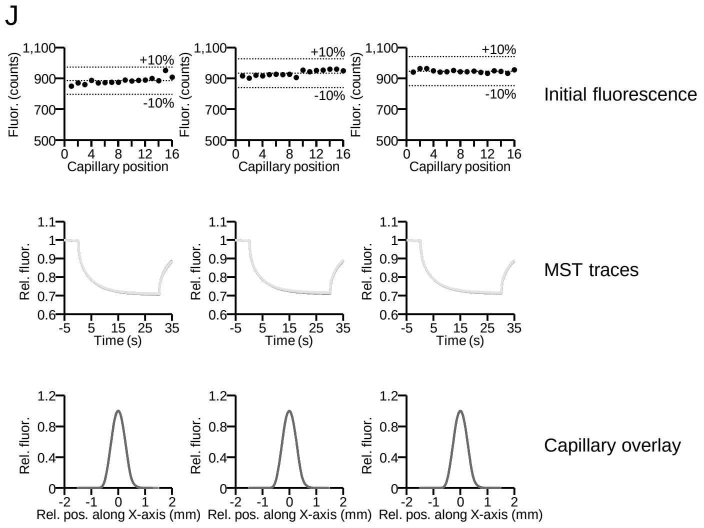

Extended Data Fig. 6L

SI Figure 2. MST analysis of interactions between Bam proteins and Tetra$_n$. (**A**) MST controls for BamA P1,2 and Tetra$_n$. Fig. 2F and Extended Data Fig. 6 show that BamA P1,2 does not interact with Tetra$_n$. Here, initial fluorescence (top graphs; average fluorescence ± 10% variation is indicated as dotted lines for each replicate), MST traces (middle graphs) and capillary overlay (bottom graphs) from three independent experiments with Tetra$_n$ (left panel) or the mock PG digest (right panel) are shown. (**B-H**) Analysis of initial fluorescence, MST traces and capillary scans for BamA P3,4 (**B**), BamA P4,5 (**C**), BamB (**D**), BamC (**E**), BamE (**F**), BamCD (**G**) and BamCDE (**H**) from three independent experiments with Tetra$_n$ (left panels) or the mock PG digest (right panels). Variations in initial fluorescence greater than ± 10% of the average fluorescence along the serial dilution prior to the application of the temperature gradient for BamA P4,5 (**C**) BamC (**E**) and BamE (**F**) were confirmed to be ligand-dependent by SD-tests (bottom graphs), by analysing the fluorescence in the first three (bound state) and last three (unbound state) capillaries of each series, before and after denaturation at 95°C in 2× SD-mix (see Methods). (**I**) Analysis of initial fluorescence, MST traces and capillary scans for control experiments performed with fluorescent-labelled BSA or BamCD in the presence of Tetra$_n$. (**J**) Analysis of initial fluorescence, MST traces and capillary scans for control experiments performed with free fluorescent Red-NHS dye and no protein in the presence of Tetra$_n$.

The Main Figure or Extended Data Figure of reference in the main text is indicated below each individual panel.
