# Supplementary material for: Peptidoglycan maturation controls outer membrane protein assembly
Source: Nature. 2022 Jun 15;606(7916):953–9. doi: 10.1038/s41586-022-04834-7 (PMC9242858; doi:10.1038/s41586-022-04834-7)
Supplement: Supplementary file 2 — Reporting Summary [file 41586_2022_4834_MOESM2_ESM.pdf]

nature portfolio

Corresponding author(s): Colin Kleanthous, Waldemar Vollmer

Last updated by author(s): 05/04/2022

Reporting Summary

Nature Portfolio wishes to improve the reproducibility of the work that we publish. This form provides structure for consistency and transparency in reporting. For further information on Nature Portfolio policies, see our Editorial Policies and the Editorial Policy Checklist.

Statistics

For all statistical analyses, confirm that the following items are present in the figure legend, table legend, main text, or Methods section.

n/a	Confirmed	
☐	☒	The exact sample size (n) for each experimental group/condition, given as a discrete number and unit of measurement
☐	☒	A statement on whether measurements were taken from distinct samples or whether the same sample was measured repeatedly
☐	☒	The statistical test(s) used AND whether they are one- or two-sided<br>*Only common tests should be described solely by name; describe more complex techniques in the Methods section.*
☒	☐	A description of all covariates tested
☒	☐	A description of any assumptions or corrections, such as tests of normality and adjustment for multiple comparisons
☐	☒	A full description of the statistical parameters including central tendency (e.g. means) or other basic estimates (e.g. regression coefficient) AND variation (e.g. standard deviation) or associated estimates of uncertainty (e.g. confidence intervals)
☐	☒	For null hypothesis testing, the test statistic (e.g. F, t, r) with confidence intervals, effect sizes, degrees of freedom and P value noted<br>*Give P values as exact values whenever suitable.*
☒	☐	For Bayesian analysis, information on the choice of priors and Markov chain Monte Carlo settings
☒	☐	For hierarchical and complex designs, identification of the appropriate level for tests and full reporting of outcomes
☐	☒	Estimates of effect sizes (e.g. Cohen's d, Pearson's r), indicating how they were calculated

Our web collection on statistics for biologists contains articles on many of the points above.

Software and code

Policy information about availability of computer code

Data collection	Data was collected using the integrated image acquisition software with each microscope. MST data were collected on a Monolith NT.115.
Data analysis	Images were analyzed using using ImageJ (v1.52p), MicrobeJ plugin 27 (v5.13m) and the JACoP plugin. MST data were analyzed by the MO.Affinity Analysis (x64) V2.1.2 (NanoTemper Technologies). BAM activity data were analyzed with Microsoft Excel 2016. Values for tetrapeptide-rich and pentapeptide-rich PG were estimated using the online MyCurveFit tool (https://mycurvefit.com/)

For manuscripts utilizing custom algorithms or software that are central to the research but not yet described in published literature, software must be made available to editors and reviewers. We strongly encourage code deposition in a community repository (e.g. GitHub). See the Nature Portfolio guidelines for submitting code & software for further information.

Data

Policy information about availability of data

All manuscripts must include a data availability statement. This statement should provide the following information, where applicable:

- Accession codes, unique identifiers, or web links for publicly available datasets
- A description of any restrictions on data availability
- For clinical datasets or third party data, please ensure that the statement adheres to our policy

The data supporting the findings of this study are available within the paper and its Supplementary Information files. All the images displayed in this study, raw MST data and raw BAM activity data are available as source data files accompanying this manuscript. Raw uncropped gel images appear in SI Figure 1 and full MST controls appear in SI Figure 2. Materials & reagents are available upon request.

Field-specific reporting

Please select the one below that is the best fit for your research. If you are not sure, read the appropriate sections before making your selection.

☒ Life sciences ☐ Behavioural & social sciences ☐ Ecological, evolutionary & environmental sciences

For a reference copy of the document with all sections, see nature.com/documents/nr-reporting-summary-flat.pdf

Life sciences study design

All studies must disclose on these points even when the disclosure is negative.

Sample size	The number of bacterial cells analyzed is the described sample size. When localization of BAM clusters is described, the sample size is the number of clusters (islands) detected. The sample size wasn't predetermined. All the cells from at least 3 fields of view were analyzed however this number varies. In cases where fields of view were sparsely populated, additional ones were analyzed to achieve a similar sample size.
Data exclusions	For microscopy experiments, bacterial clusters (cells touching one another) were excluded in order to prevent miscalculation of the fluorescence intensity or distribution. In MST in few experiments few capillaries (1 or 2 of a series of 16 capillaries of a serial dilution) showed signs of protein aggregation (recognized by a bumpy MST curve) and these were excluded from the analysis.
Replication	All the microscopy experiments were carried out at least twice and the results were reproducible. The number of biological replicates is indicated in the legends and Methods section. MST and BAM activity experiments were done in triplicate.
Randomization	For microscopy experiments at least 3 fields of view from each dataset were randomly used and all the the non-clustered cells (see above) were analyzed. Samples were allocated into experimental groups according to their genetic background, growth condition (based on OD600), cell cycle condition (based on cell length) or treatment with different antibiotics.
Blinding	For all fluorescent microscopy images a corresponding transillumination image was taken and images were picked for analysis based upon the transillumination images (which do not display the analyzed data). Blinding the genetic background, cell cycle condition or antibiotic treatment wasn't possible in most cases since the different groups display noticeable morphological characteristics.

Reporting for specific materials, systems and methods

We require information from authors about some types of materials, experimental systems and methods used in many studies. Here, indicate whether each material, system or method listed is relevant to your study. If you are not sure if a list item applies to your research, read the appropriate section before selecting a response.

Materials & experimental systems

n/a	Involved in the study	
☐	☒	Antibodies
☒	☐	Eukaryotic cell lines
☒	☐	Palaeontology and archaeology
☒	☐	Animals and other organisms
☒	☐	Human research participants
☒	☐	Clinical data
☒	☐	Dual use research of concern

Methods

n/a	Involved in the study	
☒	☐	ChIP-seq
☒	☐	Flow cytometry
☒	☐	MRI-based neuroimaging

Antibodies

Antibodies used	αBamA - MAB2 (monoclonal) : Genentech, 29E9. (Storek et al., 2018)<br><br>α-BamA, α-BamB, α-BamC, α-BamE (Rodríguez-Alonso et al., 2020) and α-Lpp (Asmar et al., 2017) antibodies were received from the Collet group (UCLouvain, Belgium); α-CpoB (Gray et al., 2015) and α-Pal antibodies were received from Alexander Egan (Newcastle University, UK); Rabbit polyclonal α-PBP5 antibodies were raised against purified PBP5 protein (Eurogentec, Belgium).<br>α-rabbit goat HRP- IgG antibodies were purchased from Sigma Aldrich (catalog number 12-348)
Validation	α-BamA- MAB2 (monoclonal): Storek et al., (2018) doi: 10.1073/pnas.1800043115. This study Ext data figure 1C<br>α-BamA - Rodriguez-Alonso et al., (2020) doi: 10.1038/s41589-020-0575-0<br>α-BamB - Rodriguez-Alonso et al., (2020) doi: 10.1038/s41589-020-0575-0<br>α-BamC - Rodriguez-Alonso et al., (2020) doi: 10.1038/s41589-020-0575-0<br>α-BamE - Rodriguez-Alonso et al., (2020) doi: 10.1038/s41589-020-0575-0<br>α-CpoB - Validated against a ΔcpoB deletion strain (Gray et al., 2015) doi: 10.7554/eLife.07118<br>α-Lpp - Validated against a Δlpp deletion strain (SI Fig. 1V)<br>α-Pal - validated against a Δpal deletion strain (SI Fig. 1W).

α-PBP5 - Validated using the multiple carboxypeptidase mutant lacking PBP5, and the same strain expressing PBP5 from plasmid (SI Fig. 1U)
