# Supplementary material for: Peptidoglycan maturation controls outer membrane protein assembly
Source: Nature. 2022 Jun 15;606(7916):953–9. doi: 10.1038/s41586-022-04834-7 (PMC9242858; doi:10.1038/s41586-022-04834-7)
Supplement: Supplementary file 3 — This zipped file contains Supplementary Tables 1–10 and a Supplementary Table guide which includes additional Supplementary Table references. [file 41586_2022_4834_MOESM3_ESM.zip › SI Table 1.pdf]

SI Table 1. Bacterial strains used in this study.

Strain	Features	Reference
E. coli		
BW25113	*lacI*$^{+}$*rrnB*$_{T14}$ Δ*lacZ*$_{WJ16}$ *hsdR*514 Δ*araBAD*$_{AH33}$ Δ*rhaBAD*$_{LD78}$ *rph-1* Δ*(araB–D)567* Δ*(rhaD–B)568* Δ*lacZ4787*(::*rrnB-3*) *hsdR514 rph-1*	Datsenko & Wanner, 2000[64]
BW25113Δ6LDT	BW25113 Δ*ldtA* Δ*ldtB* Δ*ldtC* Δ*ldtD* Δ*ldtE* Δ*ldtF*	Kuru *et al.*, 2017[30]
CS109	W1485 *glnV rpoS rph*	Denome *et al.*, 1999[39]
CS12-7	CS109 Δ*dacA*	Denome *et al.*, 1999[39]
CS703-1	CS109 Δ*mrcA* Δ*dacB* Δ*dacA* Δ*dacC* Δ*pbpG* Δ*ampC* Δ*ampH*	Meberg *et al.*, 2001[38]
CS703-1Δ*lpp*	CS109 Δ*mrcA* Δ*dacB* Δ*dacA* Δ*dacC* Δ*pbpG* Δ*ampC* Δ*ampH* Δ*lpp*	Dr Christian Otten (Newcastle University)
GNE 4077	BW25113 *ompT*, *waaD*::Cm BamA_barr (K. pneumoniae)::Gm	Storek et al., 2018[13]
MC1061	K-12 F^{-} λ^{-} Δ*(ara-leu)7697* [*araD139*]B/r Δ*(codB-lacI)3 galK16 galE15* e14– *mcrA0 relA1 rpsL150*(Str^{R}) *spoT1 mcrB1 hsdR2(r*$^{-}$*m*$^{+}$*)*	Casadaban & Cohen, 1980[65]
RK5016	MC4100, *metE70, argH, btuB, recA*	Heller et al., 1985[66]
PB114	Δ*minB*::Km(R). dadR1, trpE61, trpA62, tna5, purB, L-+.	de Boer et al., 1989[67]
K. pneumoniae		
TU11	Clinical isolate	Loraine et al., 2018[68]
P. aeruginosa		
PAO1	Clinical isolate	Jacobs et al. 2003[69]
