# Supplementary material for: Peptidoglycan maturation controls outer membrane protein assembly
Source: Nature. 2022 Jun 15;606(7916):953–9. doi: 10.1038/s41586-022-04834-7 (PMC9242858; doi:10.1038/s41586-022-04834-7)
Supplement: Supplementary file 3 — This zipped file contains Supplementary Tables 1–10 and a Supplementary Table guide which includes additional Supplementary Table references. [file 41586_2022_4834_MOESM3_ESM.zip › SI Table 2.pdf]

SI Table 2. Plasmids used in this study.

Plasmid	Application/Description	Reference
pEE04	pBAD myc HisB-FepA	Khait *et al.*, 2021[27]
pNGH015	pBAD myc His-B-BtuB	Housden *et al.,* 2013[70]
pNGH206	pET21a-$\Delta^{2\text{-}61}$ ColE9 K469C	This study
pNGH382	pQE-2-His_6-Im9-$CloDF13_{301\text{-}460}$	This study
pVY23	pET21a-S2NTD-mCherry-His	This study
pQE70-POTRA(1-2)	Purification of BamA POTRA(1-2)	Knowles *et al.*, 2008[58]
pET26b-POTRA(3-4)	Purification of BamA POTRA(3-4)	This study
pET26b-POTRA(4-5)	Purification of BamA POTRA(4-5)	This study
pET22b-*bamB*	Purification of BamB	Rossiter *et al.*, 2011[71]
pET16b-*bamC*	Purification of BamC	Knowles *et al.*, 2009[72]
pET22b-*bamE*	Purification of BamE	Knowles *et al.*, 2011[73]
pSK46	Purification of full-length BamCD	Hagan *et al.*, 2010[40]
pBamE-His	Purification of full-length BamE	Sklar *et al.*, 2007[74]
pJH114	Purification of BamABCDE	Roman-Hernandez *et al.*, 2014[41]
pSK257	Purification of SurA	Hagan *et al.*, 2010[40]
pCH18	Purification of OmpT	Hagan *et al.*, 2010[40]
pMN86	Purification of MepM	Singh *et al.*, 2012[33]
pBAD33	Construction of pBAD33-*dacA*	Guzman *et al.*, 1995[75]
pBAD33-*dacA*	PBP5 complementation	This study
