# Supplementary material for: Peptidoglycan maturation controls outer membrane protein assembly
Source: Nature. 2022 Jun 15;606(7916):953–9. doi: 10.1038/s41586-022-04834-7 (PMC9242858; doi:10.1038/s41586-022-04834-7)
Supplement: Supplementary file 3 — This zipped file contains Supplementary Tables 1–10 and a Supplementary Table guide which includes additional Supplementary Table references. [file 41586_2022_4834_MOESM3_ESM.zip › SI Table 3.pdf]

SI Table 3. Oligonucleotides used in this study.

Name	Sequence (5'→3')	Application
dacA_F	CGGGGATCCTCTAGACATCACGGATGTCGTAGTTCAG	Amplification of *dacA* from *E. coli* BW25113
dacA_R	AAAACAGCCAAGCTTTTAACCAAACCAGTGATGGAAC	Amplification of *dacA* from *E. coli* BW25113
pBAD33_FR	ACGACATCCGTGATGTCTAGAGGATCCCCGGGTACCG	Cloning of *dacA* into pBAD33
pBAD33_RF	CACTGGTTTGGTTAAAAGCTTGGCTGTTTTGGCGG	Cloning of *dacA* into pBAD33
pBAD33_seq_F1	CGCAACTCTCTACTGTTTCTC	Sequencing of pBAD33-*dacA*
dacA_seq_R1	GTCGACATTCAGGCTGTTATC	Sequencing of pBAD33-*dacA*
dacA_seq_F2	CGCGAGATATGGCGCTGATC	Sequencing of pBAD33-*dacA*
pBAD33_seq_R2	CTGCCGCCAGGCAAATTCTG	Sequencing of pBAD33-*dacA*
pVY23_ins_F	GGTGGAGGCGGACTACAAGGGCGGATCTATGGTGAGCAAGGGCGAGGAAGATAACATG	Construction of pVY23
pVY23_ins_R	CAGTGGTGGTGGTGGTGGTGGCCCTGAAAATACAGGTTTTCCTCGAGCTTGTACAGCTCGTCCATGCCGCCG	Construction of pVY23
pVY23_vec_F	CGGCATGGACGAGCTGTACAAGCTCGAGGAAAACCTGTATTTTCAGGGCCACCACCACCACCACCACTGAGATC	Construction of pVY23
pVY23_vec_R	CTTCCTCGCCCTTGCTCACCATAGATCCGCCCTTGTAGTCCGCCTCCACCTCAGC	Construction of pVY23
