# Supplementary material for: Peptidoglycan maturation controls outer membrane protein assembly
Source: Nature. 2022 Jun 15;606(7916):953–9. doi: 10.1038/s41586-022-04834-7 (PMC9242858; doi:10.1038/s41586-022-04834-7)
Supplement: Supplementary file 3 — This zipped file contains Supplementary Tables 1–10 and a Supplementary Table guide which includes additional Supplementary Table references. [file 41586_2022_4834_MOESM3_ESM.zip › SI Table 4.pdf]

SI Table 4. Antibodies and engineered bacteriocins used in this study.

Name	Type	Dilution	Reference
αBamA - MAB2	FAB, monoclonal	200 nM	This study
α-BamA	Polyclonal	1:40000	Rodríguez-Alonso *et al.*, 2020[76]
α-BamB	Polyclonal	1:3000	Rodríguez-Alonso *et al.*, 2020[76]
α-BamC	Polyclonal	1:20000	Rodríguez-Alonso *et al.*, 2020[76]
α-BamE	Polyclonal	1:1500	Rodríguez-Alonso *et al.*, 2020[76]
α-CpoB	Polyclonal	1:2500	Gray *et al.*, 2015[36]
α-Lpp	Polyclonal	1:3000	Asmar *et al.*, 2017[77]
α-Pal	Polyclonal	1:2500	Alexander Egan (Newcastle University)
α-PBP5	Polyclonal	1:1000	This study
α-rabbit goat HRP- IgG	Polyclonal	1:5000	Sigma-Aldrich No 12-348
ColE9-AF^{488}	Colicin	200 nM	Rassam et al., 2015[8]
ColB-GFP	Colicin	200 nM	Cohen-Khait *et al.*, 2021[27]
ColB-mCherry	Colicin	200 nM	Cohen-Khait *et al.*, 2021[27]
CloDF13-AF^{488}	Klebicin	200 nM	This study
PyoS2-mCherry	Pyocin	200 nM	This study
$PyoS5_{1-315}$-AF^{488}	Pyocin	200 nM	Behrens *et al.*, 2020[55]
