# Supplementary material for: Peptidoglycan maturation controls outer membrane protein assembly
Source: Nature. 2022 Jun 15;606(7916):953–9. doi: 10.1038/s41586-022-04834-7 (PMC9242858; doi:10.1038/s41586-022-04834-7)
Supplement: Supplementary file 3 — This zipped file contains Supplementary Tables 1–10 and a Supplementary Table guide which includes additional Supplementary Table references. [file 41586_2022_4834_MOESM3_ESM.zip › SI Table Guide_ESM.pdf]

SI Table Legends

SI Table 1. Bacterial strains used in this study. The relevant features of the strains and references are indicated.

SI Table 2. Plasmids used in this study. With information about the application of the plasmids, descriptions and references.

SI Table 3. Oligonucleotides used in this study. Contains the name, sequence and application of the oligonucleotides.

SI Table 4. Antibodies and engineered bacteriocins used in this study. Details the type of the antibodies and bacteriocins, their dilution in the experiments and the references.

SI Table 5. Source data for MST analysis of interactions between BamA POTRA domain constructs and Tetra$_n$ (Fig. 2). Proteins labelled with Red-NHS were incubated with a 2-fold serial dilution of Tetra$_n$ from ˜2.8 mg/ml to ˜0.1 µg/ml, for a total of 16 samples per series, in 50 mM sodium phosphate, 150 mM NaCl, pH 7.0. Each sample of the series was loaded on a standard-coated MST capillary and placed on a Monolith NT.115 (NanoTemper Technologies). All experiments were performed in triplicate (values for each individual replicate are shown). Capillary scans across each series of 16 capillaries were performed prior to the application of the temperature gradient. LED power for each set of experiment was chosen in order to obtain maximum initial fluorescence values between 200 and 2,500 counts for every capillary of each individual series. Thermophoresis was analysed at the steady-state region of each

thermogram, after ˜30 s from the application of the temperature gradient (MST power chosen for every individual experiment is indicated to the left of each table). Curve fit values shown in graphs in Fig. 2F are indicated in "F_{norm} fit values" for BamA P3,4. For BamA P4,5, which exhibited variations in initial fluorescence greater than ± 10% of the average fluorescence along the serial dilution prior to the application of the temperature gradient (SI Fig. 2C), curve fit was performed directly on the initial fluorescence ("raw fluorescence fit values").

SI Table 6. Source data for MST analysis of interactions between Bam proteins and Tetra*n* (Extended Data Fig. 6). Data for each individual experiment shown in Extended Data Fig. 6 are indicated on separate sheets of the source data file; each sheet is named after the corresponding panel on Extended Data Fig. 6. Proteins labelled with Red-NHS or free Red-NHS in the absence of protein were incubated with a 2-fold serial dilution of Tetra*n* from ˜2.8 mg/ml to ˜0.1 µg/ml or mock PG digest diluted in the same way, for a total of 16 samples per series, in 50 mM sodium phosphate, 150 mM NaCl, pH 7.0. Each sample of the series was loaded on a standard-coated MST capillary and placed on a Monolith NT.115 (NanoTemper Technologies). All experiments were performed in triplicate (values for each individual replicate are shown). Capillary scans across each series of 16 capillaries were performed prior to the application of the temperature gradient. LED power for each set of experiment was chosen in order to obtain maximum initial fluorescence values between 200 and 2,500 counts for every capillary of each individual series. Thermophoresis was analysed at the steady-state region of each thermogram, after ˜30 s from the application of the temperature gradient (MST power chosen for every individual experiment is indicated to the left of each table). Curve fit values shown in Extended Data Fig. 6 are indicated in "F_{norm} fit values" for BamA P3,4 ("ED Fig. 6D" sheet), BamB ("ED Fig. 6F" sheet) and BamCD ("ED Fig. 6I" and "ED Fig. 6K" sheets).

Alternatively, for BamA P4,5 (“ED Fig. 6E” sheet), BamC (“ED Fig. 6G” sheet) and BamE (“ED Fig. 6H” sheet), which exhibited variations in initial fluorescence greater than ± 10% of the average fluorescence along the serial dilution prior to the application of the temperature gradient, curve fit was performed directly on the initial fluorescence (“raw fluorescence fit values”).

SI Table 7. Source data for initial fluorescence, MST traces, capillary overlay and SD-tests of interactions between Bam proteins and Tetra*n* (SI Fig. 2). Data for each individual experiment shown in SI Fig. 2 are indicated on separate sheets of the source data file; each sheet is named after the corresponding panel on SI Fig. 2. Proteins labelled with Red-NHS or free Red-NHS in the absence of protein were incubated with a 2-fold serial dilution of Tetra*n* from ˜2.8 mg/ml to ˜0.1 µg/ml or mock PG digest diluted in the same way, for a total of 16 samples per series, in 50 mM sodium phosphate, 150 mM NaCl, pH 7.0. Each sample of the series was loaded on a standard-coated MST capillary and placed on a Monolith NT.115 (NanoTemper Technologies). All experiments were performed in triplicate (values for each individual replicate are shown). Capillary scans across each series of 16 capillaries were performed prior to the application of the temperature gradient. LED power for each set of experiment was chosen in order to obtain maximum initial fluorescence values between 200 and 2,500 counts for every capillary of each individual series (“Initial fluorescence” sheets). LED power chosen for each experiment is indicated to the left of each table (graphs for maximum initial fluorescence measured for each capillary are shown in SI Fig. 2 A-J, “Initial fluorescence” graphs). Normalised fluorescence measured across the diameter of each capillary is shown in “Capillary overlay” sheets (graphs for capillary overlays are shown in SI Fig. 2 A-J, “Capillary overlay” graphs). Thermophoresis was monitored for ~5 s before switching on the infrared

laser to establish the microscopic temperature gradient within the capillary, then for 30 s during the application of the laser, then for ~5 s more after the laser was switched off ("MST traces" sheets). MST power chosen for each experiment is indicated on the left of each table (thermograms for normalised fluorescence throughout the experiment are shown in SI Fig. 2 A-J, "MST traces" graphs). Variations in initial fluorescence greater than ± 10% of the average fluorescence along the series prior to the application of the temperature gradient for BamA P4,5 (SI Fig. 2C), BamC (SI Fig. 2E) and BamE (SI Fig. 2F) were confirmed to be ligand-dependent by SD-tests: the raw fluorescence of the first three (bound state) and last three (unbound state) capillaries of each series was measured, then mixtures denatured in 2× SD-mix at 95°C and fluorescence measured again (see Methods). Data from SD-tests are indicated in "SD-test" sheets (results for SD-tests are shown in SI Fig. 2C, 2E and 2F, bottom graphs).

SI Table 8. Source data for BAM activity assays in the presence of tetrapeptide-rich or pentapeptide-rich PG (Fig. 3 C-E). BAM proteoliposomes were incubated with a serial 2-fold dilution of tetrapeptide-rich PG from MC1061 (Fig. 3C) or pentapeptide-rich PG from CS703-1 (Fig. 3D) in the presence of the fluorogenic peptide. Reactions were initiated by mixing with subreactions containing SurA and unfolded OmpT and immediately incubated at 30°C on a FLUOstar Microplate Reader (BMG Labtech). The fluorescent emission upon cleavage of the fluorogenic peptide by folded OmpT (excitation at 330 nm, emission at 430 nm) was monitored for 1 h 20 min with readings every 20 s. Raw fluorescence data from three replicates performed in the presence of different concentrations of tetrapeptide-rich PG ("Fig. 3C" sheet) or pentapeptide-rich PG ("Fig. 3D" sheet) are indicated. Activity rates relative to control reactions with no PG calculated from serial dilution experiments were plotted against

PG concentration (Fig. 3E) and curve fitting performed with the online MyCurve tool (https://mycurvefit.com/), using a symmetrical sigmoidal model ("Fig. 3E" sheet).

SI Table 9. Source data for *in vitro* BAM activity controls (Extended Data Fig. 7). Control reactions containing BAM proteoliposomes were incubated with the fluorogenic peptide. Reactions were initiated by mixing with subreactions containing SurA and unfolded OmpT. Alternatively, parallel reactions with no SurA, no OmpT or empty liposomes (no BAM) were prepared in the same way (Extended Data Fig. 7C). The fluorescent emission upon cleavage of the fluorogenic peptide (excitation at 330 nm, emission at 430 nm) was monitored for 1 h 20 min with readings every 20 s. Raw fluorescence data from three replicates are indicated ("ED Fig. 7C" sheet).

To analyse the effect of PG on the cleavage of the fluorogenic peptide by OmpT (Extended Data Fig. 7D), BAM proteoliposomes were mixed with SurA and unfolded OmpT and incubated for 2 h 30 min at 30°C, then mixtures supplemented either with fluorogenic peptide or with fluorogenic peptide and tetrapeptide-rich PG, and fluorescent emission monitored as described. Raw fluorescence data from three replicates are indicated ("ED Fig. 7D" sheet).

To study the effect of PG binding by SurA on *in vitro* BAM activity (Extended Data Fig. 7F), 15 µM SurA was incubated with 1 mg of tetrapeptide-rich PG, then the mixture supplemented to BAM activity assay reactions prepared as described. Parallel control reaction contained no excess SurA and no PG, excess SurA only, or PG only. Fluorescent emission was monitored as described. Raw fluorescence data from three replicates are indicated ("ED Fig. 7F" sheet).

BAM activity assays were also performed in the presence of $Tetra_n$ (Extended Data Fig. 7G) by incubating BAM proteoliposomes with 2.5 mg/ml or no $Tetra_n$ in the presence of the fluorogenic peptide, mixing with subreactions containing SurA and unfolded OmpT and

monitoring the fluorescent emission as described. Raw fluorescence data from three replicates are indicated (“ED Fig. 7F” sheet).

SI Table 10. Source data for BAM activity assays in the presence of PG isolated upon PBP5 complementation in CS703-1 (Extended Data Fig. 10E). PG was isolated from *E. coli* CS703-1 carrying p*dacA* upon arabinose induction of *dacA* expression, from CS703-1 and from the isogenic tetrapeptide-rich strain CS109. BAM proteoliposomes were incubated with no PG or with PG isolated from each of the three strains in the presence of the fluorogenic peptide. Reactions were initiated by mixing with subreactions containing SurA and unfolded OmpT and immediately incubated at 30°C on a FLUOstar Microplate Reader (BMG Labtech). The fluorescent emission upon cleavage of the fluorogenic peptide by folded OmpT (excitation at 330 nm, emission at 430 nm) was monitored for 1 h 20 min with readings every 20 s. Raw fluorescence data from three replicates performed in the presence of different concentrations of tetrapeptide-rich PG.

Supplementary Table References

64. Datsenko, K. A. & Wanner, B. L. One-step inactivation of chromosomal genes in *Escherichia coli* K-12 using PCR products. Proc. Natl. Acad. Sci. U. S. A. 97, 6640–6645 (2000).
65. Casadaban, M. J. & Cohen, S. N. Analysis of gene control signals by DNA fusion and cloning in *Escherichia coli*. J. Mol. Biol. 138, 179–207 (1980).
66. Heller, K., Mann, B. J. & Kadner, R. J. Cloning and expression of the gene for the vitamin B12 receptor protein in the outer membrane of *Escherichia coli*. J. Bacteriol. 161, 896–903 (1985).

67. de Boer, P. A. J., Crossley, R. E. & Rothfield, L. I. A division inhibitor and a topological specificity factor coded for by the minicell locus determine proper placement of the division septum in *E. coli*. Cell 56, 641–649 (1989).

68. Loraine, J. *et al*. Complement susceptibility in relation to genome sequence of recent *Klebsiella pneumoniae* isolates from Thai hospitals. Am Soc Microbiol 3, e00537-18 (2018).

69. Jacobs, M. A. *et al*. Comprehensive transposon mutant library of *Pseudomonas aeruginosa*. Natl. Acad Sci. 100, 14339–14344 (2003).

70. Housden, N. G. *et al*. Intrinsically disordered protein threads through the bacterial outer-membrane porin OmpF. Science 340, 1570–1574 (2013).

71. Rossiter, A. E. *et al*. The essential β-barrel assembly machinery complex components BamD and BamA are required for autotransporter biogenesis. J. Bacteriol. 193, 4250–4253 (2011).

72. Knowles, T. J., Scott-Tucker, A., Overduin, M. & Henderson, I. R. Membrane protein architects: The role of the BAM complex in outer membrane protein assembly. Nat. Rev. Microbiol. 7, 206–214 (2009).

73. Knowles, T. J. *et al*. Structure and function of BamE within the outer membrane and the β-barrel assembly machine. EMBO Rep. 12, 123–128 (2011).

74. Sklar, J. G. et al. Lipoprotein SmpA is a component of the YaeT complex that assembles outer membrane proteins in *Escherichia coli*. Proc. Natl. Acad. Sci. U. S. A. 21, 2473–2484 (2007).

75. Guzman, L. M., Belin, D., Carson, M. J. & Beckwith, J. Tight regulation, modulation, and high-level expression by vectors containing the arabinose P(BAD) promoter. J. Bacteriol. 177, 4121–4130 (1995).

76. Rodríguez-Alonso, R. *et al*. Structural insight into the formation of lipoprotein-β-barrel complexes. Nat. Chem. Biol. 16, 1019–1025 (2020).

77. Asmar, A. T. *et al*. Communication across the bacterial cell envelope depends on the size of the periplasm. PLoS Biol. 15, 1–16 (2017).
