# Supplementary figures and images for: Peptidoglycan maturation controls outer membrane protein assembly
Source: Nature. 2022 Jun 15;606(7916):953–9. doi: 10.1038/s41586-022-04834-7 (PMC9242858; doi:10.1038/s41586-022-04834-7)

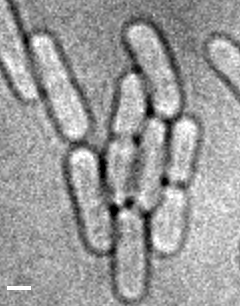

Supplement: Supplementary file 4 — This zipped file contains image source data. [file 41586_2022_4834_MOESM4_ESM.zip › Ext1/B1.tif]

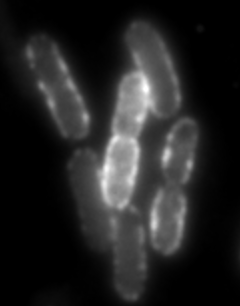

Supplement: Supplementary file 4 — This zipped file contains image source data. [file 41586_2022_4834_MOESM4_ESM.zip › Ext1/B2.tif]

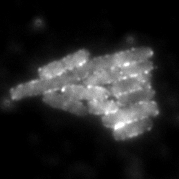

Supplement: Supplementary file 4 — This zipped file contains image source data. [file 41586_2022_4834_MOESM4_ESM.zip › Ext1/C1.tif]

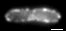

Supplement: Supplementary file 4 — This zipped file contains image source data. [file 41586_2022_4834_MOESM4_ESM.zip › Ext1/C3.jpg]

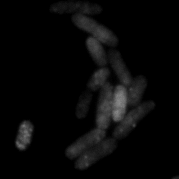

Supplement: Supplementary file 4 — This zipped file contains image source data. [file 41586_2022_4834_MOESM4_ESM.zip › Ext1/C4.tif]

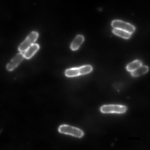

Supplement: Supplementary file 4 — This zipped file contains image source data. [file 41586_2022_4834_MOESM4_ESM.zip › Ext10/A1.tif]

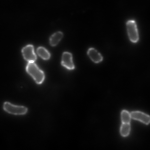

Supplement: Supplementary file 4 — This zipped file contains image source data. [file 41586_2022_4834_MOESM4_ESM.zip › Ext10/A2.tif]

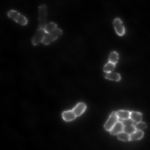

Supplement: Supplementary file 4 — This zipped file contains image source data. [file 41586_2022_4834_MOESM4_ESM.zip › Ext10/A3.tif]

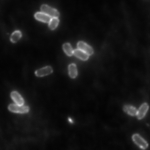

Supplement: Supplementary file 4 — This zipped file contains image source data. [file 41586_2022_4834_MOESM4_ESM.zip › Ext10/A4.tif]

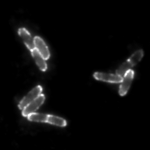

Supplement: Supplementary file 4 — This zipped file contains image source data. [file 41586_2022_4834_MOESM4_ESM.zip › Ext10/G1.tif]

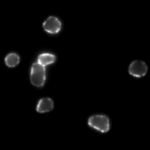

Supplement: Supplementary file 4 — This zipped file contains image source data. [file 41586_2022_4834_MOESM4_ESM.zip › Ext10/G2.tif]

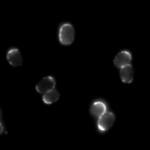

Supplement: Supplementary file 4 — This zipped file contains image source data. [file 41586_2022_4834_MOESM4_ESM.zip › Ext10/G3.tif]

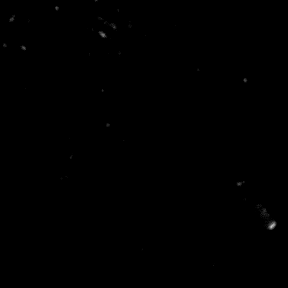

Supplement: Supplementary file 4 — This zipped file contains image source data. [file 41586_2022_4834_MOESM4_ESM.zip › Ext2/A1.tif]

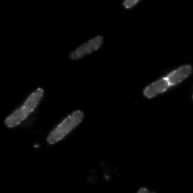

Supplement: Supplementary file 4 — This zipped file contains image source data. [file 41586_2022_4834_MOESM4_ESM.zip › Ext2/A2.tif]

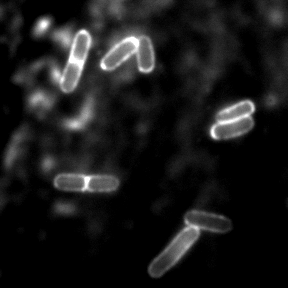

Supplement: Supplementary file 4 — This zipped file contains image source data. [file 41586_2022_4834_MOESM4_ESM.zip › Ext2/A3.tif]

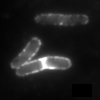

Supplement: Supplementary file 4 — This zipped file contains image source data. [file 41586_2022_4834_MOESM4_ESM.zip › Ext2/B1.tif]

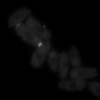

Supplement: Supplementary file 4 — This zipped file contains image source data. [file 41586_2022_4834_MOESM4_ESM.zip › Ext2/B2.tif]

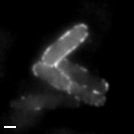

Supplement: Supplementary file 4 — This zipped file contains image source data. [file 41586_2022_4834_MOESM4_ESM.zip › Ext2/C1.tif]

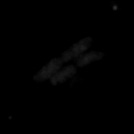

Supplement: Supplementary file 4 — This zipped file contains image source data. [file 41586_2022_4834_MOESM4_ESM.zip › Ext2/C2.tif]

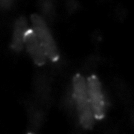

Supplement: Supplementary file 4 — This zipped file contains image source data. [file 41586_2022_4834_MOESM4_ESM.zip › Ext2/C3.tif]

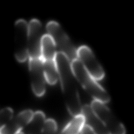

Supplement: Supplementary file 4 — This zipped file contains image source data. [file 41586_2022_4834_MOESM4_ESM.zip › Ext2/C4.tif]

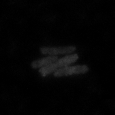

Supplement: Supplementary file 4 — This zipped file contains image source data. [file 41586_2022_4834_MOESM4_ESM.zip › Ext2/D1.tif]

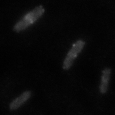

Supplement: Supplementary file 4 — This zipped file contains image source data. [file 41586_2022_4834_MOESM4_ESM.zip › Ext2/D2.tif]

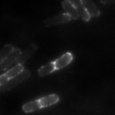

Supplement: Supplementary file 4 — This zipped file contains image source data. [file 41586_2022_4834_MOESM4_ESM.zip › Ext2/D3.tif]

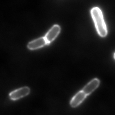

Supplement: Supplementary file 4 — This zipped file contains image source data. [file 41586_2022_4834_MOESM4_ESM.zip › Ext2/D4.tif]

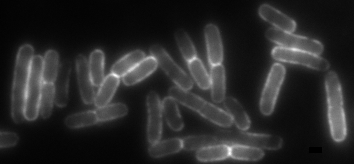

Supplement: Supplementary file 4 — This zipped file contains image source data. [file 41586_2022_4834_MOESM4_ESM.zip › Ext2/F1.tif]

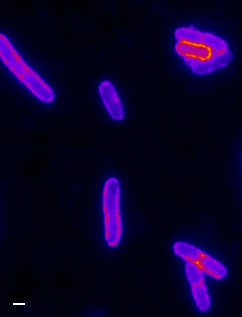

Supplement: Supplementary file 4 — This zipped file contains image source data. [file 41586_2022_4834_MOESM4_ESM.zip › Ext2/G.tif]

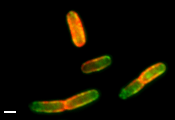

Supplement: Supplementary file 4 — This zipped file contains image source data. [file 41586_2022_4834_MOESM4_ESM.zip › Ext3/A.tif]

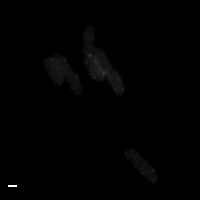

Supplement: Supplementary file 4 — This zipped file contains image source data. [file 41586_2022_4834_MOESM4_ESM.zip › Ext3/B1.tif]

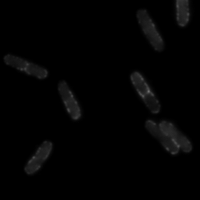

Supplement: Supplementary file 4 — This zipped file contains image source data. [file 41586_2022_4834_MOESM4_ESM.zip › Ext3/B2.tif]

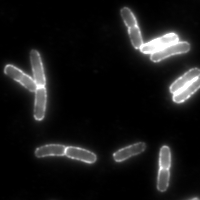

Supplement: Supplementary file 4 — This zipped file contains image source data. [file 41586_2022_4834_MOESM4_ESM.zip › Ext3/B3.tif]

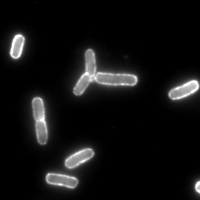

Supplement: Supplementary file 4 — This zipped file contains image source data. [file 41586_2022_4834_MOESM4_ESM.zip › Ext3/B4.tif]

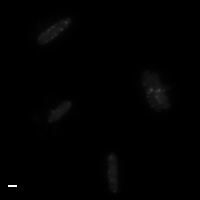

Supplement: Supplementary file 4 — This zipped file contains image source data. [file 41586_2022_4834_MOESM4_ESM.zip › Ext3/D1.tif]

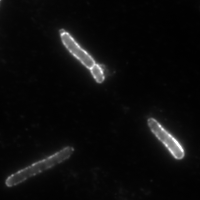

Supplement: Supplementary file 4 — This zipped file contains image source data. [file 41586_2022_4834_MOESM4_ESM.zip › Ext3/D2.tif]

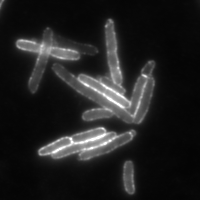

Supplement: Supplementary file 4 — This zipped file contains image source data. [file 41586_2022_4834_MOESM4_ESM.zip › Ext3/D3.tif]

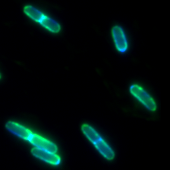

Supplement: Supplementary file 4 — This zipped file contains image source data. [file 41586_2022_4834_MOESM4_ESM.zip › Ext4/G3.tif]

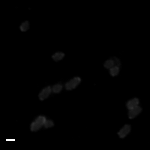

Supplement: Supplementary file 4 — This zipped file contains image source data. [file 41586_2022_4834_MOESM4_ESM.zip › Ext5/A1.tif]

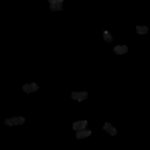

Supplement: Supplementary file 4 — This zipped file contains image source data. [file 41586_2022_4834_MOESM4_ESM.zip › Ext5/A2.tif]

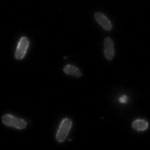

Supplement: Supplementary file 4 — This zipped file contains image source data. [file 41586_2022_4834_MOESM4_ESM.zip › Ext5/A3.tif]

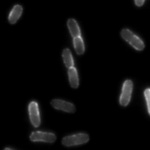

Supplement: Supplementary file 4 — This zipped file contains image source data. [file 41586_2022_4834_MOESM4_ESM.zip › Ext5/A4.tif]

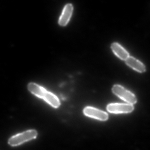

Supplement: Supplementary file 4 — This zipped file contains image source data. [file 41586_2022_4834_MOESM4_ESM.zip › Ext5/A5.tif]

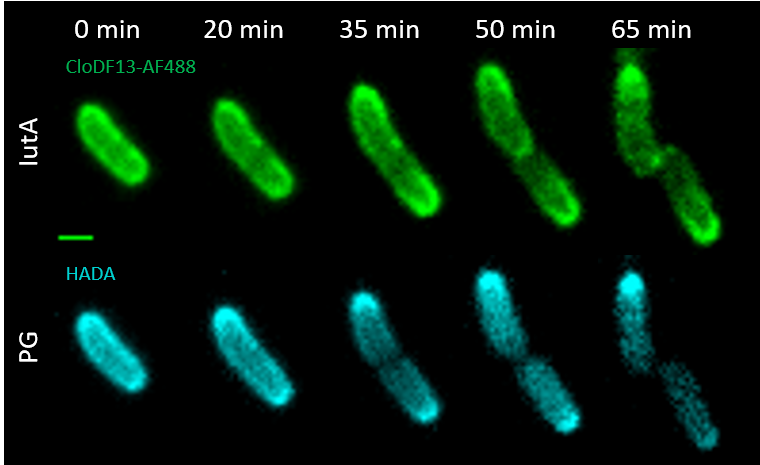

Supplement: Supplementary file 4 — This zipped file contains image source data. [file 41586_2022_4834_MOESM4_ESM.zip › Ext5/G.png]

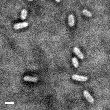

Supplement: Supplementary file 4 — This zipped file contains image source data. [file 41586_2022_4834_MOESM4_ESM.zip › Ext5/H1.tif]

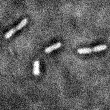

Supplement: Supplementary file 4 — This zipped file contains image source data. [file 41586_2022_4834_MOESM4_ESM.zip › Ext5/H5.tif]

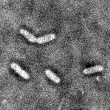

Supplement: Supplementary file 4 — This zipped file contains image source data. [file 41586_2022_4834_MOESM4_ESM.zip › Ext5/H9.tif]

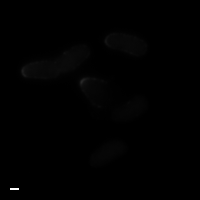

Supplement: Supplementary file 4 — This zipped file contains image source data. [file 41586_2022_4834_MOESM4_ESM.zip › Ext8/A1.tif]

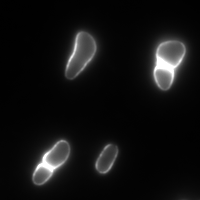

Supplement: Supplementary file 4 — This zipped file contains image source data. [file 41586_2022_4834_MOESM4_ESM.zip › Ext8/A2.tif]

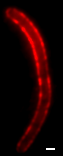

Supplement: Supplementary file 4 — This zipped file contains image source data. [file 41586_2022_4834_MOESM4_ESM.zip › Ext8/C1.tif]

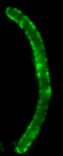

Supplement: Supplementary file 4 — This zipped file contains image source data. [file 41586_2022_4834_MOESM4_ESM.zip › Ext8/C2.tif]

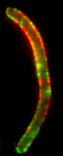

Supplement: Supplementary file 4 — This zipped file contains image source data. [file 41586_2022_4834_MOESM4_ESM.zip › Ext8/C3.tif]

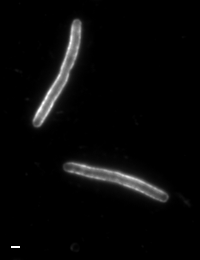

Supplement: Supplementary file 4 — This zipped file contains image source data. [file 41586_2022_4834_MOESM4_ESM.zip › Ext8/E1.tif]

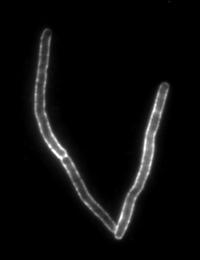

Supplement: Supplementary file 4 — This zipped file contains image source data. [file 41586_2022_4834_MOESM4_ESM.zip › Ext8/E2.tif]

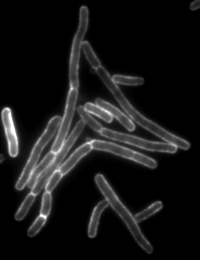

Supplement: Supplementary file 4 — This zipped file contains image source data. [file 41586_2022_4834_MOESM4_ESM.zip › Ext8/E3.tif]

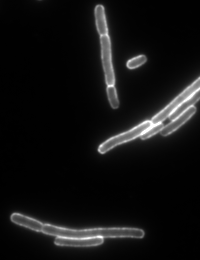

Supplement: Supplementary file 4 — This zipped file contains image source data. [file 41586_2022_4834_MOESM4_ESM.zip › Ext8/E4.tif]

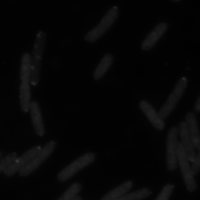

Supplement: Supplementary file 4 — This zipped file contains image source data. [file 41586_2022_4834_MOESM4_ESM.zip › Ext9/A1.tif]

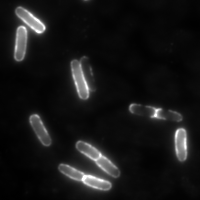

Supplement: Supplementary file 4 — This zipped file contains image source data. [file 41586_2022_4834_MOESM4_ESM.zip › Ext9/A2.tif]

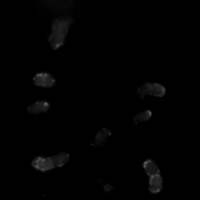

Supplement: Supplementary file 4 — This zipped file contains image source data. [file 41586_2022_4834_MOESM4_ESM.zip › Ext9/A3.tif]

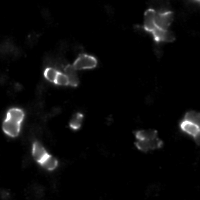

Supplement: Supplementary file 4 — This zipped file contains image source data. [file 41586_2022_4834_MOESM4_ESM.zip › Ext9/A4.tif]

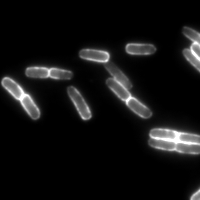

Supplement: Supplementary file 4 — This zipped file contains image source data. [file 41586_2022_4834_MOESM4_ESM.zip › Ext9/B1.tif]

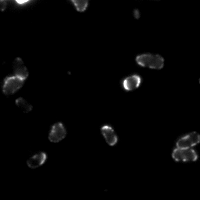

Supplement: Supplementary file 4 — This zipped file contains image source data. [file 41586_2022_4834_MOESM4_ESM.zip › Ext9/B4.tif]

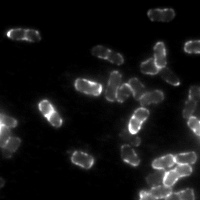

Supplement: Supplementary file 4 — This zipped file contains image source data. [file 41586_2022_4834_MOESM4_ESM.zip › Ext9/B5.tif]

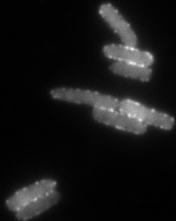

Supplement: Supplementary file 4 — This zipped file contains image source data. [file 41586_2022_4834_MOESM4_ESM.zip › Ext9/F1.tif]

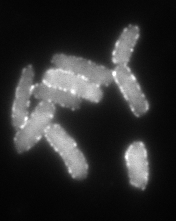

Supplement: Supplementary file 4 — This zipped file contains image source data. [file 41586_2022_4834_MOESM4_ESM.zip › Ext9/F3.tif]

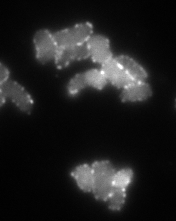

Supplement: Supplementary file 4 — This zipped file contains image source data. [file 41586_2022_4834_MOESM4_ESM.zip › Ext9/F5.tif]

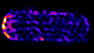

Supplement: Supplementary file 4 — This zipped file contains image source data. [file 41586_2022_4834_MOESM4_ESM.zip › Fig1/A1.tif]

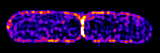

Supplement: Supplementary file 4 — This zipped file contains image source data. [file 41586_2022_4834_MOESM4_ESM.zip › Fig1/A2.tif]

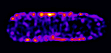

Supplement: Supplementary file 4 — This zipped file contains image source data. [file 41586_2022_4834_MOESM4_ESM.zip › Fig1/A3.tif]

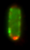

Supplement: Supplementary file 4 — This zipped file contains image source data. [file 41586_2022_4834_MOESM4_ESM.zip › Fig1/C3.tif]

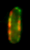

Supplement: Supplementary file 4 — This zipped file contains image source data. [file 41586_2022_4834_MOESM4_ESM.zip › Fig1/C6.tif]

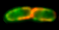

Supplement: Supplementary file 4 — This zipped file contains image source data. [file 41586_2022_4834_MOESM4_ESM.zip › Fig1/C9.tif]

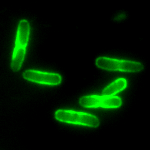

Supplement: Supplementary file 4 — This zipped file contains image source data. [file 41586_2022_4834_MOESM4_ESM.zip › Fig2/A1.tif]

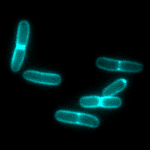

Supplement: Supplementary file 4 — This zipped file contains image source data. [file 41586_2022_4834_MOESM4_ESM.zip › Fig2/A2.tif]

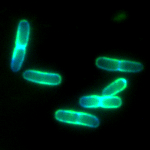

Supplement: Supplementary file 4 — This zipped file contains image source data. [file 41586_2022_4834_MOESM4_ESM.zip › Fig2/A3.tif (RGB).tif]

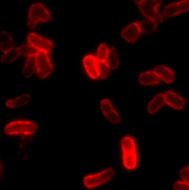

Supplement: Supplementary file 4 — This zipped file contains image source data. [file 41586_2022_4834_MOESM4_ESM.zip › Fig4/A1.tif]

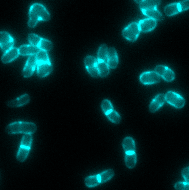

Supplement: Supplementary file 4 — This zipped file contains image source data. [file 41586_2022_4834_MOESM4_ESM.zip › Fig4/A2.tif]

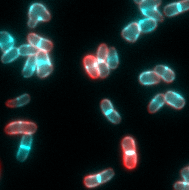

Supplement: Supplementary file 4 — This zipped file contains image source data. [file 41586_2022_4834_MOESM4_ESM.zip › Fig4/A3.tif]

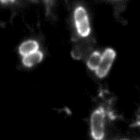

Supplement: Supplementary file 4 — This zipped file contains image source data. [file 41586_2022_4834_MOESM4_ESM.zip › Fig4/C1.tif]

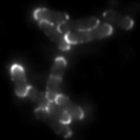

Supplement: Supplementary file 4 — This zipped file contains image source data. [file 41586_2022_4834_MOESM4_ESM.zip › Fig4/C2.tif]

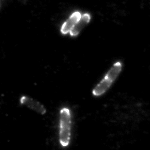

Supplement: Supplementary file 4 — This zipped file contains image source data. [file 41586_2022_4834_MOESM4_ESM.zip › Fig4/D1.tif]

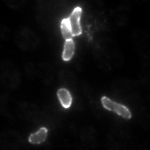

Supplement: Supplementary file 4 — This zipped file contains image source data. [file 41586_2022_4834_MOESM4_ESM.zip › Fig4/D2.tif]

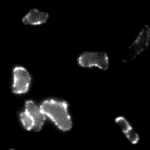

Supplement: Supplementary file 4 — This zipped file contains image source data. [file 41586_2022_4834_MOESM4_ESM.zip › Fig4/D3.tif]

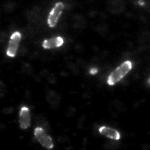

Supplement: Supplementary file 4 — This zipped file contains image source data. [file 41586_2022_4834_MOESM4_ESM.zip › Fig4/D4.tif]
